# DxDirector: an agentic large language model driving the full-process clinical diagnosis

**DOI:** 10.1038/s41467-026-71928-5

**Published:** 2026-04-23

**Authors:** Shicheng Xu, Xin Huang, Zihao Wei, Liang Pang, Huawei Shen, Xueqi Cheng

**Affiliations:** 1 State Key Laboratory of AI Safety, Institute of Computing Technology, Chinese Academy of Sciences, Beijing, China; 2 University of Chinese Academy of Sciences, Beijing, China; 3 Peking University Third Hospital, Beijing, China

**Keywords:** Diagnosis, Predictive medicine, Medical research

## Abstract

Clinical diagnosis in the real world often begins with ambiguous patient complaints that require iterative reasoning and testing. While large language models (LLMs) increasingly assist with specific medical queries, they currently lack the ability to autonomously drive this entire diagnostic workflow, limiting their potential to significantly alleviate physician workload. Here we present DxDirector-7B, an agentic LLM designed to navigate the full diagnostic process through advanced slow thinking capabilities. Unlike existing assistants, our model autonomously determines optimal diagnostic strategies, requesting physician intervention only for necessary clinical operations. In evaluations spanning rare diseases and complex real-world cases, DxDirector-7B achieves superior diagnostic accuracy compared to state-of-the-art medical and general-purpose LLMs with significantly larger parameters. Crucially, it drastically reduces physician involvement while maintaining a robust safety and accountability framework for high-risk conditions. These results demonstrate a paradigm shift where AI effectively leads clinical reasoning, offering a scalable solution to enhance diagnostic efficiency and accessibility.

## Abstract (web summary)

Authors develop an autonomous agentic LLM called DxDirector-7B. Its clinical diagnostic accuracy outperforms larger models. Using deep thinking strategies, it significantly reduces physician workload, requesting human assistance only when necessary.

## Introduction

Full-process clinical diagnosis encompasses the entire diagnostic workflow connected with clinical decision making^1^, beginning with a patient’s vague chief complaint. Physicians must iteratively make differential diagnoses, design and interpret a series of appropriate diagnostic tests, and progressively refine their understanding of the patient’s clinical information before reaching a definitive diagnosis^2–4^. This complex process demands not only extensive medical knowledge and advanced reasoning skills^5^ but also imposes a substantial workload on physicians. Despite rigorous professional training, the misdiagnosis rate in clinical practice remains close to 20%^6,7^. The growing patient demand continues to outpace the diagnostic capacity of physicians, underscoring the urgent need for more efficient and scalable diagnostic solutions^8,9^.

Recent advances in large language models (LLMs), a rapidly evolving artificial intelligence (AI) technology, have demonstrated remarkable capabilities in language comprehension and generation. This progress has spurred growing interest in their potential applications in clinical diagnosis. Emerging studies suggest that LLMs exhibit promising diagnostic performance^10–13^, prompting the development of medical-specialized LLMs^7,14–19^. However, the role of LLMs in real-world diagnosis is limited to functioning merely as tools for physicians. This limitation arises primarily because current LLMs excel in diagnosing cases with comprehensive clinical data—such as symptoms, medical history, diagnostic test results, and  clear instructions^7,15,17^—whereas real-world full-process clinical diagnosis often begins with only a patient’s vague chief complaint^2,4,20^. This means LLMs can only provide assistance in making final diagnoses or answering specific medical questions at certain parts within the diagnostic process, while the majority of the diagnostic process still relies heavily on human physicians, such as clinical reasoning, condition assessment, and designing diagnostic tests to progressively enrich the clinical information.

**Fig. 1:**
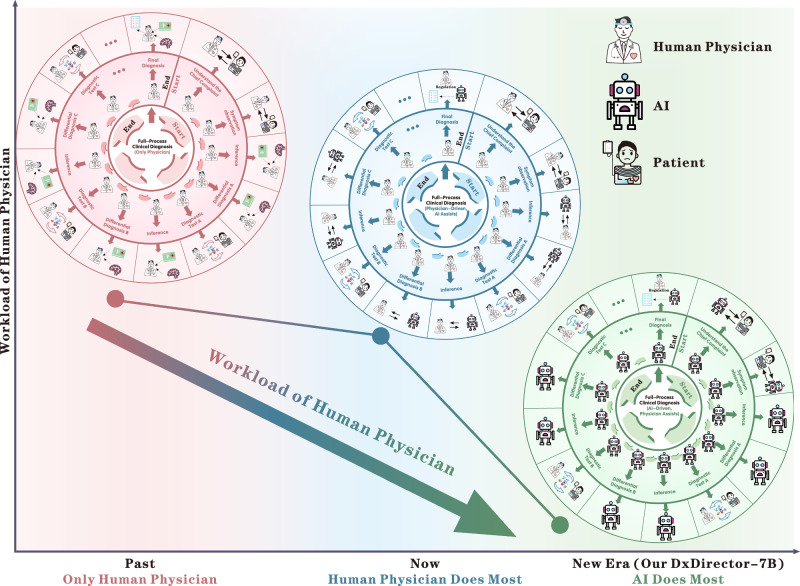
Workflow of full-process diagnosis in past, now and ours. The inner circle represents the driver (deciding the specific clinical problems) of multi-step dynamic clinical diagnosis, and the outer circle represents the specific execution of the corresponding steps (solving the clinical problems). In the past, all work is done by human physicians. Now, artificial intelligence (AI), especially large language model (LLM), has reduced physicians' workload to a certain extent, but AI can only serve as a tool to answer questions designed by physicians at specific steps, and lacks the ability to drive the full diagnosis process starting from the chief complaint, which still relies heavily on physicians. DxDirector-7B, marks an era that AI can drive the full-process diagnosis, only needs physicians at necessary steps, reducing the workload of physicians as much as possible.

To address this challenge, we propose a paradigm shift in the role of LLMs in clinical diagnosis. Unlike existing LLMs, which function solely as specific assistants to physicians, our approach redefines this relationship by positioning the LLM as a collaborative tool for physicians that can drive the full diagnostic process to significantly reduce physician workload on repetitive and low-risk reasoning tasks and allowing physicians to primarily focus on clinical operations, risk management and final decision. As described in Fig. 1, at the beginning, the LLM can only access the patient’s vague chief complaint, and it gradually clarifies the patient’s condition, designs appropriate diagnostic tests, infers complex medical knowledge and clinical phenomena, and finally makes the diagnosis. During this process, the LLM can dynamically request assistance from physicians only when it faces clinical operations that computer program-based LLMs cannot complete, such as symptom observation, laboratory testing, physical examination, etc. Physicians fulfill these requests and input the results, enabling the LLM to continue the subsequent diagnosis. The pattern of the LLM requesting assistance adheres to the principle of minimizing physicians’ involvement, thereby reducing the workload and requirement for medical expertise of physicians as much as possible. Furthermore, a risk identification model accompanies the entire diagnostic process and can automatically identify high risks that affect patient safety. It enables physicians to take over the diagnosis process at any time to avoid potential risks and retain the final decision-making power over the diagnosis results.

Building on this design, we introduce DxDirector-7B, an LLM with advanced deep thinking capabilities (like human “slow thinking”) by going through complex reasoning before providing the specific decisions, capable of autonomously driving the full-process clinical diagnosis starting from a vague chief complaint. DxDirector-7B progressively executes the entire clinical diagnosis step-by-step; it performs deep thinking to determine the optimal strategy at each step and seeks assistance from human physicians only at necessary steps with the principle of minimizing physicians’ involvement. It dynamically assesses whether sufficient clinical information has been gathered to establish a final diagnosis or whether further diagnostic steps are required. The final diagnostic output includes a summary of the entire diagnosis process, supported by authoritative medical literature, thereby enhancing the verifiability of AI-generated diagnoses. Furthermore, this structured output establishes a robust accountability framework between physicians and the LLM, ensuring traceability in misdiagnosis.

We evaluate DxDirector-7B in the full-process clinical diagnosis setting using both real-world scenarios and four authoritative publicly available datasets. The evaluation datasets comprise 26,018 cases, including rare, complex, and diagnostically challenging cases reported in New England Journal of Medicine (NEJM) Clinicopathologic Cases^21^, cases from the U.S. Medical Licensing Examination (USMLE)^22^, and cases of real-world inpatients from officially certified Grade 3A hospitals in China (Grade 3A hospitals are the highest level hospitals in China’s “three-grade, six-class” classification system). To ensure a comprehensive assessment, we conduct fine-grained evaluations across 19 clinical departments (e.g., neurosurgery, oncology, endocrinology) and 12 clinical tasks (e.g., diagnosis, differential diagnosis, treatment). Experimental results indicate that in terms of full-process clinical diagnostic accuracy, DxDirector-7B significantly surpasses medically adapted LLMs that use more parameters, such as MedFound-176B^7^ and OpenbioLLM-70B. It also significantly surpasses the current strongest commercial general-purpose LLMs with nearly 100 times more parameters, such as GPT-4o, o1-preview, o3-mini, and Deepseek-R1-671B. Notably, DxDirector-7B achieves this superior performance while requiring significantly lower physician involvement compared to these other LLMs. These findings show that DxDirector-7B achieves greater accuracy with significantly lower computational and training costs, requiring the lowest human physicians’ efforts in the entire diagnosis process. Evaluations with the participation of medical specialists show that in real-world full-process diagnostic scenarios, the diagnoses generated by DxDirector-7B achieve substitution for medical specialists in 60% to 75% of cases in many departments such as pulmonology and gastroenterology. Further analysis highlights its ability to provide a fine-grained verification framework for AI-generated diagnoses, establishing a robust accountability mechanism between physicians and AI in the event of misdiagnosis.

This paper marks an era where AI, traditionally a tool for physicians on specific questions, now leads the entire diagnostic process with minimal physician involvement. It advances effective AI deployment in full-process clinical workflows of the real world, reducing the workload of physicians to the greatest extent possible and indicating an efficient, accurate and scalable diagnostic solution.

## Results

In this section, we first give an overview of DxDirector-7B and its training method. Then, we present the experimental results about comparing DxDirector-7B with the most advanced medically adapted LLMs and commercial general-purpose LLMs.

### Overview of DxDirector-7B and training method

This section introduces the overview of our proposed DxDirector-7B and its training method.

#### Overview of DxDirector-7B

**Fig. 2:**
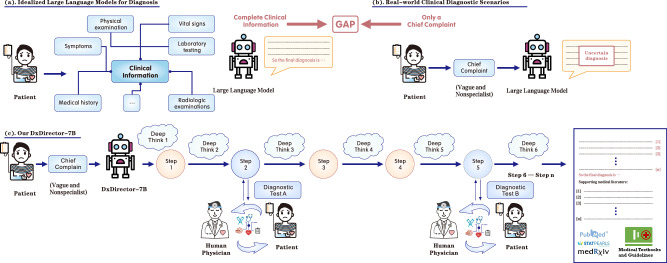
Comparison between DxDirector-7B and existing large language models (LLMs) in full-process clinic diagnosis. **a** Existing LLMs are still limited to answering questions with complete clinical information. **b** However, real-world clinical diagnosis only begins with the patient’s vague and nonspecialist chief complaint. This gap means that much more work in collecting complete clinical information still highly relies on human physicians. **c** DxDirector-7B addresses this by driving the full-process clinical diagnosis step-by-step, only requesting assistance from physicians at necessary steps with the principle of minimizing physicians' involvement.

The overall workflow of DxDirector-7B is shown in Fig. 2c and the practical case of DxDirector-7B is illustrated in Supplementary Fig. 1. As shown in Fig. 2a, b, current LLMs typically rely on complete clinical information for diagnosis, a scenario that rarely aligns with real-world clinical practice, where initial information often consists solely of a vague chief complaint. This means physicians still need to handle a heavy workload between receiving the chief complaint and making final diagnosis, and LLMs are just assistants in certain parts of this entire complex process. To address this disparity, we introduce DxDirector-7B, an advanced LLM with powerful deep thinking ability to drive the full-process clinical diagnosis starting with only a patient’s vague chief complaint, which is much closer to real-world clinical diagnosis than existing LLMs.

In Fig. 2c, DxDirector-7B systematically executes complex clinical diagnosis in a stepwise manner, interconnected by a detailed reasoning process—termed “deep thinking”—that mimics human “slow thinking” cognitive strategy. This deep thinking incorporates current clinical data and integrates overarching diagnostic objectives, guiding the identification of critical questions at each diagnostic stage (denoted as [Question] in Supplementary Fig. 1). If the question requires objective medical knowledge or inference (automatically marked with “<LLM>”), DxDirector-7B will generate the answer to the question by itself. If the question requires clinical operations for diagnostics, such as medical imaging, physical examinations, laboratory tests that computer program-based LLMs cannot complete, it will actively request assistance from human physicians (automatically marked with “<Physician>”), who will input the results of the operation as an answer. The deep thinking at each step ensures that the step is correct and efficient, so that the diagnosis can be completed accurately while relying on the minimal human physicians’ efforts.

When DxDirector-7B determines that the diagnosis is complete, it synthesizes the preceding steps into a succinct summary ([Final Diagnosis] in Supplementary Fig. 1). It can attach the authoritative medical literature retrieved by a medical search model or physicians’ operations involved in each step. This improves the verifiability of the generated diagnosis at fine-grained level. Additionally, it clearly delineates the actions of LLMs and physicians to establish a precise accountability framework, which is critical in potential medical incidents. More practical cases are in Supplementary Figs. 30–35.

Compared to existing state-of-the-art LLMs, DxDirector-7B achieves superior diagnostic accuracy while markedly reducing both the clinical workload and expertise required of human physicians. As discussed in Fig. 1, the development of DxDirector-7B signals a paradigm shift in clinical practice, fundamentally redefining the collaborative dynamics between AI and healthcare professionals and providing an efficient, accurate and scalable diagnostic solution.

#### Training method

Our training method for DxDirector-7B includes three stages: (1) Continued pre-training on medical data; (2) Instruction-tuning for full-process diagnosis; (3) Step-level strategy preference optimization.

The first stage is consistent with existing medical-adaptation methods for LLMs^7,17^. We continued pre-training Llama-2-7B^23^ on large-scale medical data such as clinical guidelines, PubMed papers, etc. This stage enables the general LLMs to acquire medical knowledge, which forms the foundation for its clinical diagnosis capabilities. Details about this can be found in section “Continued pre-training on medical data”.

The second stage is instruction-tuning for full-process clinical diagnosis. This stage enables DxDirector-7B to drive the full-process clinical diagnosis solely starting with ambiguous chief complaints, through the step-by-step reasoning and continuous deep thinking. The training dataset is constructed based on publicly available medical question-answering data^22^. We use general-purpose LLM GPT-4o to convert patients’ case reports in the datasets into step-by-step reasoning, and use powerful reasoning LLM o1-preview to enrich the thinking process of each step (details of this can be found in section “Instruction-tuning for full-process clinical diagnosis”). The automated construction process of this synthetic data is supervised by medical experts. Following data construction, we obtain 10,178 high-quality instruction-response pairs covering multiple clinical tasks such as diagnosis, differential diagnosis, designing treatment plan, screening, etiology analysis, etc. Instruction tuning based on this dataset endows DxDirector-7B with the preliminary capability to drive a full-process clinical diagnosis and perform deep thinking. The technical details of training can be found in section “Instruction-tuning for full-process clinical diagnosis”.

The third stage is step-level strategy preference optimization. We define the question to be solved in each step derived by deep thinking of DxDirector-7B as a “strategy”. After the second stage, DxDirector-7B can generate the strategy step-by-step as shown in Fig. 2c. The third stage enables DxDirector-7B to implicitly compare multiple potential strategies in deep thinking at each step and select the optimal strategy. This ensures that each step in complex clinical reasoning is correct and efficient, so that the diagnosis can be completed accurately while relying on minimal human physician effort. The optimization of this stage is performed at step-level. In training data construction, we use multiple sampling to make DxDirector-7B generate multiple different strategies for each step (given the same prefix) and assign different rewards to these strategies. The reward value is determined by both the correctness of the final answer and the quantified physician workload derived from the strategies. Strategies with more correct answers are assigned higher rewards. For strategies with the same correct answers, the strategies that seek more assistance from human physicians will have a lower reward value. In training, DxDirector-7B learns to refine deep thinking to generate the strategy with the highest reward by reward-based reinforcement learning^24^ with the principle of ensuring correctness while minimizing the workload of human physicians. Details can be found in section “Step-level strategy preference optimization”.

### Overview of experiments

#### Evaluation datasets

The evaluation datasets consist of two parts: one is four publicly available medical datasets evaluated automatically based on their provided correct answers, and the other is a set of cases in real-world clinical diagnosis with the evaluation participated by medical specialists.

For the publicly available datasets, we first collect raw data and then reconstruct them to simulate full-process clinical diagnosis scenarios. Four datasets are utilized: (1) RareArena (https://github.com/zhao-zy15/RareArena) is a dataset of nearly 50,000 rare disease diagnoses extracted from case summaries in PubMed Central, covering 4597 rare disease types. We use the rare disease confirmation subset, which covers 22,901 data samples. (2) NEJM Clinicopathologic Cases^21^, which covers 344 clinical cases published by the New England Journal of Medicine between 2014 and 2024. These cases are highly complex, rare, and educationally significant. (3) ClinicalBench^25^ is a multi-departmental clinical diagnostic evaluation benchmark including 1500 real-world cases that cover 150 diseases. (4) US Medical License Exam^22^, a set of 1273 challenging medical questions in the US Medical License Exam. There are many tasks in this dataset such as diagnosis, differential diagnosis, treatment planning, etc.

To simulate the full-process clinical diagnosis beginning with only a patient’s initial chief complaint, we reconstruct the four datasets as follows. For each data instance, we first employ GPT-4o API. to extract all clinical information (patient’s profile, disease symptoms and histories, drug dosage requirements, diagnostic test results, etc.). Next, we utilize GPT-4o API to transform medically precise clinical descriptions into vague chief complaints characteristic of real patients. Both steps leverage the in-context learning approach^26^, guided by explicit instructions and exemplar cases curated by medical experts. In this way, each data instance is reformulated as a triplet comprising a clinical diagnostic question, an initial patient chief complaint, and detailed clinical information. At the beginning of the diagnosis, LLMs can only access the chief complaint, while additional clinical information needs to be gradually inferred or obtained through its active reasoning.

For the real-world clinical diagnosis, we construct a real clinical diagnostic scenario within an officially certified Grade 3A hospital in China. This evaluation covers 160 real cases across 9 different clinical departments. We invited the medical specialists in each department to participate in the evaluation of the diagnostic results generated by LLMs. The specific details about this can be found in section “Evaluations on real-world clinical diagnosis”. To safeguard patient privacy, any personally identifiable information (PII) or other sensitive details have been manually identified and removed by the medical team.

#### Full-process clinical diagnosis setting for evaluation

Based on above datasets, we construct a full-process clinical diagnosis setting to evaluate the performance of various LLMs. In this setting, each data instance consists of a question, a patient’s chief complaint and detailed clinical information. Initially, the LLM has access solely to the chief complaint and is tasked with addressing questions related to diagnosis, treatment strategies, etiology, etc. Any additional clinical details must subsequently be inferred or actively obtained through stepwise reasoning. When encountering tasks that need clinical operations that the computer program cannot complete, such as symptom observation, laboratory testing, physical examination, etc, the LLM proactively requests assistance from human physicians. To automatically simulate this physician interaction on large-scale dataset, we implement an AI agent powered by GPT-4o, which receives real-time queries from the LLM, interprets the requested clinical information, and provides relevant data extracted from detailed clinical information to LLM, allowing LLM to continue reasoning (see prompts in Supplementary Fig. 21). This framework effectively replicates realistic interactions in full-process clinical diagnosis, where LLM asks the assistance from physicians. In order to make the baselines actively perform full-process clinical diagnosis rather than directly make a diagnosis when the clinical information is insufficient (as shown in Fig. 2a, b), we design specific prompts (Supplementary Figs. 22 and 23) to guide the baselines to engage in the multi-round conversation between the simulated physicians and clinical environments.

#### Baselines

The baselines in the experiments can be divided into two categories: One is the current most powerful commercial general-purpose LLMs including Deepseek-R1-671B^27^, Deepseek-v3-671B^27^, GPT-4o^28^, OpenAI o1-preview^29^, OpenAI o3-mini (https://openai.com/index/o3-mini-system-card/), Gemini-2.0-flash^30^. These LLMs boast hundreds of billions of parameters and are developed by tech giants (OpenAI, Google, Microsoft and Deepseek) at immense training costs. Recent study has shown that they have promising performance in making the final clinical diagnosis^21,31^. The other is the open source LLMs specifically optimized for medical domain including Meditron-70B^17^, OpenbioLLM-70B (https://huggingface.co/aaditya/Llama3-OpenBioLLM-70B#), Clinical Camel-70B^32^ and Meditron-176B^7^ and a open source general LLM Llama-3-70B^33^. They have significantly more parameters than DxDirector-7B (they have 70 billion parameters or 176 billion parameters, while DxDirector-7B has only 7 billion parameters), which means they are more expensive to train and infer.

### Accuracy of clinical diagnosis

This section reports the experimental results about clinical diagnostic accuracy of various LLMs on NEJM Clinicopathologic Cases, RareArena and ClinicalBench in full-process diagnosis setting. DxDirector-7B achieved the highest accuracy. The detailed results and analysis are as follows.

**Fig. 3:**
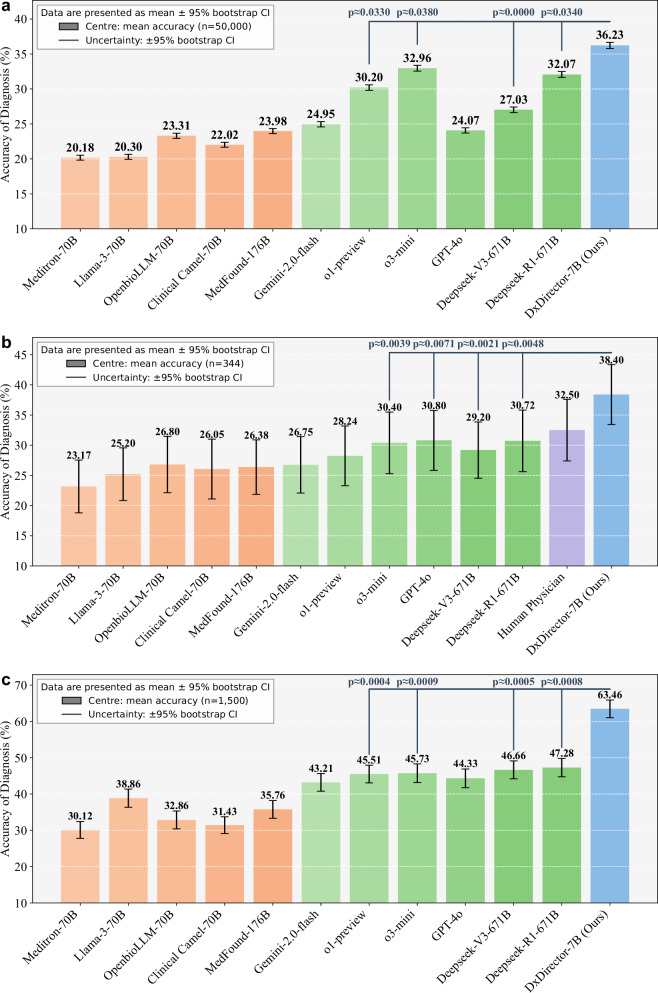
Accuracy of diagnoses generated by different large language models (LLMs) across different datasets in full-process diagnosis setting. **a** Results on dataset with Rare Disease Cases (RareArena). **b** Results on dataset with Complex Cases (New England Journal of Medicine Clinicopathologic Cases). **c** Results on dataset with Real-world Cases (ClinicalBench). Bars are annotated with the accuracy of each LLM. Error bars reflect 95% confidence intervals determined by non-parametric bootstrap procedure with 1000 samples on RareArena and ClinicalBench, and 200 samples on New England Journal of Medicine (NEJM) Cases. We perform statistical significance tests utilizing the two-side McNemar test between DxDirector-7B and the top-3 baselines on each dataset, with *p* value levels annotated on the bars. Source data are available in the Source Data file (Figure-3-a.xlsx, Figure-3-b.xlsx, Figure-3-c.xlsx).

The evaluation on RareArena reveals the capacity of LLMs to diagnose rare diseases—a challenging domain that requires expertise in conditions characterized by low prevalence, encompassing 4,597 distinct pathologies across 22,901 clinical cases. As illustrated in Fig. 3a, under the full-process diagnostic setting, DxDirector-7B achieves the highest accuracy at 36.23%. This represents a 3.27% absolute advantage over the strongest commercial LLM (o3-mini: 32.96%) and a 12.25% lead against medically adapted LLMs (MedFound-176B: 23.98%), despite using 25 times fewer parameters than medically adapted LLMs and nearly 100 times fewer than commercial LLMs like Deepseek-R1-671B (32.07%). Besides, the stark contrast between commercial LLMs highlights reasoning’s critical role—GPT-4o (24.07%) underperforms o3-mini by 8.89% and o1-preview (30.20%) by 6.13%, despite comparable medical knowledge memorization. Both o1-preview and o3-mini are LLMs with powerful reasoning ability. This suggests that stronger logical reasoning enables better synthesis of sparse symptom patterns in rare disease diagnosis, a capability GPT-4o lacks despite superior general intelligence^34^. DxDirector-7B amplifies this advantage through deep thinking like human at each step, achieving higher parameter efficiency while delivering higher accuracy.

The evaluation on NEJM Clinicopathologic Cases benchmark reveals critical insights into the capabilities and limitations of LLMs in complex clinical reasoning. Figure 3b presents the diagnostic accuracy of baselines and DxDirector-7B in the setting of full-process clinical diagnosis. DxDirector-7B achieves accuracy (38.4%) and outperforms human physicians. Further analysis reveals three pivotal findings. (1) First, existing medical-domain adaptation methods of

LLMs provides limited benefits on full-process diagnosis: LLMs pretrained on large-scale medical data (Meditron-70B: 23.17%; OpenbioLLM-70B: 26.80%; MedFound-176B: 26.38%) show marginal gains over the generalist Llama-3-70B (25.20%) at equivalent (70B) or even more (176B) parameters (Δ ≤ 1.60%). (2) Second, while trillion-parameter commercial general-purpose LLMs (GPT-4o: 30.8%; Deepseek-R1-671B: 30.72%) surpass medically adapted LLMs, all remain statistically inferior to human physicians (32.5%), exposing fundamental limitations in existing LLMs for full-process clinical diagnosis. (3) Third, DxDirector-7B achieves accuracy—a 5.9% absolute improvement over physicians and 7.6% over GPT-4o—despite using merely 4%–10% parameters of medically adapted LLMs (7B vs. 70B, 176B) and nearly 1% of commercial general-purpose LLMs. This demonstrates the effectiveness and efficiency of our training method. The results redefine optimization strategies for medical LLMs, proving that lightweight models with powerful deep thinking ability can master complex full-process clinical reasoning, rather than brute-force scaling or narrow pretraining on large scale medical data.

The ClinicalBench—spanning 1500 real-world cases across 150 diseases—reveals the performance of LLMs in real-world full-process clinical diagnosis. Results in Fig. 3c show DxDirector-7B achieves the highest accuracy at 63.46%, outperforming the strongest commercial model (Deepseek-R1-671B: 47.28%) by 16.18% and medically adapted LLMs (Clinical Camel-70B: 31.43%; OpenbioLLM-70B: 32.86%; MedFound-176B: 35.76%) by 27.70–32.03%, despite using much fewer parameters. In addition to the conclusions consistent with NEJM and RareArena, the results on ClinicalBench suggest two important findings: first, compared to NEJM and RareArena, DxDirector-7B achieves the largest absolute improvement, which shows the significant advantages of DxDirector-7B in real-world diagnosis. Second, sole medical-domain training of LLMs cannot be efficiently translated to full-process diagnosis in clinical practice, as OpenbioLLM-70B (32.86%) and MedFound-176B (35.76%) shows worse performance than general LLM Llama-3-70B (38.86%).

### Quantitative analysis of human physicians’ workload

This section analyzes the workload needed by human physicians when LLMs drive the full-process clinical diagnostic. In our constructed AI-driven full-process clinical diagnosis setting, to maximize the potential of LLMs and reduce the workload of human physicians as much as possible, human physicians only need to follow the instructions of LLMs to complete clinical operations that LLMs cannot achieve, such as observing symptoms, physical examinations, laboratory tests, etc. An ideal LLM should be capable of precisely identifying the essential clinical tasks that truly require human intervention, adhering to the principle of minimizing physician involvement while ensuring diagnostic accuracy. To quantitatively assess this, we introduce two key metrics: (1) the total number of clinical operations that the LLM requests physicians to perform throughout the diagnostic process (where fewer requests indicate greater efficiency), and (2) the proportion of operations that are truly useful for making an accurate diagnosis out of all requested operations. (the higher the better). The implementation method for the above judgments is described in section “Evaluation metrics”. The specific clinical operations can be found in word cloud analysis of Supplementary Figs. 1–10.

**Fig. 4:**
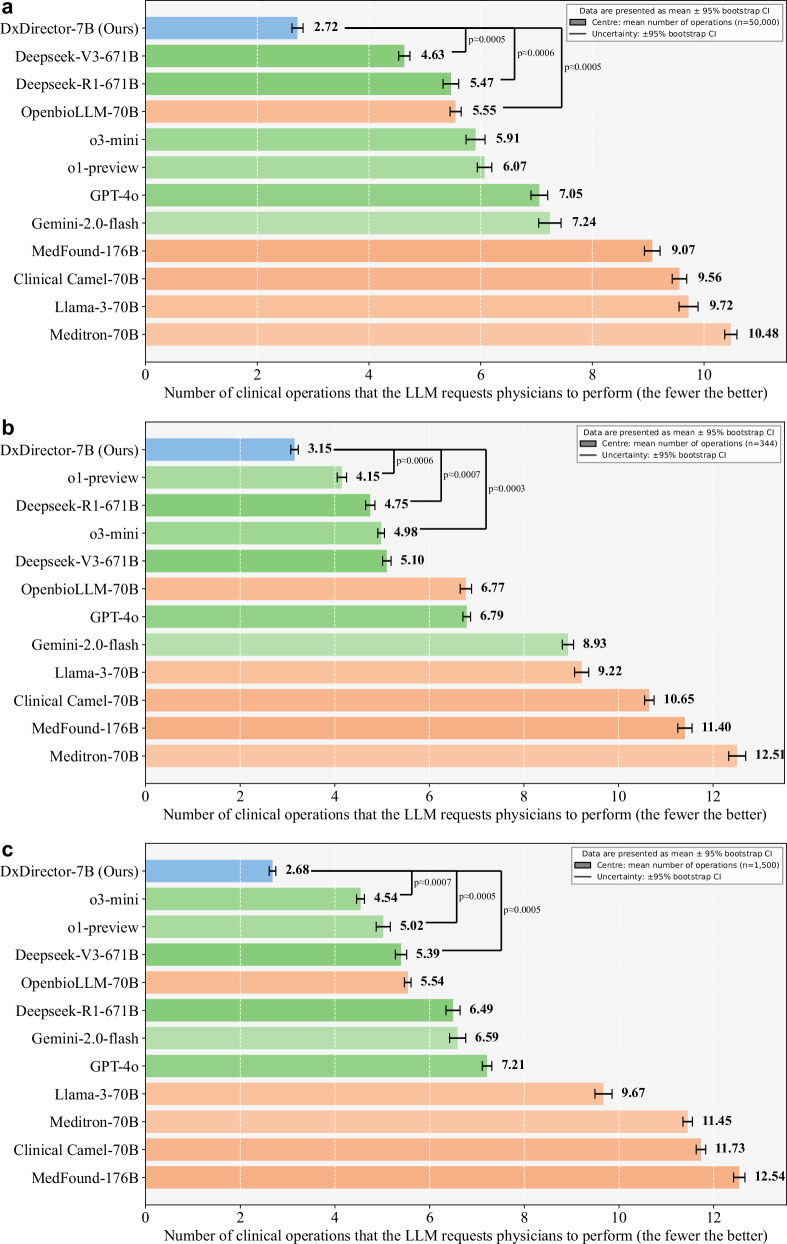
Number of clinical operations that LLMs request physicians to perform in the entire diagnosis process (the fewer the better). **a** Results on dataset with Rare Disease Cases (RareArena). **b** Results on dataset with Complex Cases (New England Journal of Medicine Clinicopathologic Cases). **c** Results on dataset with Real-world Cases (ClinicalBench). Error bars reflect 95% confidence intervals determined by non-parametric bootstrap procedure with 1000 samples on RareArena and ClinicalBench, and 200 samples on NEJM Cases. We perform statistical significance tests utilizing two-side Mann-Whitney *U* test between DxDirector-7B and the top-3 baselines, with *p* value levels annotated on the bars. Source data are available in the Source Data file (Figure-4-a.xlsx, Figure-4-b.xlsx, Figure-4-c.xlsx).

For the first metric, the average statistical results over three datasets are shown in Fig. 4a–c. Specifically, DxDirector-7B effectively complete the entire diagnostic process with an average of approximately 3 clinical operations across diverse diagnostic scenarios, including rare diseases (RareArena), complex cases (NEJM), and real-world clinical contexts (ClinicalBench). This efficiency notably surpass that of all baseline LLMs. By comparison, general-purpose commercial LLMs typically necessitate between 4 and 8 operations, while open-source medically adapted LLMs exhibit the poorest performance, often requiring nearly 10 clinical operations. Within baseline comparisons, commercial general-purpose LLMs such as o3-mini and o1-preview, benefiting from robust reasoning capabilities, consistently require fewer operations than other LLMs, including GPT-4o. Enhanced reasoning capacity allows these LLMs to effectively leverage available clinical data to make accurate clinical decision, thus minimizing additional operational demands.

**Fig. 5:**
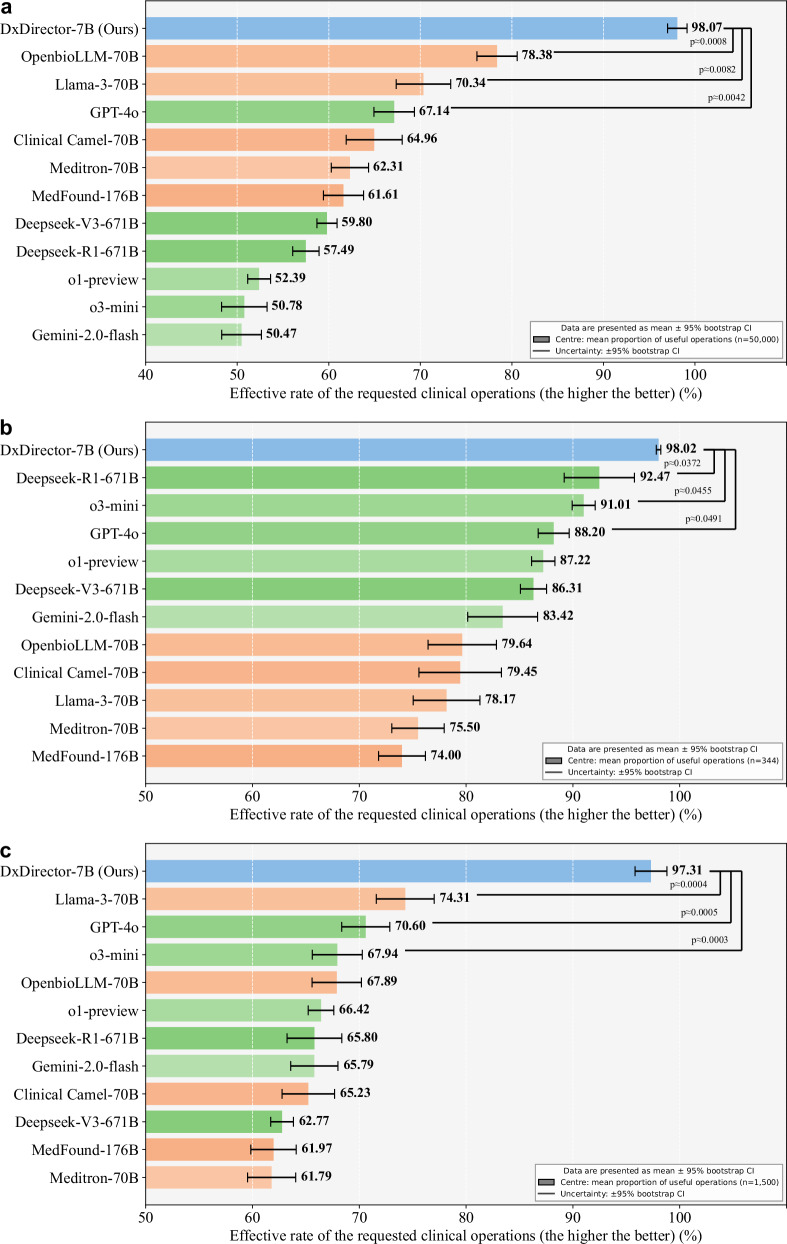
Proportion of operations that are truly useful for making a diagnosis out of all requested operations. (the higher the better). **a** Results on dataset with Rare Disease Cases (RareArena). **b** Results on dataset with Complex Cases (New England Journal of Medicine Clinicopathologic Cases). **c** Results on dataset with Real-world Cases (ClinicalBench). Error bars reflect 95% confidence intervals determined by non-parametric bootstrap procedure with 1000 samples on RareArena and ClinicalBench, and 200 samples on NEJM Cases. We perform statistical significance tests utilizing Mann-Whitney *U* test between DxDirector-7B and the top-3 baselines, with *p* value levels annotated on the bars. Source data are available in the Source Data file (Figure-5-a.xlsx, Figure-5-b.xlsx, Figure-5-c.xlsx).

For the second metric, we determine whether an operation genuinely contributes to diagnosis by assessing whether it appears in the case report provided by medical specialists. This metric serves as an indicator of the LLMs’ proficiency in discerning essential operations necessary for accurate diagnosis while avoiding any redundant operations, with higher values reflecting greater efficiency in engaging human physician assistance. Experimental results across three datasets are presented in Fig. 5a–c. DxDirector-7B demonstrates consistently superior performance, achieving efficiency ranging from 97% to 98% across all datasets, significantly surpassing the baselines. The performance of the baselines varies. In the diagnosis of complex cases (NEJM), general-purpose LLMs consistently outperform medically adapted LLMs, whereas in the diagnosis of rare diseases (RareArena), medically adapted LLMs surpass general-purpose LLMs. This indicates that the improvement on efficiency in seeking human physicians’ assistance is jointly driven by reasoning capabilities and the retention of specialized medical knowledge. General-purpose commercial LLMs excel in the former, while medically adapted LLMs excel in the latter.

### Department-level fine-grained evaluations

**Fig. 6:**
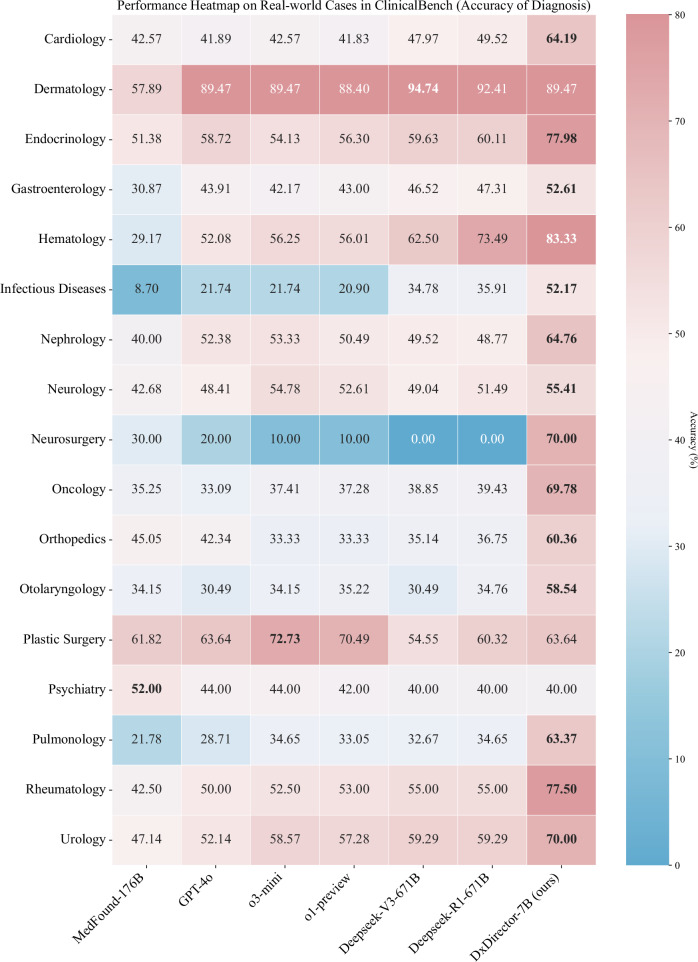
A comparative heatmap analysis of diagnostic accuracy. This figure shows the results of DxDirector-7B vs. state-of-the-art medically adapted and commercial general-purpose large language models (LLMs) across 17 clinical departments consisting of 1500 samples in ClinicalBench that is collected from real world. Source data are available in the Source Data file (Figure-6.xlsx).

In this part, we categorize the data from ClinicalBench and RareArena by clinical department and assess the diagnostic accuracy within each category, providing a more granular evaluation of LLMs. The heat map in Fig. 6 illustrates the diagnostic accuracy across 17 clinical departments comprising 1500 real-world cases within ClinicalBench. DxDirector-7B achieves greater performance on 14 out of 17 departments. ClinicalBench can reflect the true clinical distribution encountered in routine practice. DxDirector-7B significantly outperforms all baseline LLMs, with substantial margins observed particularly in Neurosurgery (Δ = 40.0%), Oncology (Δ = 30.93%) and Pulmonology (Δ = 28.72%). Diagnoses within these departments typically necessitate comprehensive integration of multiple diagnostic tests. It is a challenging scenario for existing state-of-the-art LLMs, which struggle to actively pursue and integrate necessary diagnostic information starting from only vague patient chief complaints. DxDirector-7B cannot achieve greater performance on Dermatology, Plastic Surgery and Psychiatry. It is mainly because that the clinical diagnosis of these three departments depends on frequent real contact, observation, and interactions between human physicians and patients, which does not play to the advantages of DxDirector-7B.

**Fig. 7:**
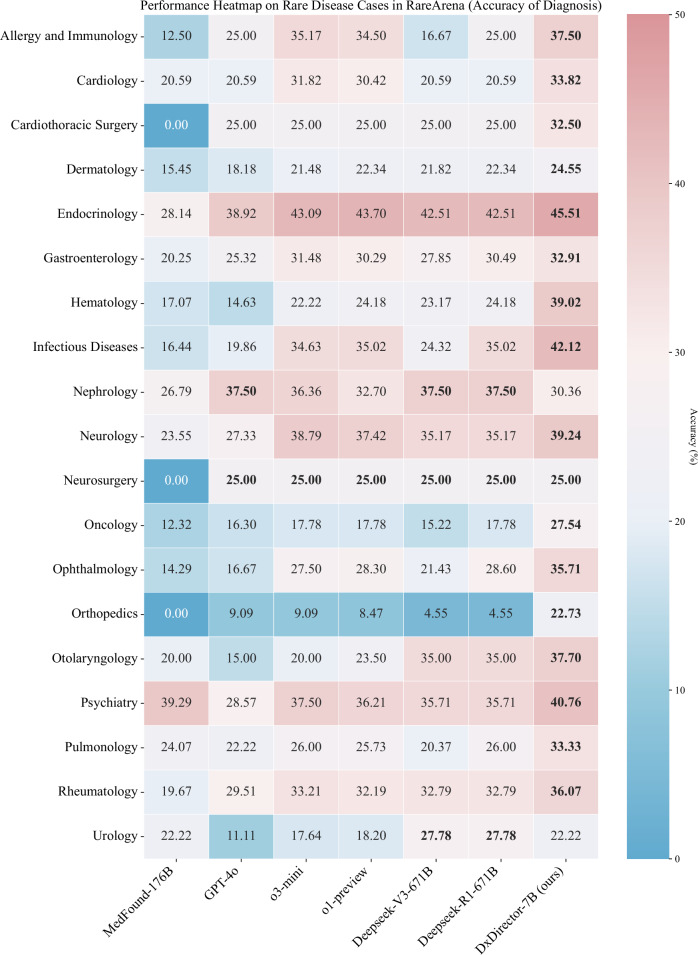
A comparative heatmap analysis of diagnostic accuracy. This figure shows the results of DxDirector-7B vs. state-of-the-art medically adapted and commercial general-purpose large language models (LLMs) across 19 clinical departments in rare disease cases on 22,901 samples in RareArena. Source data are available in the Source Data file (Figure-7.xlsx).

The heat map in Fig. 7 illustrates the diagnostic accuracy across 19 clinical departments for rare disease diagnosis, based on 22,901 samples from RareArena. Notably, DxDirector-7B, outperforms all baselines in 16 out of 19 departments. In particular, DxDirector-7B demonstrates substantial improvements in diagnostic accuracy for rare diseases in Hematology (Δ = 14.84%), Orthopedics (Δ = 13.64%), Oncology (Δ = 9.76%), Pulmonology (Δ = 7.33%), and Infectious Diseases (Δ = 7.1%). The diagnosis of rare diseases in these departments emphasizes that LLMs can accurately plan and integrate diagnostic tests at multiple stages, integrate travel and contact history with laboratory results, and perform image-test collaborative reasoning. These capabilities are the core of LLMs in driving full-process clinical diagnosis, demonstrating the superiority of DxDirector-7B in this regard. DxDirector-7B does not surpass Deepseek-v3-671B on Urology (Δ = −5.56%) and Nephrology (Δ = −7.14%). Given the overlap in medical knowledge related to the urinary system and kidney function between these two departments, this limitation suggests that DxDirector-7B may have gaps in its medical knowledge concerning rare diseases in these domains. This analysis shows the strengths and limitations of DxDirector-7B compared to state-of-the-art LLMs in clinical diagnosis across various departments.

### Evaluations on real-world clinical diagnosis

In this section, we introduce medical specialists to participate in the evaluation of LLMs in real-world clinical diagnosis scenarios. The real clinical diagnostic scenario is set within an officially certified Grade 3A hospital in China. The involved patients are inpatients presenting with more complex conditions than typical outpatients. Consequently, LLMs must engage in intricate reasoning to gather comprehensive clinical information effectively. To safeguard patients from potential harm, the evaluation environment is structured as follows: patient behaviors and medical specialist operations during clinical diagnosis are fully documented from actual inpatient records. Subsequently, two GPT-4o-based agents replicate precisely the recorded behaviors of patients and specialists throughout the diagnostic process. In evaluation, LLMs interact with these agents to drive the full-process diagnosis, initiating solely from the patient’s vague chief complaint. Within this controlled environment, LLMs do not directly interact with real patients, and their diagnostic outputs undergo rigorous review by medical specialists, thereby effectively mitigating ethical risks and potential harm.

This evaluation is performed on 160 cases across 9 different clinical departments including Gastroenterology, Nephrology, Dermatology, Cardiovascular Medicine, Infectious Diseases, Endocrinology, Pulmonology, General Surgery, and Pain Management. We compare DxDirector-7B with the most powerful commercial LLMs including GPT-4o, o1-preview, o3-mini, Deepseek-V3-671B and Deepseek-R1-671B, which possess tens of times more parameters than DxDirector-7B. Medical specialists from each department participate in evaluating the diagnostic contents produced by these LLMs. This evaluation is conducted from two aspects: (1) scoring the diagnostic content generated by LLMs (on a scale from 0 to 10), and (2) assessing whether the diagnoses generated by LLMs could fully replace those made by medical specialists. To ensure objective assessments and mitigate potential biases in human specialists scoring, a double-blind adjudication approach is implemented. In this approach, both human specialists and LLMs independently diagnose the same patient cases without exposure to each other’s diagnostic outputs. Additionally, a third-party evaluation agent, utilizing both GPT-4o and Deepseek-V3, assigns scores based on the alignment between LLM-generated diagnoses and those provided by medical specialists. The final score is calculated as the average of the scores given by two independently trained LLMs from different organizations (GPT-4o and DeepSeek-V3) to mitigate single-model biases and reduce variance in the judge. Averaging their scores yields a more stable, conservative estimate of alignment to the physician reference. The assessment of whether the diagnoses generated by LLMs could replace those made by specialists also follows the same pattern by observing the decisions of the GPT-4o-based third party agent mentioned above (can or cannot).

**Fig. 8:**
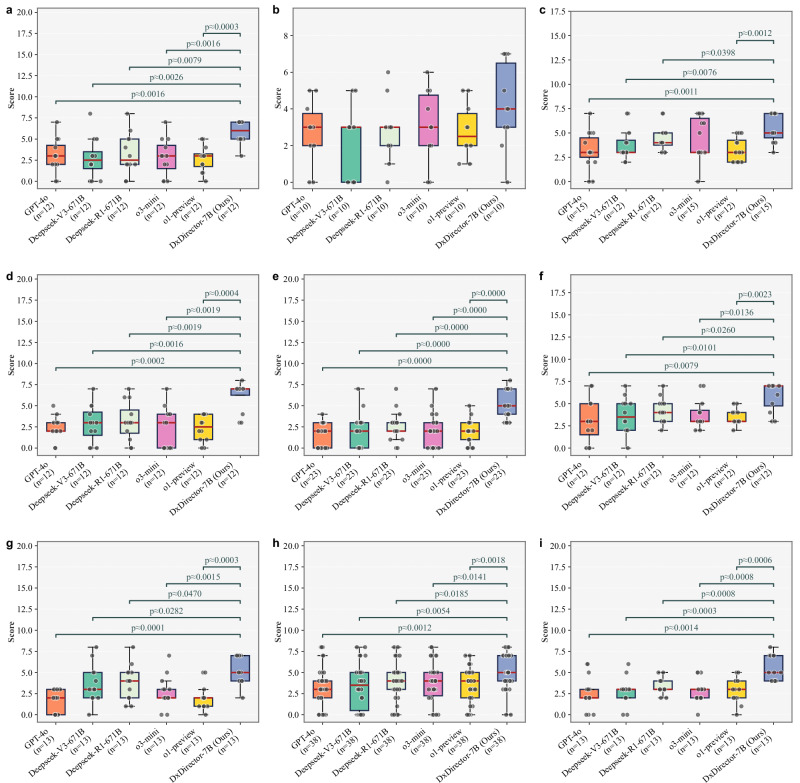
A comparison of the capabilities of different large language models (LLMs) in 9 departments in real-world clinical diagnosis. **a** Cardiovascular Medicine. **b** Dermatology. **c** Endocrinology. **d** Gastroenterology. **e** General Surgery. **f** Infectious Diseases. **g** Nephrology. **h** Pain Management. **i** Pulmonology. The evaluation is conducted by double-blinded adjudication between LLMs and specialists in the corresponding departments, with scores ranging from 0 to 10. The sample size (*n*) for each model represents the number of independent clinical cases evaluated and is indicated on the x-axis. Each data point represents a distinct clinical scenario (biological replicate); no technical replicates were pooled. Box plots illustrate the distribution of scores for each model. Box Plot Definitions: The central red line represents the median (50th percentile). The lower and upper boundaries of the box indicate the 25th and 75th percentiles, respectively (defining the Interquartile Range, IQR). The whiskers extend to the minimum and maximum values that fall within 1.5 × IQR from the box edges. Data points outside this range are plotted individually as outliers. We perform statistical significance tests utilizing the two-side Mann-Whitney *U* test between DxDirector-7B and the baselines, with *p* value levels annotated on the figures. Source data are available in the Source Data file (Figure-8.xlsx).

The results of the first aspect are shown in Fig. 8. Overall, DxDirector-7B achieves the highest alignment with medical specialists in all 9 clinical departments. The significant lead is evident in Cardiovascular, Pulmonology, and Gastroenterology. In these departments, patients’ chief complaints are far from sufficient to determine the final diagnosis, requiring additional diagnostic test results such as CT scans, angiography, blood tests, and more. These necessitate LLMs to actively acquire the complete clinical information by step-by-step reasoning to finish the entire diagnostic process, posing a substantial challenge for existing LLMs and DxDirector-7B can effectively address.

**Fig. 9:**
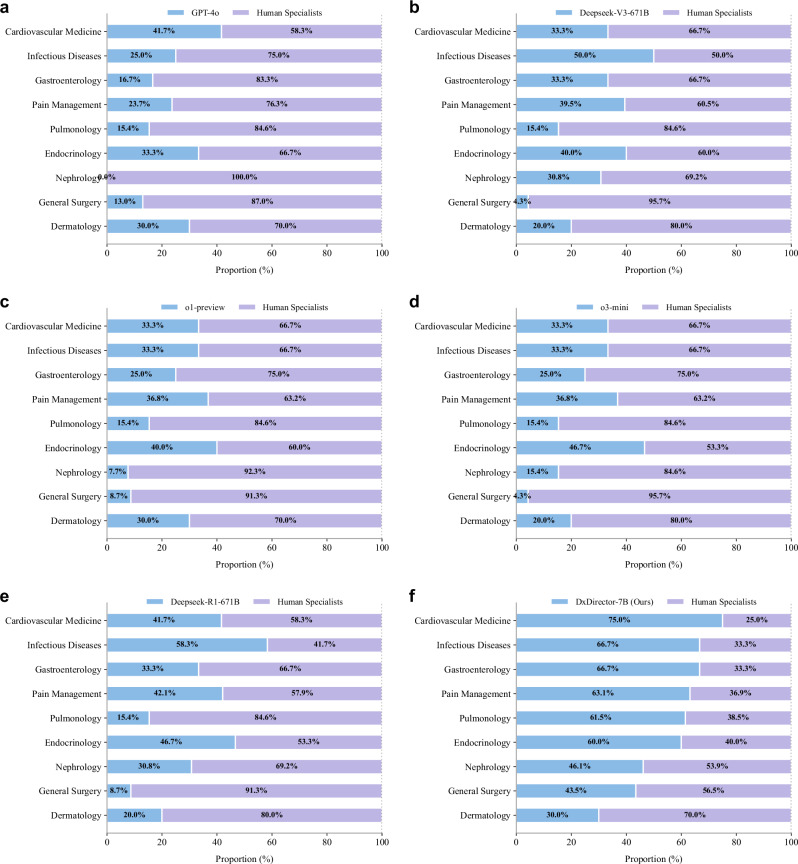
The proportion of the diagnoses generated by large language models (LLMs) can completely replace those of medical specialists in each department. **a** GPT-4o vs. Specialists. **b** Deepseek-V3-671B vs. Specialists. **c** o1-preview vs. Specialists. **d** o3-mini vs. Specialists. **e** Deepseek-R1-671B vs. Specialists. **f** DxDirector-7B (Ours) vs. Specialists. The assessment is conducted by double-blinded adjudication between LLMs and specialists in the corresponding department. Source data are available in the Source Data file (Figure-9.xlsx).

The results of the second aspect are shown in Fig. 9. In this evaluation, the GPT-4o-based third-party agent assesses whether the diagnoses generated by DxDirector-7B could replace those made by medical specialists. The bar of Fig. 9 indicates the proportion of samples that LLMs can replace specialist physicians to the total number of samples. All baseline LLMs fail to outperform specialist physicians in all departments. On the contrary, the diagnostic contents generated by DxDirector-7B in Cardiovascular Medicine achieves a 75.0% replacement rate to specialist physicians. In Infectious Diseases, Gastroenterology, Pain Management, Pulmonology and Endocrinology, DxDirector-7B achieves 60%–66.7% replacement rate to specialist physicians. These departments reply on the comprehensive analysis and reasoning of clinical testing information. For departments such as Dermatology and General Surgery where physical operations such as contact, observation, and real-time response are dominant, DxDirector-7B cannot achieve replacement rates of more than 50%, because in real-world clinical diagnosis, these departments strongly rely on frequent real interactions with human physicians and patients.

We introduce professional human physicians to manually review the scoring results of the AI agents in the above evaluation. Specifically, we randomly sample 10% of the test cases from all baseline LLMs and our DxDirector for three independent physicians to review. We compare the rank ordering of models under human scores vs. AI agent scores using rank-based statistics (Kendall’s *τ* = 0.83), and report inter-rater reliability between clinicians (Intraclass Correlation Coefficient (ICC) = 0.79). We observe consistent model rankings between human review and the agents.

### Evaluations on US medical license exam consisting of various clinical tasks

In this section, we assess the performance of LLMs using the United States Medical Licensing Examination (USMLE) dataset, which comprises 1273 publicly available cases covering various clinical tasks, such as diagnosis, differential diagnosis, prevention, etiological analysis, etc. To better replicate realistic clinical scenarios and elevate the complexity, we convert the original multiple-choice format of the USMLE questions into open-ended questions. This transformation demands more sophisticated reasoning and clinical inference from the LLMs. In this transformed dataset, LLMs are required to address questions across various tasks under full-process diagnosis setting. Here, only the patient’s chief complaint is initially provided, and the LLMs must actively infer and gather more detailed clinical information through more reasoning.

#### Overall accuracy

**Fig. 10:**
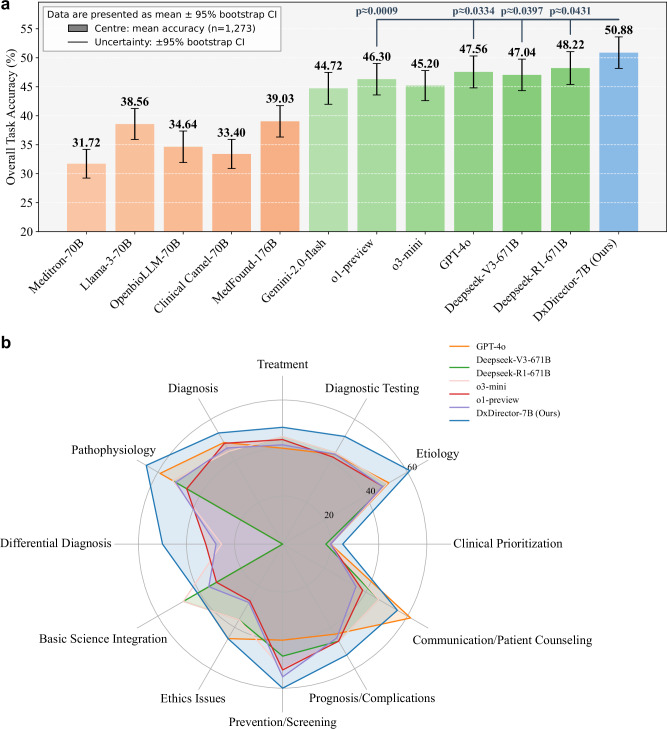
Performance on US Medical License Exam (USMLE)^22^. **a** Accuracy of answering questions about various clinical tasks on US Medical License Exam (USMLE) in full-process diagnosis setting. Bars are annotated with the accuracy of each LLM. Error bars reflect 95% confidence intervals determined by non-parametric bootstrap procedure with 1,000 samples. We perform statistical significance tests utilizing the two-side McNemar test between DxDirector-7B and the top-4 baseline, with *p* value levels annotated on the bars. **b** The visual comparison among various large language models (LLMs) on 12 clinical tasks in USMLE. Source data are available in the Source Data file (Figure-10-a.xlsx, Figure-10-b.xlsx).

The overall performance of various LLMs on the US Medical Licensing Examination (USMLE) is illustrated in Fig. 10a. DxDirector-7B achieves the highest accuracy (50.88%), underscoring its superior capabilities not only in making diagnosis but also across a broader array of clinical tasks, thereby highlighting its versatility for practical healthcare applications. Notably, DxDirector-7B outperforms medically adapted LLMs such as MedFound-176B, attaining a significant absolute improvement of 11.85% despite having only approximately one-tenth of the parameter size (7B compared to 70B and 176B). Furthermore, medically adapted LLMs with similar parameter sizes (OpenbioLLM-70B, Clinical Camel-70B, and Meditron-70B) demonstrate inferior performance (by Δ = −6.84% to −3.92%) compared to the general LLM Llama-3-70B. This observation suggests that existing medical adaptation methods, while effective at enhancing diagnostic accuracy, may inadvertently compromise performance on other critical tasks in full-process clinical diagnosis setting. Collectively, these comparisons emphasize the efficacy and generalizability of our training method employed in developing DxDirector-7B.

#### Specific accuracy on twelve clinical tasks

Figure 10b provides a detailed comparison of the performance of various LLMs across 12 clinical tasks within the USMLE dataset, offering granular insight into their comprehensive clinical capabilities in full-process diagnosis. DxDirector-7B outperforms all powerful commercial LLMs on 10 out of 12 tasks. Specifically, DxDirector-7B achieves large improvement over all baselines in differential diagnosis (Δ = 18.00%) and etiology (Δ = 10.13%). Both of these two tasks require LLMs to obtain as detailed and accurate clinical test information as possible to rule out potential disease options and determine the etiology.

### Accountability in misdiagnosis

In this section, we evaluate the accountability of DxDirector-7B in cases of misdiagnosis. Unlike existing LLMs, which present the entire diagnostic reasoning process interwoven with multiple human physician operations without clear distinction, DxDirector-7B explicitly structures the diagnostic process (Supplementary Fig. 1). Each diagnostic step is clearly itemized, distinguishing the content generated by the LLM from that provided by human physicians, and each LLM-generated step is explicitly attached by authoritative medical literature. This structured approach enables precise identification of specific erroneous steps during a misdiagnosis and clarifies responsibility between the LLM and physicians.

To evaluate the accountability capability of DxDirector-7B in cases of misdiagnosis, we simulate diagnostic errors by introducing perturbations to randomly selected steps within the diagnostic process to make LLM generate the incorrect diagnosis. The specific perturbation method is employing Deepseek-V3 to rewrite the selected steps to generate new content that is factually inconsistent with the original content. These perturbations can impact either the LLM-generated content, the involvement of human physicians, or both. In this scenario, a GPT-4o-based agent is employed to assess whether it can correctly identify the source of misdiagnosis—whether attributed to human physicians, LLMs, or both—thereby evaluating the effectiveness of the accountability mechanism. This evaluation constitutes a three-class classification task, utilizing precision and recall as primary metrics. The evaluation dataset comprises 1500 sampled cases that all LLMs can generate the correct diagnosis from RareArena and ClinicalBench. Results, see Supplementary Fig. 1, indicate that DxDirector-7B’s accountability mechanism achieves the highest precision and recall across all categories compared to baseline LLMs. Notably, baseline LLMs typically attribute errors disproportionately to human physicians over LLMs, reflected by higher recall but lower precision for physician accountability tha LLM (recall: 78.23–80.91% vs. 49.35–53.42%; precision: 60.42–62.15% vs. 70.40–71.95%), which means that the diagnostic content generated by baselines makes physicians over-accountable compared to LLMs. In contrast, DxDirector-7B maintains comparable and high precision and recall for both LLM and physician accountability. This means that providing clear and fine-grained content attached by authoritative medical literature is of great significance for achieving an accurate medical accountability mechanism.

## Discussion

### Reverse the physician–AI relationship

Most current AI systems still function primarily as tools for physicians in the specific parts of clinicl diagnosis. This AI-assisted workflow limits AI’s ability to fully reduce physician workload and improve diagnostic efficiency. In this paper, we propose a new paradigm that reverses the physician–AI relationship: we train an LLM to serve as the director of the full clinical diagnostic process, while physicians act as assistants who provide input only when necessary, following the principle of minimizing physician involvement. This paradigm supports effective AI deployment in real-world, full-process clinical workflows, reduces physician workload to the greatest extent possible, and enables an efficient, accurate, and scalable diagnostic solution.

### Superior diagnostic accuracy in a full-process clinical diagnosis setting

Building on this design, we propose DxDirector-7B, an LLM with strong deep-thinking capability that can drive full-process clinical diagnosis with minimal human effort. By doing so, it reduces physician workload and, as much as possible, lowers the need for specialized expertise in practical clinical tasks. We evaluate DxDirector-7B in a full-process setting across four publicly available, authoritative datasets (including rare and complex cases) and in a real-world clinical diagnostic scenario in a top-tier hospital in China. In terms of diagnostic accuracy, results in Figs. 3, 8, and 10 show that DxDirector-7B substantially outperforms medically adapted LLMs with dozens of times more parameters (e.g., MedFound-176B) as well as commercial general-purpose LLMs with nearly 100 times more parameters (e.g., GPT-4o, o1-preview, o3-mini, and Deepseek-R1-671B). These results indicate that DxDirector-7B is not only accurate but also computationally economical, representing a solid step toward low-cost and effective deployment of LLMs in practical clinical diagnostics.

### Significantly reducing physician workload

The workload that physicians must bear during full-process diagnosis is a crucial metric for evaluating the extent to which AI empowers clinical diagnosis. Results in Figs. 4 and 5 show that, across the entire diagnostic process, DxDirector-7B requires the least physician effort, achieves the highest physician efficiency, and delivers the most accurate diagnoses compared with existing state-of-the-art LLMs. These findings suggest that DxDirector-7B provides an efficient, accurate, and scalable diagnostic solution.

### Comprehensive department-level understanding

To obtain a more comprehensive view of DxDirector-7B’s strengths and weaknesses across clinical departments, we compare DxDirector-7B with baseline models at the departmental level in Fig. 6 (17 departments, real-world cases) and Fig. 7 (19 departments, rare diseases). The results show that DxDirector-7B achieves substantially higher diagnostic accuracy than all strong baselines in most departments, particularly in complex departments that require many diagnostic tests (e.g., oncology). This suggests that DxDirector-7B is better able to select and sequence appropriate diagnostic tests during full-process diagnosis, iteratively enriching clinical information to support accurate conclusions. We attribute this advantage to DxDirector-7B’s ability to perform continuous deep thinking to make better stepwise decisions, highlighting the importance of developing LLMs with stronger deep-thinking capabilities for clinical diagnosis.

### Potential to substitute for medical specialists in real-world diagnosis

Experiments on real-world cases across nine clinical departments in a top-tier hospital in China further demonstrate the advantages of DxDirector-7B for practical clinical diagnostic applications (Fig. 8). Evaluations involving medical specialists show that DxDirector-7B’s diagnoses could substitute for specialist judgment in 60–75% of cases in several departments (Fig. 9). These results outperform all state-of-the-art LLM baselines and indicate that DxDirector-7B has strong potential to serve as a viable substitute for medical specialists in real-world diagnosis.

### Superior performance across diverse clinical tasks

Diagnosis is not the only task in clinical practice. We further evaluate LLMs on 12 clinical tasks (e.g., differential diagnosis, treatment, and etiology) at the US Medical Licensing Examination level. DxDirector-7B outperforms all strong commercial LLMs on 10 of the 12 tasks, with particularly large improvements on tasks that require LLMs to obtain clinical test information as completely and accurately as possible, such as differential diagnosis and etiology. Overall, DxDirector-7B is expected to serve as an all-around director in clinical workflows, completing diverse clinical tasks while minimizing the need for assistance from human physicians.

### Accurate accountability mechanism

Establishing a clear and accurate accountability mechanism between physicians and AI for misdiagnosis is essential when both parties work closely together. DxDirector-7B produces a structured diagnostic record (Supplementary Fig. 1) in which each step is itemized; LLM-generated content is clearly distinguished from physician-provided input; and each LLM-generated step is supported by authoritative medical literature. This structured output enables clear identification of specific erroneous medical steps and supports attribution of responsibility between physicians and LLMs in cases of misdiagnosis. Evaluation on a large misdiagnosis dataset shows that, compared with all strong baseline LLMs, DxDirector-7B provides a more accurate and fair mechanism for accountability in misdiagnosis.

### Clinical relevance and safety

While benchmark accuracy provides an important quantitative measure of diagnostic capability, true clinical applicability requires more than correctness of the final diagnosis. In real-world practice, physicians prioritize patient safety, risk stratification, and timely therapeutic decisions. First, DxDirector-7B incorporates an escalation mechanism: when it encounters patterns indicative of high-risk or emergent conditions (e.g., severe hemodynamic instability, critical lab abnormalities, or suspected malignancy), the system is designed to flag the case and immediately defer decision-making to physicians. This ensures that urgent cases are not delayed by automated reasoning and that clinical oversight is preserved at critical junctures. Second, the model’s structured outputs include stepwise logic, supporting medical literature, and explicit attribution of which steps involved physician input. This creates a transparent accountability framework that enhances traceability in the event of misdiagnosis and reinforces physician responsibility for final decisions. Finally, by reducing physician workload on repetitive and low-risk reasoning tasks while retaining human oversight for high-stakes decisions, DxDirector-7B helps balance efficiency with safety. This paradigm aligns with clinical norms by safeguarding professional autonomy and patient trust, while demonstrating that AI can contribute meaningfully to healthcare delivery without undermining the ethical and professional foundations of medical practice.

### Value and impact

These results show that DxDirector-7B reshapes collaboration between AI and human physicians, signaling an era in which AI—traditionally regarded as a physician’s tool—can assume a director role by autonomously steering the full diagnostic process while minimizing physician involvement. This paradigm shift is designed to substantially reduce physician workload, improve efficiency, and increase diagnostic accuracy. By reducing reliance on physician time and specialized expertise, DxDirector-7B lowers barriers to high-quality diagnosis. By delivering a low-cost, efficient, and accurate clinical solution, DxDirector-7B may have substantial impact, particularly in medically underserved and resource-limited regions, and it appears promising across multiple departments and tasks. Moreover, its substantially lower parameter count—and correspondingly lower training and inference costs compared with existing state-of-the-art LLMs—makes DxDirector-7B more feasible to deploy across a broad range of medical institutions.

### Limitations and future work

DxDirector-7B performs worse than Deepseek-V3 and Deepseek-R1 on basic science integration. This is primarily due to the nearly 100 × gap in parameter count, which makes DxDirector-7B comparatively weaker at memorizing basic medical knowledge. In addition, GPT-4o, with stronger chat-oriented capabilities, performs better than DxDirector-7B in patient communication. Compared with other tasks, basic science integration and patient communication depend less on the ability to drive the entire diagnostic process; therefore, these capabilities were not specifically optimized in DxDirector-7B. Besides, inherent selection bias exists as the study was conducted at a single top-tier medical institution using inpatient records, which typically involve more complex conditions than outpatient cases, potentially limiting the generalizability of the results to primary care settings or hospitals with different resource levels. As for the future work, although DxDirector-7B has demonstrated strong performance, several directions warrant further study. For example, more refined and department-specific rules for physician participation could further improve efficiency. In addition, the assistants to DxDirector-7B may include not only human physicians but also other healthcare AI models. For instance, DxDirector-7B could call specialized pathology and imaging analysis models with visual understanding capabilities for Radiology^35–38^, Echocardiography^39^, Cell Slice Analysis^40^, Pathology^41^, and related domains. This could further reduce physician workload and improve diagnostic accuracy. At a high level, DxDirector-7B could serve as a director that establishes an efficient diagnostic framework and promotes effective collaboration among three key entities: physicians, patients, and specialized AI models. Continued exploration along these directions may transform the current healthcare paradigm: an LLM with exceptional reasoning capability could enable more automated, efficient mobilization and integration of medical resources, thereby improving both the efficiency and accuracy of healthcare delivery.

## Methods

**Statement about ethical regulations:** All data utilized in this research are exclusively for academic purposes, acquired ethically and legally, and have been reviewed and approved by the ethics committee of Peking University Third Hospital (IRB00006761-M20250173), ensuring adherence to ethical and legal standards. Data collection rigorously complies with principles of patient privacy protection. No hospital-related information is disclosed, and to further protect patient confidentiality, all PII, treatment locations, and other sensitive details are systematically identified and removed by the medical team. Written informed consent was obtained from the participants.

**Data Collection and Evaluation Setup:** Our real-world clinical diagnostic scenario is set within an officially certified Grade 3A hospital in China. The involved patients are inpatients presenting with more complex conditions than typical outpatients. Consequently, LLMs must engage in intricate reasoning to gather comprehensive clinical information effectively. To safeguard patients from potential harm, the evaluation environment is structured as follows: patient behaviors and medical specialist operations during clinical diagnosis are fully documented from actual inpatient records. Subsequently, two GPT-4o-based agents replicate precisely the recorded behaviors of patients and specialists throughout the diagnostic process. In evaluation, LLMs interact with these agents to drive the full-process diagnosis, initiating solely from the patient’s vague chief complaint. Within this controlled environment, LLMs do not directly interact with real patients, and their diagnostic outputs undergo rigorous review by medical specialists, thereby effectively mitigating ethical risks and potential harm. The evaluation is performed on 160 cases across 9 different clinical departments including Gastroenterology, Nephrology, Dermatology, Cardiovascular Medicine, Infectious Diseases, Endocrinology, Pulmonology, General Surgery, and Pain Management. To ensure objective assessments and mitigate potential biases in human specialists scoring, a double-blind adjudication approach is implemented. In this approach, both human specialists and LLMs independently diagnose the same patient cases without exposure to each other’s diagnostic outputs. Additionally, a third-party evaluation agent, utilizing both GPT-4o and Deepseek-V3, assigns scores ranging from 0 to 10 based on the alignment between LLM-generated diagnoses and those provided by medical specialists. The final score is calculated as the average of the scores given by GPT-4o and Deepseek-V3, thus ensuring robust and unbiased comparative assessment. The assessment of whether the diagnoses generated by LLMs could fully replace those made by medical specialists also follows the same pattern by observing the decisions of the third party agent (can or cannot).

In this section, we introduce the detailed training method of DxDirector-7B, which can be divided into three stages: (1) Continued pre-training on medical data; (2) Instruction-tuning for full-process clinical diagnosis; (3) Step-level strategy preference optimization. Then we introduce more details about our experiments.

### Continued pre-training on medical data

This stage enables the general LLM to acquire medical knowledge, which forms the foundation for its clinical diagnosis capabilities. Specifically, in this stage, we train open-source LLM (Llama-2-7B) on large-scale medical texts by cross-entropy loss function in the paradigm of next token prediction, being supervised by the learning signals from medical texts themselves. For example, given ${{\mathcal{C}}}$ is a dataset of texts, *C*_*i*_ = [*c*_1_, *c*_2_, *c*_3_, …*c*_*n*_] is one of the text sequence in ${{\mathcal{C}}}$ (${C}_{i}\in {{\mathcal{C}}}$), ${{\mathcal{M}}}({c}_{t}| {c}_{1},{c}_{2},\ldots {c}_{t-1};\theta )$ is the probability of *c*_*t*_ estimated by the LLM given prefix [*c*_1_, *c*_2_, …*c*_*t*−1_] and model parameters *θ*. the training objective in this continued pre-training stage is minimizing the negative log-likelihood over set ${{\mathcal{C}}}$ as:

1 $${\min }_{\theta }{\sum }_{{C}_{t}\in {{\mathcal{C}}}}{\sum }_{t=1}^{n}-\log {{\mathcal{M}}}({c}_{i}| {c}_{1},{c}_{2},\ldots {c}_{t-1};\theta ).$$

We collect publicly available medical data for this continued pre-training including 35K articles from clinical guidelines, 16.1M paper abstracts from PubMed and PubMed Central, 5M full papers from PubMed and PubMed Central^17^. Llama-2-7B is trained on these datasets to memorize the basic medical knowledge. Besides, we use experience replay^17^ to maintain the original general knowledge of LLama-2-7B by mixing 500K general domain data from Wikipedia, ArXiv, books, and StackExchange into the training datasets.

### Instruction-tuning for full-process clinical diagnosis

Instruction-tuning is the process of training the LLM that has been pre-trained to generate expected responses for user’s input instructions^42^. Full-process clinical diagnosis in the real world, especially for rare and complex cases, often involves multiple complex medical knowledge and multiple diagnostic test procedures. This section introduces our proposed instruction-tuning method that enables DxDirector-7B to drive full-process clinical diagnosis solely starting with ambiguous chief complaints, through a step-by-step reasoning and continuous deep thinking. The training at this stage aims to endow DxDirector-7B with four key capabilities:

1. **Progressive clinical information reasoning**: DxDirector-7B can gradually reason valuable clinical information starting with a patient’s vague complaint in a step-by-step manner, ultimately completing clinical tasks such as diagnosis, treatment plan design, etc. Each step may involve inference about medical knowledge, analyzing clinical phenomena, or designing diagnostic tests.
2. **Step-level deep thinking**: DxDirector-7B possesses deep thinking—that mimics human “slow thinking capabilities at the step level”. When determining the specific strategy for each step, it first generates a thinking process that analyzes the currently available information and the expected goal to define the optimal strategy for the current step.
3. **Human assistance when necessary**: In cases where the strategy requires clinical operations for diagnostic test—such as medical imaging, physical examinations, laboratory tests, etc, which computer program-based LLMs cannot complete—DxDirector-7B can request assistance from human physicians and continue reasoning after receiving the assistance.
4. **Autonomously generate final diagnosis**: DxDirector-7B can autonomously decide whether it can make am accurate final diagnosis based on the currently available information. The generated final diagnosis is a concise and clear summary of the step-by-step reasoning process, with each involved medical knowledge supported by authoritative medical literature.

We introduce the details about this stage in two parts: dataset construction and training.

#### Data construction

Constructing the suitable training dataset is the prerequisite. Our data construction can be divided into three steps: (1) raw data collection, (2) data transformation, and (3) deep thinking injection. The pipeline of our data construction is shown in Supplementary Fig. 2.

In raw data collection, our raw data is collected from MedQA^22^, a medical question-and-answer dataset enriched with extensive context, including detailed clinical information such as patient profiles, disease symptoms and histories, diagnostic test results, vital signs, etc. Questions in this dataset covers multiple clinical tasks such as diagnosis, differential diagnosis, designing treatment plan, screening, analyzing etiology, etc. This dataset contains 10,178 samples. After collecting the raw dataset, we perform data transformation on it to construct the data aligning with full-process diagnosis. We use GPT-4o API for fully automated data transformation, and introduce human medical experts perform sample evaluations of the transformed data to ensure the quality. For each data sample, data transformation consists of three steps as shown in “Data Transformation” part in Supplementary Fig. 2:

Firstly, we use GPT-4o to extract the patient’s detailed clinical information from the context, such as symptoms, medical history, all diagnostic test results, and vital signs, etc. In full-process clinical diagnosis, this information cannot be obtained at the beginning, but is gradually obtained during the complex consultation process.

Secondly, we use GPT-4o API to rewrite the original data into a patient-style chief complaint, which is a simple, vague, and non-professional description provided by the patient about their condition without any specific clinical information. This is the only information that LLMs can obtain from patients at the beginning of real-world full-process clinical diagnosis. Besides, we rephrase multiple-choice question in original data into open-ended question, which is closer to real-world clinical diagnosis. The rewritten chief complaint and open-ended question is synthesized into the instruction for the latter tuning.

Thirdly, we give the clinical information to GPT-4o and and design examples and complex prompts (i.e., in-context learning^43^) to instruct GPT-4o to convert the provided information to simulate the step-by-step reasoning process in full-process clinical diagnosis starting with the patient’s chief complaint. In this way, we construct the initial instruction-response pairs to fine-tune DxDirector-7B. As shown in the “Initial Response” of data transformation stage in Supplementary Fig. 2, each step consists of a question-answer pair in the paradigm of self-questioning and self-answering. The questions are divided into two types: one is inquiries or inference based on objective medical knowledge, such as the causes of a specific disease or determining the possible disease based on the patient’s specific symptoms, etc. These questions are marked as “<LLM>” and their answers can be finished by LLM itself. The other type of question involves inquiring about the clinical operations for diagnostic test or communication to patients. They are marked as “<Physician>” and their answer must be finished with the help of human physicians. When the reasoning is finished, the “[Final Content]” is generated, it is the summary of the reasoning process, with the number of reasoning steps marked at the corresponding positions. This enhances the credibility and error-correctability of the AI-generated diagnostic process. It is worth noting that GPT-4o cannot effectively perform full-process diagnosis. Therefore, to construct this data, we add the patient’s detailed clinical information to the context to GPT-4o. GPT-4o simulates the full-process diagnosis under the premise of already knowing all related information, which simplifies the task significantly. This approach enables us to construct a large amount of data that meets the requirements at a relatively low cost.

After transformation, we inject the deep thinking content for each step of “Initial Response”. As shown in deep thinking injection stage in Supplementary Fig. 2, we use o1-preview to generate detailed thinking content for each step of “Initial Response”. This thinking should fully consider the clinical information at the current step and combine it with the ultimate clinical goal to reason about the optimal strategy that should be taken at the current step, which simulates the human slow thinking process. Deep thinking makes the logical connection between each step in the whole process of clinical diagnosis closer. These contents often only appear in the minds of human physicians and are not written in electronic medical records. The explicit generation of these contents enables DxDirector-7B to have the slow thinking ability like human physicians. We do not generate deep thinking end-to-end during the data transformation stage because we find that doing so will result in deep thinking revealing currently unknown clinical information in advance.

The data instance in final instruction-response pairs for instruction-tuning for full-process clinical diagnosis is the instruction consisting of patient’s chief complaint and clinical question, and the response consisting of multi-step reasoning, deep thinking and final diagnosis.

During data construction, we randomly sample the transformed instruction-response pairs and provide them to human medical experts for evaluation to review whether this data aligns with real clinical diagnostic scenarios. We collect feedback from the medical experts and continuously refine our prompts to optimize the quality of the data. The detailed prompts in data construction can be found in Supplementary Figs. 11–20. Review criteria of human medical experts and the level of agreement between the experts and the generated dataset and be found in section 6 in Supplementary. Finally, we obtain 10,178 high-quality instruction-response pairs for training.

#### Training with decoupled reasoning and knowledge

We train DxDirector-7B to perform full-process clinical diagnosis on the constructed dataset above. As shown in instruction-response pair of Supplementary Fig. 2, given the instruction, DxDirector-7B is trained to generate the response consisting of numbered “[Deep Think]” and numbered “[Question]-[Answer]” pairs. “[Deep Think]” and “[Question]” emphasize reasoning capability while “[Answer]” emphasize medical knowledge recalling capability. We propose a decoupled training method based on loss-masking to enable DxDirector-7B to learn these two capabilities separately. It trains the two capabilities alternately in batches. When training reasoning ability, the loss function only computed over the content in “[Deep Think]” and “[Question]”, while the content in “[Answer]” and “[Final Content]” is masked. This allows the LLM to focus on reasoning the questions to be addressed at each step rather than recalling their answers. The cross-entropy loss function ${{{\mathcal{L}}}}_{1}$ for this can be computed as:

2 $${{{\mathcal{L}}}}_{1}={\sum }_{{R}_{i}\,\in {{\mathcal{Q}}}}-\log {{\mathcal{M}}}({R}_{i}| I,{R}_{1:i-1};\theta ),$$

in which ${{\mathcal{M}}}$ is the distribution of next token prediction of LLM, *I* and *R* are the instruction and response respectively of instruction-response pair in Supplementary Fig. 2. ${{\mathcal{Q}}}$ is set of tokens in “[Deep Think]” and “[Question]”, and *R*_1:*i*−1_ is the prefix for the token *R*_*i*_.

When training knowledge recalling capability, the loss function only includes the content in “[Answer]” and “[Final Content]”, while the content in “[Deep Think]” and “[Question]” is masked. Since some answers need the assistance from human physicians to obtain patient’s clinical information, the input data used to training this ability is accompanied by the extracted patient’s clinical information. The cross-entropy loss function ${{{\mathcal{L}}}}_{2}$ for this can be computed as:

3 $${{{\mathcal{L}}}}_{2}={\sum }_{{R}_{i} \,\notin \, {{\mathcal{Q}}}}-\log {{\mathcal{M}}}({R}_{i}| I,P,{R}_{1:i-1};\theta ),$$

in which *P* is the patient’s clinical information that used to simulated the assistance from human physicians in training. The details of the hyperparameters in training are provided in the section “Implementations”.

### Step-level strategy preference optimization

We call the “[Question]” to be solved in each step derived by deep thinking of DxDirector-7B as “strategy”. After instruction-tuning, DxDirector-7B has initially demonstrated the ability to drive full-process clinical diagnoses by generating the strategy step-by-step. However, instruction-tuning with token-level cross-entropy loss function cannot effectively make LLM learn how to make optimal strategy at each step, i.e., generate the most appropriate “[Question]”. The further optimization in this stage enables DxDirector-7B to implicitly compare multiple potential strategies in deep thinking at each step and select the optimal strategy. This ensures that each step in complex clinical reasoning is correct and efficient, so that the diagnosis can be completed accurately while relying on minimal human physician effort.

The training method we proposed in this stage is called Step-Level Strategy Preference Optimization, a reinforcement learning algorithm that assigns different rewards to different strategies at the same step and trains DxDirector-7B to generate the strategy with higher reward. To achieve this, first, we construct the training data that consists of multiple strategy labeled with different rewards for each step, and then design the specific method for preference optimization training.

#### Data construction

The overview of data construction for this stage can be found in Supplementary Fig. 3. At step *t*, we give instruction *I* and reasoning content from steps 1 to *t* − 1: {(*d*_1_, *q*_1_, *a*_1_), (*d*_1_, *q*_2_, *a*_2_), …, (*d*_*t*_, *q*_*t*−1_, *a*_*t*−1_)} as the input for ${{{\mathcal{M}}}}_{ft}$, in which *d*_*i*_, *q*_*i*_ and *a*_*i*_ are the deep thinking, question (i.e., strategy) and answer respectively at the *i* − *th* step, ${{{\mathcal{M}}}}_{ft}$ is the DxDirector-7B after instruction-tuning of the second stage. We change the random seed^44^ to make ${{{\mathcal{M}}}}_{ft}$ output *k* different responses at step *t*: $\{({d}_{t}^{1},{q}_{t}^{1},{a}_{t}^{1}),({d}_{t}^{2},{q}_{t}^{2},{a}_{t}^{2}),\ldots,({d}_{t}^{k},{q}_{t}^{k},{a}_{t}^{k})\}$ when faced with this input. In our implementation, we set *k* to 3 and more sampling-related hyperparameters can be found in section “Implementations”. For each response $({d}_{t}^{i},{q}_{t}^{i},{a}_{t}^{i})$, we assign a reward ${r}_{t}^{i}$ to it based on the correctness of the final answer generated by the reasoning path continued with this response and the degree of reliance on the human physicians. The correct answers are assigned higher rewards. For answers of the same correctness, the strategies that seek more assistance from human physicians will have a lower reward value than other strategies. The reward assigning strategy is as follows:

4 $${r}_{i}^{i}=\left\{\begin{array}{ll}\frac{10}{\gamma },& \, {{\rm{the}}} \; {{\rm{final}}} \; {{\rm{answer}}} \; {{\rm{is}}} \; {{\rm{correct}}} \hfill \\ 0,& \, {{\rm{the}}} \; {{\rm{final}}} \; {{\rm{answer}}} \; {{\rm{is}}} \; {{\rm{incorrect}}} \,,\end{array}\right.$$

in which *γ* is the number of requesting for assistance from human physicians. We assign a corresponding reward to each response and obtain the set ${{{\mathcal{S}}}}_{t}=\{({d}_{t}^{1},{q}_{t}^{1},{a}_{t}^{1},{r}_{t}^{1}),({d}_{t}^{2},{q}_{t}^{2},{a}_{t}^{2},{r}_{t}^{2}),\ldots,({d}_{t}^{k},{q}_{t}^{k},{a}_{t}^{k},{r}_{t}^{k})\}$ with rewards at step *t*. We generate all unique ordered pairs over ${{{\mathcal{S}}}}_{t}$, which can be described as:

5 $${{{\mathcal{P}}}}_{t}=\left\{\left(({d}_{t}^{m},{q}_{t}^{m},{a}_{t}^{m},{r}_{t}^{m}),({d}_{t}^{n},{q}_{t}^{n},{a}_{t}^{n},{r}_{t}^{n})\right)| {r}_{t}^{m} > {r}_{t}^{n},1\le m\le k,1\le n\le k\right\}.$$

In this way, we can get the data sample for strategy preference optimization training at step *t*, it consists of the input *X*_*t*_ = *I* + {(*d*_1_, *q*_1_, *a*_1_), (*d*_2_, *q*_2_, *a*_2_), …, (*d*_*t*−1_, *q*_*t*−1_, *a*_*t*−1_)} and a set of paired responses ${{{\mathcal{P}}}}_{t}$. Our strategy preference optimization training method optimizes DxDirector-7B learn to make the better choice in each pair $({d}_{t}^{m},{q}_{t}^{m},{a}_{t}^{m},{r}_{t}^{m}),({d}_{t}^{n},{q}_{t}^{n},{a}_{t}^{n},{r}_{t}^{n})$ of ${{{\mathcal{P}}}}_{t}$, maximizing the probability of generating the response with the higher reward while minimizing the probability of generating the response with the lower reward. Specific details about this will be introduced in section “Step-level preference optimization training”. The response with the highest reward in ${{{\mathcal{P}}}}_{t}$ will be added to the prefix for data construction in step *t* + 1. We use this strategy to iterate through each step of 2000 instruction-response pairs and finally get 23,608 data samples for training, this dataset is denoted as ${{\mathcal{D}}}$ ($({X}_{t},{{{\mathcal{P}}}}_{t})\in {{\mathcal{D}}}$).

#### Step-level preference optimization training

After data construction, we conduct step-level preference optimization training on ${{{\mathcal{M}}}}_{ft}$ to make it learn to implicitly compare multiple potential strategies in deep thinking at each step and select the optimal strategy with the principle of ensuring correctness while minimizing the workload of human physicians. The training objective is based on Direct Preference Optimization (DPO) loss function^45^:

6 $${{{\mathcal{L}}}}_{3}({\pi }_{\theta };{\pi }_{{{\rm{ref}}}})=-{{\mathbb{E}}}_{({X}_{t},{{{\mathcal{P}}}}_{t}) \sim {{\mathcal{D}}}}\frac{1}{| {{{\mathcal{P}}}}_{t}| }{\sum }_{\begin{array}{c}\left(({d}_{t}^{m},{r}_{t}^{m})\right.,\\ \left.({d}_{t}^{n},{r}_{t}^{n})\right)\in {{{\mathcal{P}}}}_{t}\end{array}}\left[\log \sigma \left(\beta \log \frac{{\pi }_{\theta }({d}_{t}^{m}| {X}_{t})}{{\pi }_{{{\rm{ref}}}}({d}_{t}^{m}| {X}_{t})}-\beta \log \frac{{\pi }_{\theta }({d}_{t}^{n}| {X}_{t})}{{\pi }_{{{\rm{ref}}}}({d}_{t}^{n}| {X}_{t})}\right)\right],$$

in which *π*_*θ*_(*b*∣*a*) is probability of the policy model generating sequence *b* given prefix *a*, *π*_ref_(*b*∣*a*) is probability of the reference model generating sequence *b* given prefix *a*, *β* is a hyperparameter usually between 0.1 to 0.5. *σ* is the sigmoid function. In this training objective, for each sample $({X}_{t},{{{\mathcal{P}}}}_{t})$ in the training set ${{\mathcal{D}}}$, we traverse each response pair $\left(({d}_{t}^{m},{r}_{t}^{m}),({d}_{t}^{n},{r}_{t}^{n})\right)$ in ${{{\mathcal{P}}}}_{t}$ and align DxDirector-7B’s deep thinking preference for the better strategy through the partial order relationship of rewards between $({d}_{t}^{m},{r}_{t}^{m})$ and $({d}_{t}^{n},{r}_{t}^{n})$, so as to enable DxDirector-7B to implicitly select the optimal strategy to generate among multiple potential strategies. For example, *X*_*t*_ is the input to ${{{\mathcal{M}}}}_{ft}$ at the *t* − *th* step, ${r}_{t}^{m} > {r}_{t}^{n}$, ${d}_{t}^{m}$ is the deep thinking for the strategy with higher reward and ${d}_{t}^{n}$ is the deep thinking for the strategy with lower reward. To optimize this loss function, DxDirector-7B should learn to maximize the probability of generating ${d}_{t}^{m}$ and minimize the probability of generating ${d}_{t}^{n}$. Since the strategy (“[Question]”) of each step is inferred from the content of deep thinking, optimization for deep thinking is more essential to enable DxDirector-7B to determine the optimal strategy for each step through more reasonable “slow thinking” like human. The details of the training are provided in the section “Implementations”.

### Training to search authoritative medical literature

After instruction-tuning and preference optimization, as “[Final Content]” shown in Supplementary Fig. 2, DxDirector-7B can summarize after multi-step reasoning and mark the referenced reasoning step numbers at the corresponding positions. These numbers not only point to the reasoning steps but also indicate references to authoritative medical literature. This innovative design enhances the verifiability and credibility of AI-generated diagnostic content at a fine-grained level. This section introduces our detailed training method for medical literature search model.

#### Training to search

##### Model Architecture

The base model for search is Gemma-2B^46^, a pre-trained language model with stacked 18 transformer layers. We convert Gemma-2B into a vector representation model that can represent the query and each paragraph in medical literature to a dense vector as follows:

Given an input paragraph *P* = {*x*_1_, *x*_2_, …, *x*_*T*_} with *T* tokens, the model outputs hidden states ${{{\bf{H}}}}^{l}\in {{\mathbb{R}}}^{T\times d}$ at each transformer layer *l* ∈ {1, 2, …, 18}. For text representation, we extract the last token’s hidden state from the final transformer layer:

7 $${{{\bf{h}}}}_{{{\rm{text}}}}={{{\bf{H}}}}^{L}[T,:]\in {{\mathbb{R}}}^{d},$$

in which *L* = 18 denotes the last transformer layer, *d* = 2048 is the hidden dimension of Gemma-2B, *T* is the sequence length of the input paragraph. This design leverages the autoregressive nature of Gemma-2B, where the final token’s representation naturally aggregates contextual information from all preceding tokens through the transformer’s self-attention mechanism. Given a query seeking medical knowledge, it represents the query and each paragraph in the corpus as vectors. The matching score between a query and a paragraph is determined by calculating the similarity between their vectors such as dot product. The paragraphs are then ranked in descending order based on their matching scores, and the top-k paragraphs are selected as the search results for the given query. To make the search model more stable and accurate in the vector representation of medical text, we train Gemma-2B on large-scale medical data in contrastive learning method, which will be introduced below.

##### Data collection and process

We collect a large amount of text data from the medical domain to train our model. The training dataset consists of 11*M* medical articles come from medical textbooks, publications, and case reports. For each article, we use its title as the query and its abstract as the paragraph matched with this query. In this way we construct a large-scale query-paragraph paired data for training the search model.

##### Training

We use in-batch contrastive learning to train our model, enabling it to accurately represent text as vectors and rank texts on vector similarity. Specifically, each training batch consists of *b* query-paragraph pairs. For the vector representation (**q**_*i*_) of a query in this batch, its positive sample is the paragraph paired with it (**p**_*i*_), while its negative samples are the *b* − 1 paragraphs paired with other queries within the same batch (**p**_*j*_, *j* ≠ *i*). For the query, in-batch contrastive learning aims to maximize the its vector similarity between the positive sample while minimizing its vector similarity between negative samples^47^. The loss function to achieve this can be described as:

8 $${{{\mathcal{L}}}}_{r}=-\frac{1}{b}{\sum }_{i=1}^{b}\log \frac{{e}^{{{{\bf{q}}}}_{i}^{\top }{{{\bf{p}}}}_{i}}}{{e}^{{{{\bf{q}}}}_{i}^{\top }{{{\bf{p}}}}_{i}}+{\sum }_{\begin{array}{c}j=1\\ j\ne i\end{array}}^{b}{e}^{{{{\bf{q}}}}_{i}^{\top }{{{\bf{p}}}}_{j}}}$$

#### Fine-grand medical literature search

##### Indexing corpus for search

We build a large-scale database consisting of 23.9M paragraphs from PubMed, 301.2K paragraphs from StatPearls, 125.8K paragraphs from medical textbooks as the corpus for medical literature search. We use our trained medical search model to represent each paragraph as a vector, and use IndexFlat (https://github.com/facebookresearch/faiss/blob/main/faiss/IndexFlat.h) method based on Faiss (https://github.com/facebookresearch/faiss) to index the vector for Approximate Nearest Neighbor (ANN)^48^ search.

##### Inference

We use each “[Question]” in multi-step reasoning of DxDirector-7B as the query to our search model. For each query, we use our trained search model to represent it to a vector. Then, we use ANN to search the indexed corpus for the paragraph vector that is closest to the query vector and obtain the paragraph corresponding to this paragraph vector as top-1 ranked paragraph in the search result. We mark this paragraph with corresponding serial number and attach it to the “[Final Content]”. In this way, we provide authoritative medical literature as a reference for each step in complex clinical diagnosis, which enables diagnostic readers to verify the diagnostic content more conveniently and judge the credibility of the diagnostic content in a fine-grained manner.

##### Evaluation

We evaluate the performance of this search model on the combined test set of ClinicalBench, RareArena and NEJM. The metric is Recall@k, one of the most commonly used evaluation metrics in the field of information retrieval. It is defined as the proportion of queries for which at least one accurate reference document appears in the top-k retrieved items. The evaluation results are: Recall@1 = 70.58%, Recall@5 = 79.80% and Recall@10 = 93.25%. These results demonstrate that relevant evidence is retrieved within the top-10 items for the vast majority of cases. The retrieval system performs effectively, ensuring that authoritative medical references are available for downstream evaluation of the model’s output. We believe this level of retrieval accuracy is sufficient to support the robustness of our evaluation process.

### High-risk escalation policy and human-in-the-loop safeguards

We design a collaborative system for DxDirector-7B to automate low-risk, high-cognitive steps while ensuring that high-risk situations are immediately surfaced to human clinicians. Concretely, we:

(i) formalize a taxonomy of clinical high-risk states from authoritative guidelines and institutional policies,
(ii) deploy a supervisory agent that continuously monitors DxDirector’s stepwise reasoning and I/O streams, and
(iii) enforce a fail-safe “stop-and-escalate” pathway that transfers control to physicians, preserves professional autonomy, and maintains a verifiable audit trail.

This shows the safety intent outlined in our method—physicians retain final decision-making and can take over at any time. In addition, escalation preserves timely risk management in alignment with our discussion on clinical relevance and safety.

#### Risk taxonomy and rule base

We compile a High-Risk Rule Set (HRRS) that enumerates triggerable states and observations. The HRRS is curated from hospital policies in the Grade 3A hospitals in China and is reviewed by medical specialists during development. The details of HRRS can be found in Supplementary materials. The HRRS is flexible enough to be adjusted according to the policies of different hospitals and updates to international standards. The taxonomy spans:

- Physiologic instability: patterns consistent with shock, severe respiratory compromise, or rapidly deteriorating neurologic status; hard thresholds for critical vitals (e.g., extreme hypotension/tachycardia, hypoxia unresponsive to routine measures) and alarm combinations (e.g., fever + neutropenia).
- Critical diagnostics: labs/imaging with immediate implications (e.g., markedly elevated troponin with ischemic symptoms; hyperkalemia with ECG changes; suspected intracranial hemorrhage wording on imaging reports).
- Time-critical syndromes and procedures: sepsis with organ dysfunction, acute coronary syndromes, stroke/TIA windows, anaphylaxis, ectopic pregnancy, acute abdomen, status epilepticus, meningitis, cauda equina signs, compartment syndrome.
- Safety, legal, and ethical flags: suicidal or homicidal ideation/plans, child/elder abuse signals, sexual assault, capacity concerns, legal/forensic holds, substance-related intoxication/withdrawal risks.
- Contraindicated actions: any model-proposed step that would exceed scope (e.g., initiating prescription-level interventions, consent-requiring procedures) or conflict with local policy.

Each HRRS item has: (i) a machine-readable definition with clear thresholds, (ii) severity class (Red—urgent and Green—routine), and (iii) escalation target (need human physicians or not). Details about the HRRS can be found in the Supplementary materials.

#### Runtime supervision via a monitoring agent

A supervisor LLM-based agent (GPT-4o) is attached to DxDirector’s event bus and ingests, in streaming fashion: (1) the current Deep Think block, (2) emitted [Question]/[Answer] pairs (including physician-supplied results), and (3) external artifacts provided by human physicians (e.g., “lab: K^+^ 6.8 mmol/L”, “CT report: ‘acute subarachnoid hemorrhage”’). Each HRRS item is organized as a single unit in a database, and a semantic ranking model (https://huggingface.co/Qwen/Qwen3-Reranker-8B) is used to find the Top-10 HRRS items that best matched the current input for the supervisor agent. The supervisor uses a structured prompt with three components:

- Context frame: The patient chief complaint, accumulated structured facts, and the current diagnostic goal.
- Rule frame: The Top-10 active HRRS items rendered as concise, testable checks (predicates and thresholds).
- Governance frame: Instructions that require the supervisor agent to determine whether a situation warrants a red risk level based on the current input and HRRS items.

At each step *t*, the supervisor evaluates ${{{\rm{SafetyScore}}}}_{t}\in \{{{\rm{Green}}},{{\rm{Red}}}\}$. For Red, the supervisor also proposes the minimum necessary context to show the physician, avoiding speculative content. This design integrates naturally with our structured, stepwise outputs and supports downstream accountability.

#### Escalation pipeline and fail-safe gating

When ${{{\rm{SafetyScore}}}}_{t}={{\rm{Red}}}$, DxDirector-7B transitions to Escalated Mode:

- Immediate halt: The diagnostic agent ceases autonomous planning beyond summarization; no further test ordering or interpretation recommendations are produced.
- Clinician escalation card: A concise artifact is emitted, containing: chief complaint, most recent structured facts, matched HRRS items and thresholds.
- Handoff and acknowledgment: All rights to the current diagnosis shall be transferred to the corresponding human clinician. The system awaits explicit physician acknowledgment before any further model activity.
- Audit log: Step-indexed records (inputs, reasoning headers, HRRS matches, timestamps, and human acknowledgments) are appended for traceability—aligning with our misdiagnosis accountability framework.

#### Human interface and accountability

Escalation cards reuse the structured, step-indexed layout of DxDirector’s final content to clearly separate physician-supplied observations from model-generated inferences and attaching literature for verifiability, thereby simplifying peer review and responsibility attribution. This symmetry is intentional: the same granularity that enables clinicians to trace decisions in ordinary cases supports rapid auditing during escalations.

#### Evaluation of the pipeline

Our evaluation consists of two settings: large-scale simulations and real clinical cases. As for simulation, we randomly select 500 samples from three datasets in section “Overview of experiments” (NEJM Clinicopathologic Cases, RareArena, and ClinicalBench) and modify the cases in these samples to incorporate red risks, such as adding laboratory test or imaging results that indicate danger based on the patient’s condition, or adding safety and legal issues. We evaluate the performance of our high-risk escalation pipeline on this dataset. The cases defined as red risk are positive samples. We calculate and report the following metrics to measure the performance of the risk escalation pipeline: (1) Precision = 92.47% (2) Recall = 98.59% (3) F1-Score = 95.43%. As for real clinical cases, our experiments are conducted on ten real-world emergency critically ill patients. Our pipeline successfully identifies risks in all cases and alert human clinicians for intervention, thereby ensuring clinical safety. These results demonstrate that our pipeline has a good ability to identify dangerous situations, ensuring the safety of DxDirector-7B in actual clinical use.

### Details about experiments

#### Baselines

The baselines of the experiments can be divided into two categories. One is the open source LLMs specifically optimized for medical scenarios and open source general-purpose LLMs:

1. **Meditron-70B**, it is a medical adapted LLM based on Llama-2-70B, with the continued pretraining on a comprehensively curated medical corpus, including selected PubMed articles, abstracts, and internationally-recognized medical guidelines.
2. **OpenbioLLM-70B**, it is an advanced LLM designed specifically for the biomedical domain based on Llama-3-70B. It achieves state-of-the-art performance on a wide range of biomedical tasks.
3. **Clinical Camel-70B**, it is a medically adapted LLM fine-tuned on the Llama-2 70B architecture using QLoRA. It is tailored for the medical and clinical research, capable of processing and generating relevant content.
4. **Meditron-176B**, it is a generalist medical LLM with 176 billion parameters, pre-trained on a large-scale medical text and real-world clinical records. It shows promising clinical diagnosis performance for cases with complete clinical information.

The other is the current most powerful commercial general-purpose large language models:

1. **GPT-4o**, it is a commercial general-purpose LLM developed by OpenAI. It shows better performance on clinical diagnosis than many medical adapted LLMs such as Meditron-70B, Clinical Camel-70B and Med-Palm-540B.
2. **o1-preview**, it is a commercial general-purpose LLM developed by OpenAI. Compared with GPT-4o, it demonstrates more powerful reasoning capability and recent study has shown that it surpasses human accuracy in making the final clinical diagnosis^21^.
3. **o3-mini**, it is a commercial general-purpose LLM developed by OpenAI. Compared with o1-preview, its reasoning ability has been upgraded again, surpassing GPT-4o and o1-preview in many complex tasks.
4. **Gemini-2.0-flash**, it is a commercial general-purpose LLM developed by Google. Its shows better performance than Google’s former PaLM 2.
5. **Deepseek-V3-671B**, it is a general-purpose developed by Deepseek. It surpasses GPT-4o in general language understanding and generation capabilities.
6. **Deepseek-R1-671B**, it is a general-purpose developed by Deepseek. Compared to Deepseek-V3, it possesses deep thinking capabilities and performs better on reasoning tasks.

#### Evaluation metrics

We propose four evaluation metrics in this paper:

1. **Accuracy of diagnosis**. This is a fully automatic evaluation metric driven by a LLM-based agent, which is used for four publicly available medical datasets including NEJM Clinicopathlogic Cases, RareArena, ClinicalBench and USMLE. Specifically, we use instructions to make the final diagnoses generated by LLMs conform to the format of “So the final answer is …”, so that we can extract the short and clear diagnoses generated by LLMs. For each sample, we compare the extracted diagnosis with the correct answer provided by the datasets to determine whether the generated diagnosis matches the correct answer. This is automatically done based on the gpt-4o-mini agent. We give gpt-4o-mini instructions and examples so that it can make the correct judgment. We statistically analyze the diagnostic accuracy to compare the capabilities of different LLMs.
2. **Number of clinical operations**. This metric calculates the average number of operations that human physicians are required to perform when different LLMs complete the full diagnostic process over the whole datasets (the lower the better), which quantifies the workload of human doctors.
3. **Effectiveness of clinical operations**. This metric calculates the average of the proportion of operations that are truly useful for making a diagnosis out of all requested operations over the whole datasets. We determine whether an operation is helpful for diagnosis by assessing whether it appears in the case report provided from medical specialists. In our large-scale automated evaluation, this is achieved by GPT-4o. GPT-4o is instructed to determine whether a given operation is mentioned in the case report provided by the medical specialists. Essentially, this task is similar to information extraction and matching, which is relatively straightforward for a LLM to handle, as it involves checking whether specific operations are explicitly referenced in the text. This metric reflects the accuracy of LLMs in determining which operations are necessary for diagnosis.
4. **Scoring referred from medical specialists**. This metric is applied in real-world diagnosis scenario. Evaluation by medical experts is an important way for us to understand the gap between AI and human physicians in real-world diagnosis. In order to establish an objective evaluation mechanism to prevent bias in human physicians’ scoring, we adopt Double-Blinded Adjudication, which makes LLMs and human physicians to give diagnoses to the same patient without seeing each other’s content. We introduce a third-party agent to score a LLM based on the degree of match between LLM’s diagnosis and human physicians’ diagnosis. The third-party agent is based on GPT-4o and Deepseek-V3, and the average of the scores given by them is taken as the actual score. The score range is between 0 and 10.

#### Statistical information

The error bars reflecting 95% confidence intervals are determined by non-parametric bootstrap procedure with 1000 samples. As for accuracy, we perform statistical significance tests utilizing the two-side McNemar test between DxDirector-7B and the top-3 baseline, with *p* value levels annotated on the bars. As for number of clinical operations, effective of clinical operations and scoring referred from specialists, we use two-side Mann-Whitney *U* test for statistical significance tests.

#### Implementations

For continued pre-training and instruction-tuning stage, we use DeepSpeed framework in zero stage 3 to train DxDirector-7B with full parameter fine-tuning on 4 Nvidia A100 80G GPUS. In continued pre-training, we follow Meditron^17^ to set *β*_1_ = 0.9, *β*_2_ = 0.95, eps = 10^−5^ for the AdamW optimizer. The learning rate is 3 × 10^−4^. The weight decay is 0.1. The batch size is 1. In instruction-tuning stage, we set *β*_1_ = 0.9, *β*_2_ = 0.95, eps = 10^−5^ for the AdamW optimizer. The learning rate is 9.65 × 10^−^^6^. The weight decay is 0. The batch size is 1 and training epochs is 3. In step-level strategy preference optimization, first, we input the same prefix to make DxDirector-7B generate multiple different replies by changing random seed at each generation, with sampling parameters as 0.6 temperature, 0.95 top-p and 20 top-k. After data construction, we use the open-source reinforcement learning framework trl https://github.com/huggingface/tr lto train our model with learning rate as 5 × 10^−7^, gradient accumulation steps as 8 and batch size as 1. During the evaluation, we cancel the random sampling setting so that the content generated by LLMs can be fully reproduced.

Code for data collection is written in Python (3.10) Joblib (1.4.2) numpy (1.26.4) scipy (1.14.0) openai (0.27.2). The training and evaluation framework used were written in Python (3.10), Pytroch (2.1.2), Transformers (4.37.2). We use DeepSpeed (0.12.6) and accelerate (0.21.0) to accelerate parallel training on multiple GPUs (4 Nvidia A100 80G GPUs). We use LLamaFactory (0.9.2) for online service of LLMs and use trl (0.9.6) for step-level preference optimization training.

#### Evaluation of agent for simulation

To automatically simulate this physician interaction on large-scale dataset, we implement an AI agent powered by GPT-4o, which receives real-time queries from the LLM, interprets the requested clinical information, and provides relevant data extracted from detailed clinical information to LLM, allowing LLM to continue reasoning (see prompts in Supplementary Fig. 21). In this setup, the simulated physician agent is largely a bounded extractor of facts from the case record in response to the model’s clinical queries (e.g., labs, symptoms, imaging findings), rather than an open-ended generator. This task is relatively straightforward for a LLM to handle^49,50^. In our audit on the test split, the agent’s information-extraction accuracy was 99.45%. Even under a conservative multi-turn assumption, the compounded accuracy remains high: 0.9945^10^ ≈ 94.63%. This level is comparable to reported ranges for practicing physicians on tasks such as chest X-ray and ECG interpretation, laboratory test interpretation and communication, and point-of-care ultrasound use^51–53^.

### Reporting summary

Further information on research design is available in the Nature Portfolio Reporting Summary linked to this article.

## Supplementary information

Supplementary Information

Reporting Summary

Peer Review file

## Source data

Source Data 1

Source Data 2

## Data Availability

The public available datasets are: RareArena: https://github.com/zhao-zy15/RareArena, NEJM Clinicopathologic Cases^21^: https://www.nejm.org/browse/nejm-article-category/clinical-cases?date=past5Years, ClinicalBench^25^: https://github.com/WeixiangYAN/ClinicalLab, USMLE and MedQA^22^: https://drive.google.com/file/d/1ImYUSLk9JbgHXOemfvyiDiirluZHPeQw/view. Source data are provided in FigShare (10.6084/m9.figshare.30688412). Source data are provided with this paper.

## Code availability

To prompt the future research, we release all source codes to train DxDirector-7B on Github (link is https://github.com/xsc1234/DxDirector). This ensures unrestricted access for anyone to use the code and model weights for any purpose, fostering future research and development. Combined with our detailed introduction to the method and the open source datasets, we believe that researchers will find it very easy to reproduce our work. The links to the codes that are developed by others are: Python^54^: https://github.com/python/cpython. Pytorch^55^: https://github.com/pytorch/pytorch. Transformers^56^: https://github.com/huggingface/transformers. DeepSpeed^57^: https://github.com/deepspeedai/DeepSpeed. accelerate^58^: https://github.com/huggingface/accelerate. LLamaFactory^59^: https://github.com/hiyouga/LlamaFactory. trl^60^: https://github.com/huggingface/trl.
