# Supplementary material for: DxDirector: an agentic large language model driving the full-process clinical diagnosis
Source: Nat Commun. 2026 Apr 23;17:5614. doi: 10.1038/s41467-026-71928-5 (PMC13315919; doi:10.1038/s41467-026-71928-5)
Supplement: Supplementary file 1 — Supplementary Information [file 41467_2026_71928_MOESM1_ESM.pdf]

Supplementary Information for Paper: DxDirector: An Agentic Large Language Model Driving the Full-process Clinical Diagnosis

Anonymous Author(s)

Contents

1 Method **2**

2 Accountability Mechanism **4**

3 Word Cloud for Clinical Operations **5**

4 Prompts for Data Construction **15**

5 Prompts for GPT-4o-based Agent to Simulate the Physicians **25**

6 Prompts for Full-process Clinical Diagnosis Setting in Baselines **25**

7 Quantitative Analysis of Human Physicians' Workload On USMLE **27**

8 Review Criteria and Agreement in Dataset Quality Assessment **28**

9 Extended Data **28**

1 Method

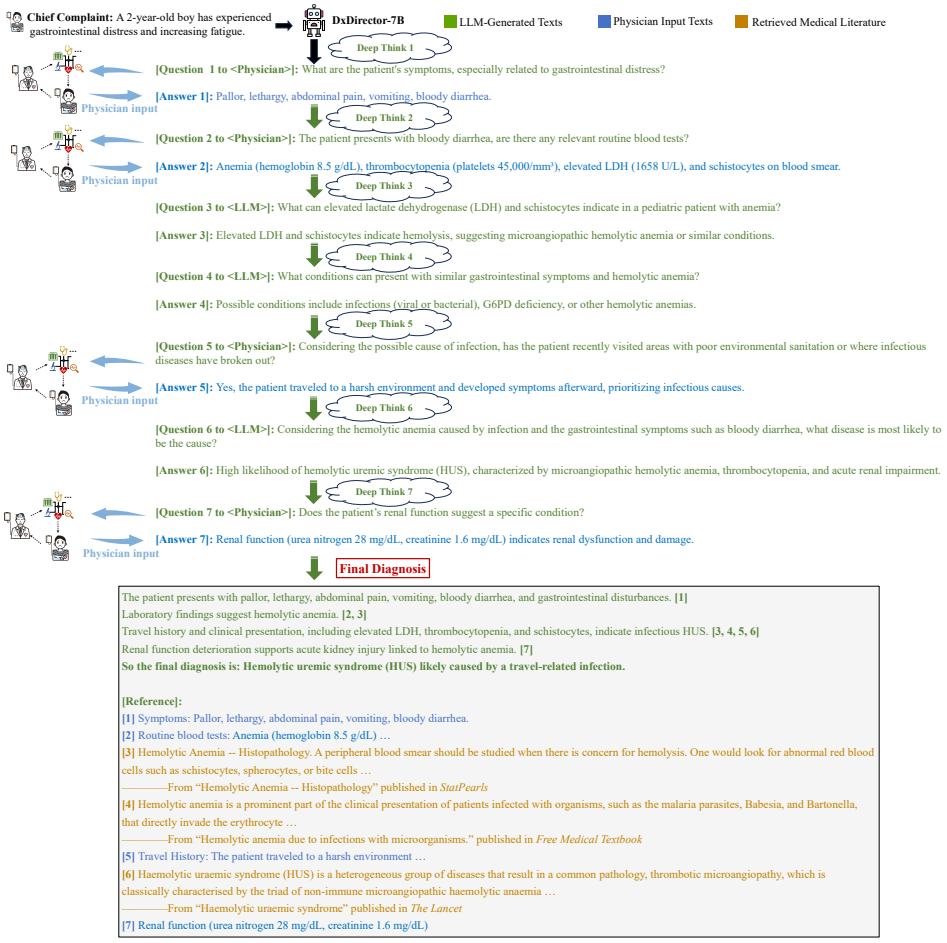

Figure 1: A case of DxDirector-7B performing the full-process diagnosis starting with only a chief complaint. Most of this process is driven by DxDirector-7B's step-by-step reasoning (green text) and physicians only need to follow its instructions to complete some basic clinical operations (blue text).

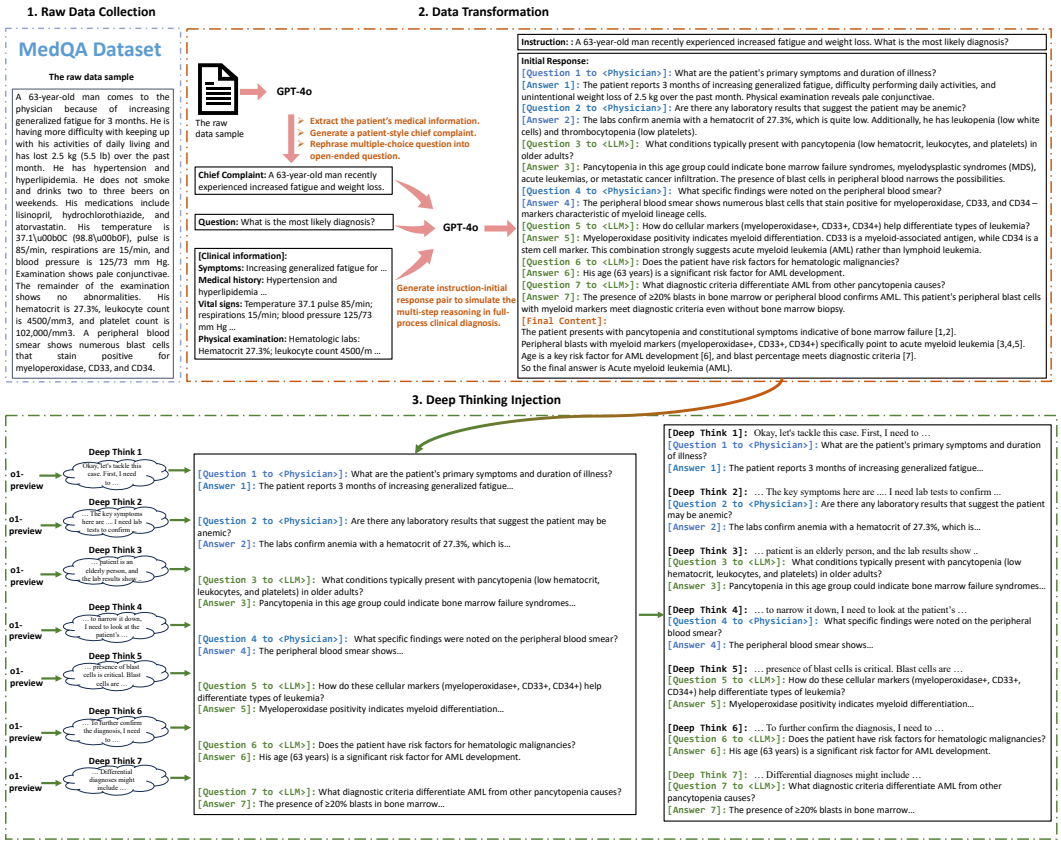

Figure 2: Pipeline of our data construction for instruction-tuning for full-process clinical diagnosis.

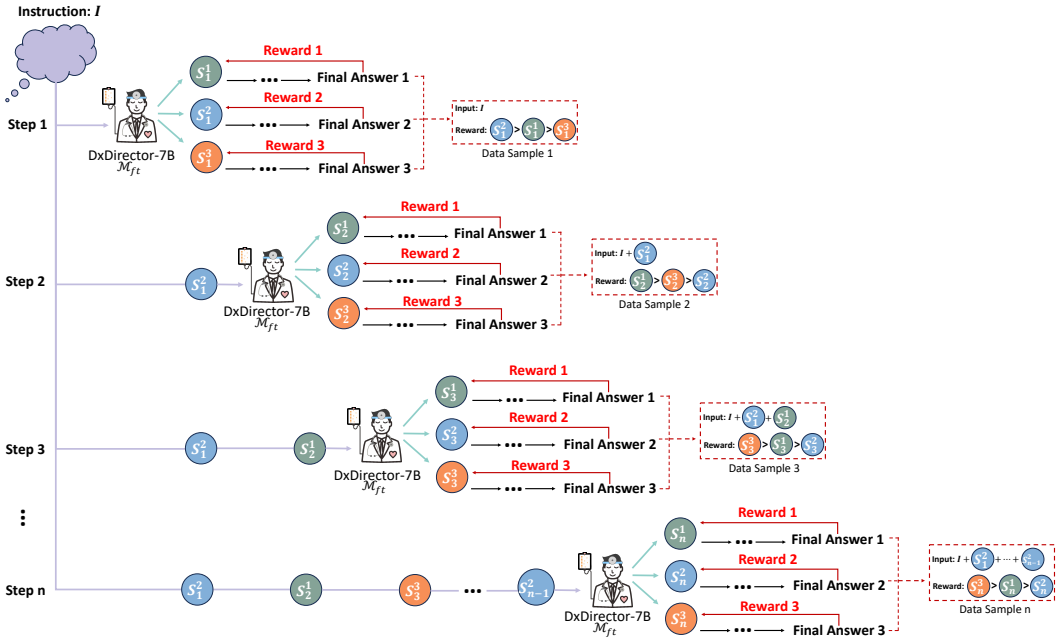

Figure 3: Pipeline of our data construction for Step-level Strategy Preference Optimization.

2 Accountability Mechanism

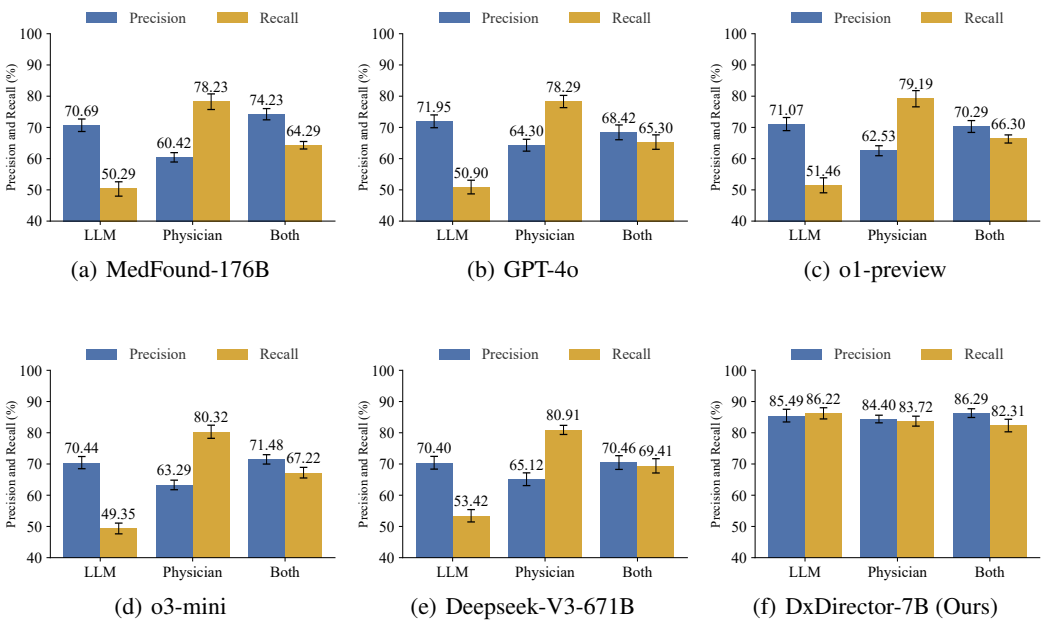

(a) MedFound-176B (b) GPT-4o (c) o1-preview

(d) o3-mini (e) Deepseek-V3-671B (f) DxDirector-7B (Ours)

Figure 4: The effectiveness of the accountability mechanism for the diagnosis content generated by different LLMs in misdiagnosis scenarios. **(a)** MedFound-176B. **(b)** GPT-4o. **(c)** o1-preview. **(d)** o3-mini. **(e)** Deepseek-V3-671B. **(f)** DxDirector-7B (Ours). The sample size is 1500. This is a classification task with 3 classes including the responsibilities of LLMs, human physicians or both of them. We use precision and recall for each task as the evaluation metrics. For example, bars for LLM class means the recall and precision for identifying the misdiagnoses caused by LLMs. Error bars reflect 95% confidence intervals determined by non-parametric bootstrap procedure with 1,000 samples.

3 Word Cloud for Clinical Operations

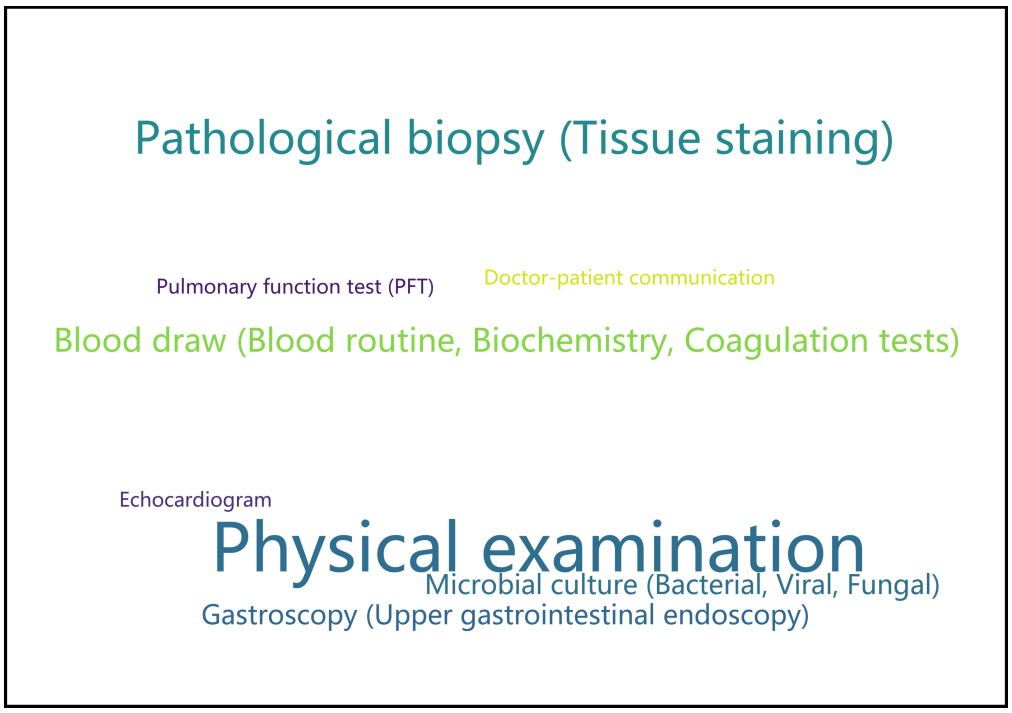

(a) Allergy and Immunology.

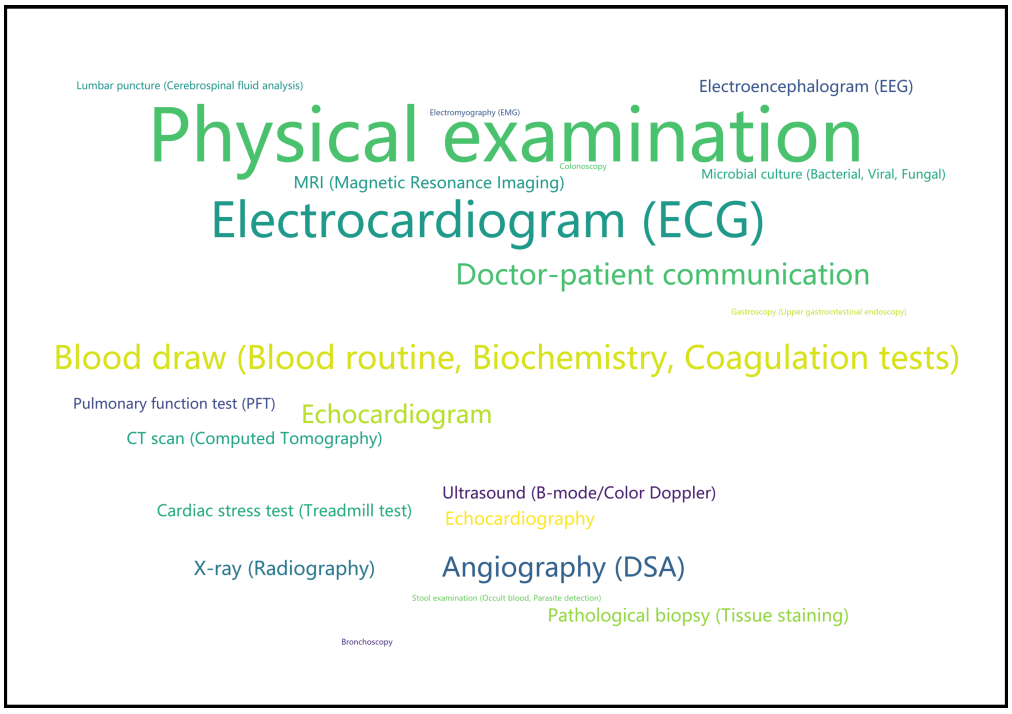

(b) Cardiology

Figure 5: Word cloud analysis of clinical operations that human physicians need to perform under the leadership of DxDirector-7B in **(a)** Allergy and Immunology, and **(b** Cardiology on RareArena and ClinicalBench datasets.

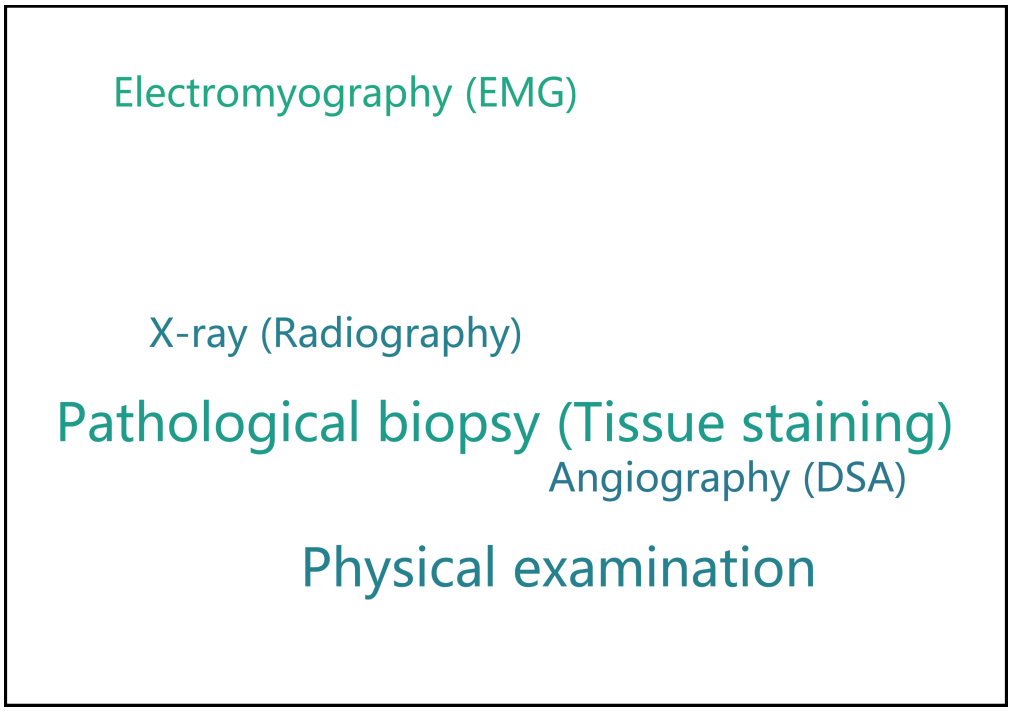

(a) Cardiothoracic Surgery

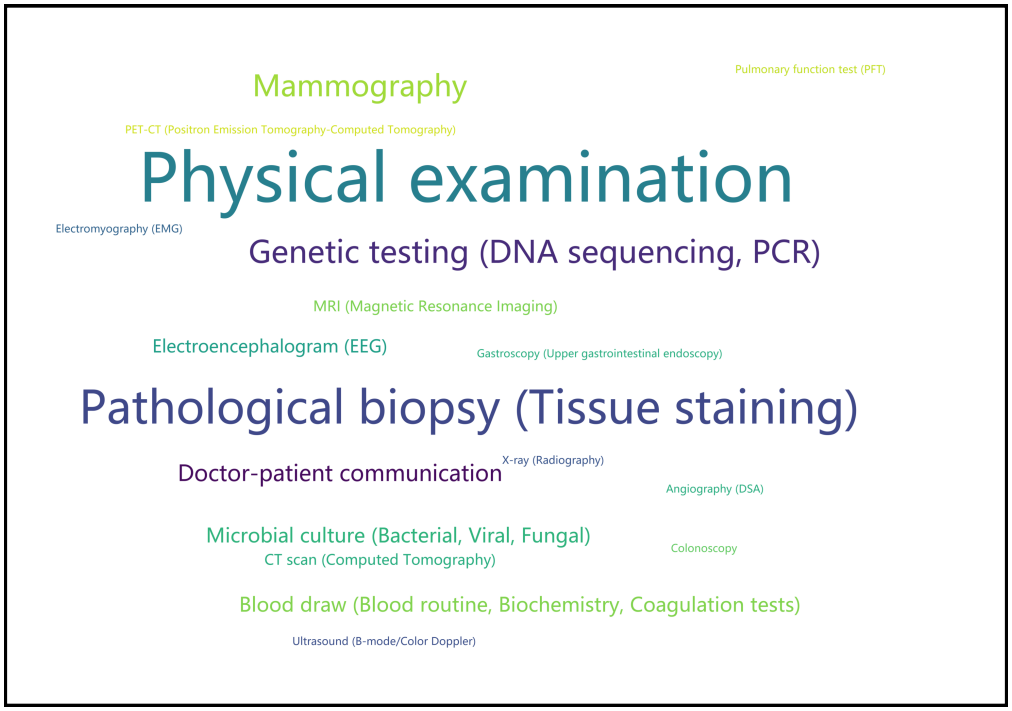

(b) Dermatology

Figure 6: Word cloud analysis of clinical operations that human physicians need to perform under the leadership of DxDirector-7B in **(a)** Cardiothoracic Surgery and **(b)** Dermatology on RareArena and ClinicalBench datasets.

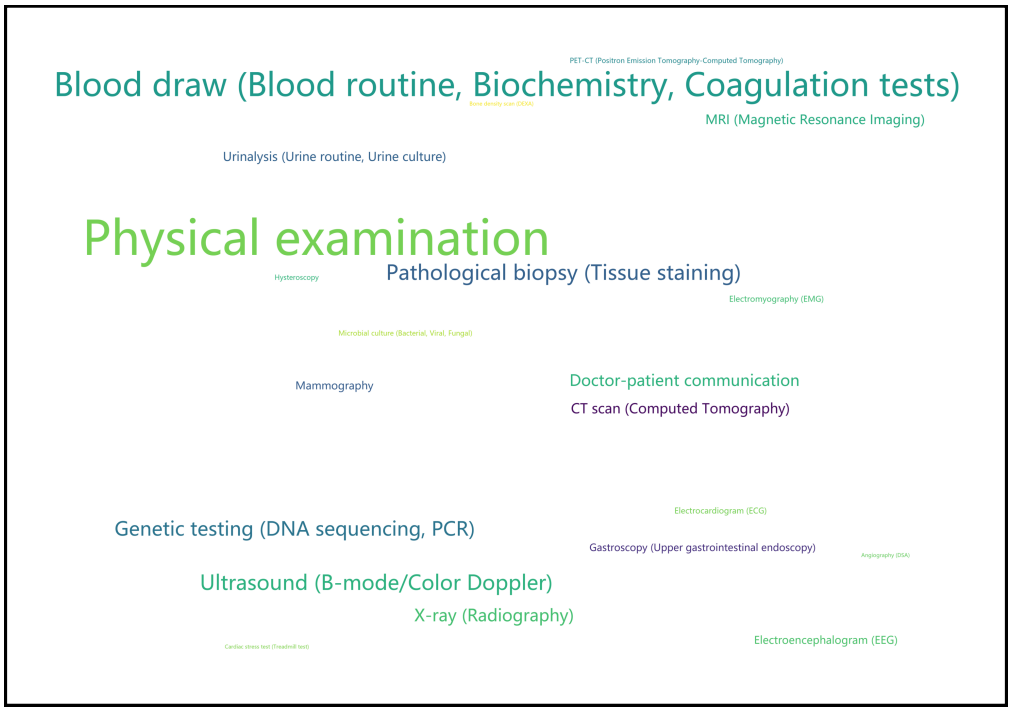

(a) Endocrinology

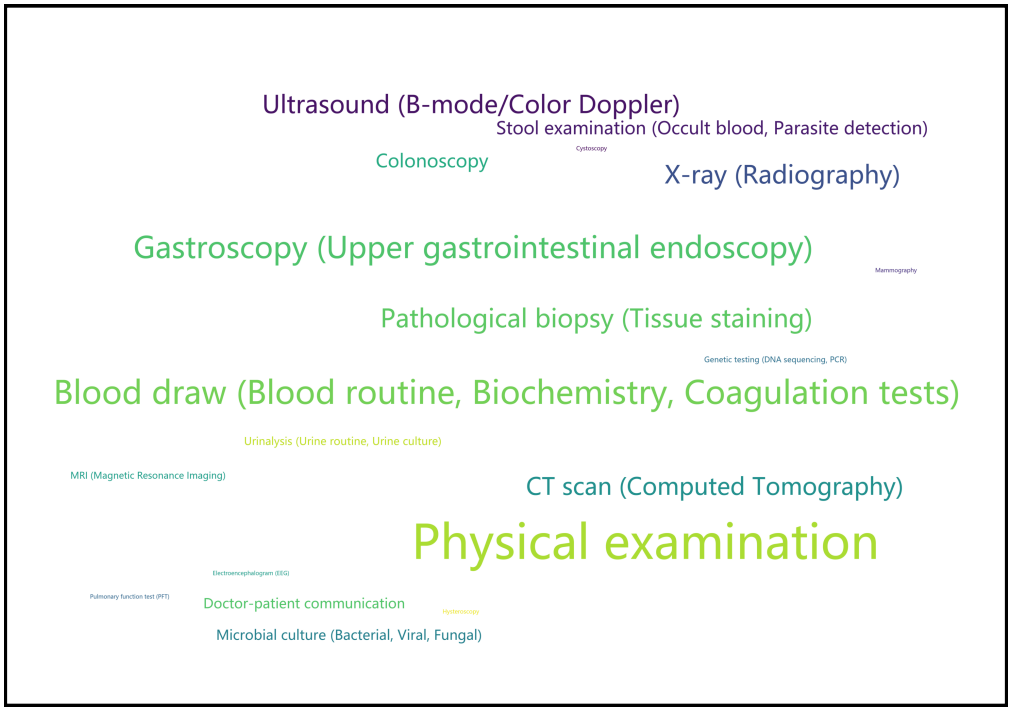

(b) Gastroenterology

Figure 7: Word cloud analysis of clinical operations that human physicians need to perform under the leadership of DxDirector-7B in **(a)** Endocrinology and **(b)** Gastroenterology on RareArena and ClinicalBench datasets.

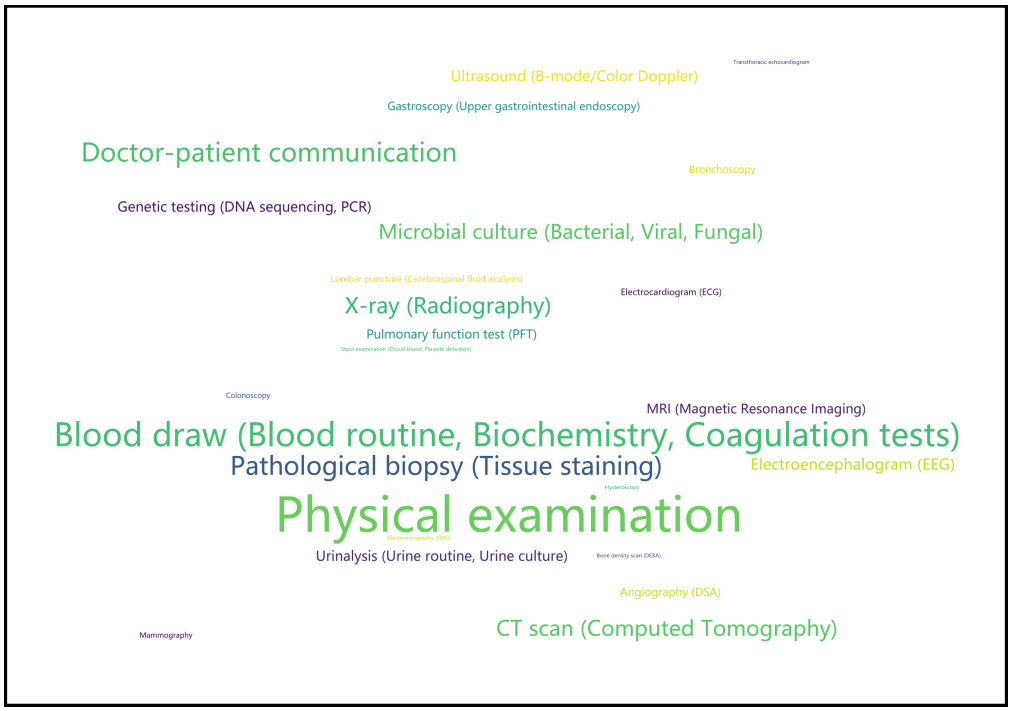

(a) Infectious Diseases

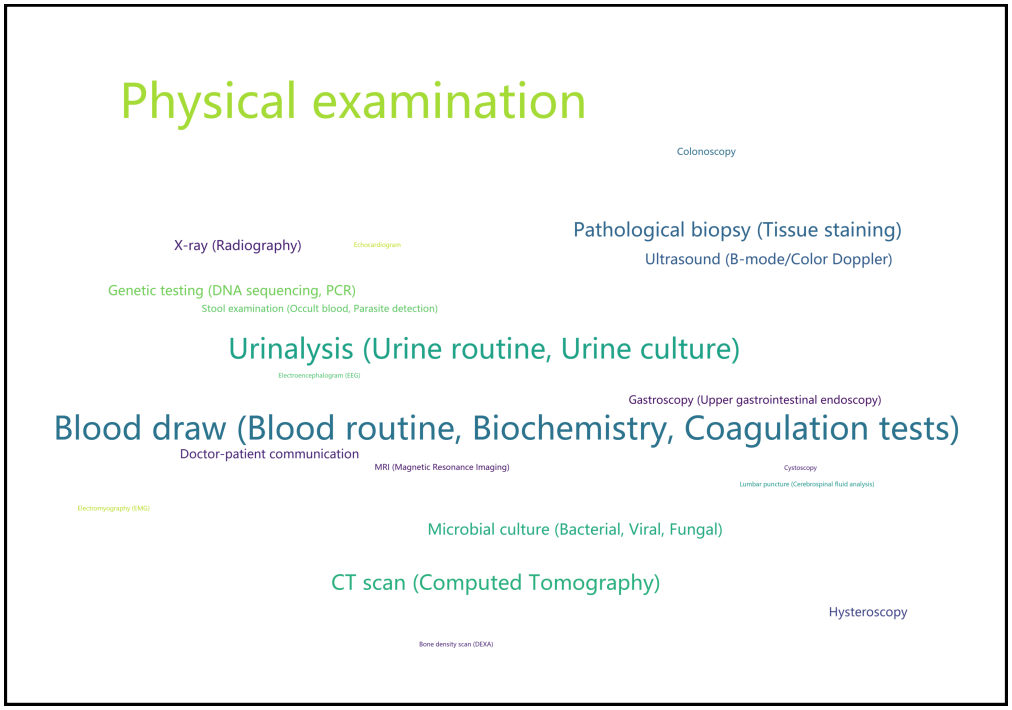

(b) Nephrology

Figure 8: Word cloud analysis of clinical operations that human physicians need to perform under the leadership of DxDirector-7B in **(a)** Infectious Diseases and **(b)** Nephrology on RareArena and ClinicalBench datasets.

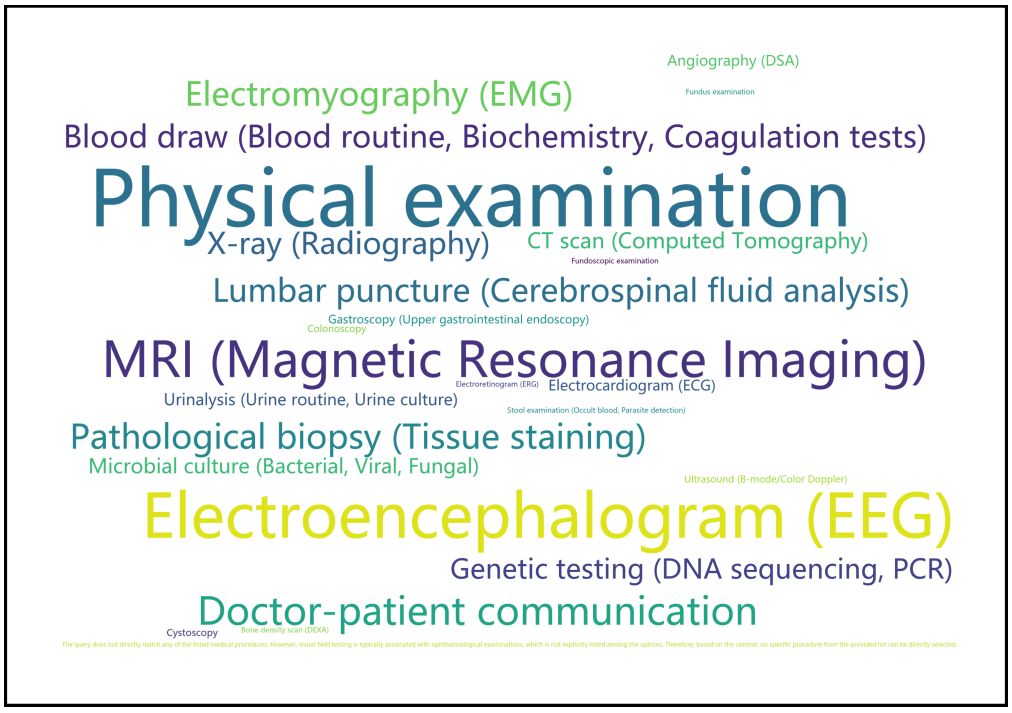

(a) Neurology

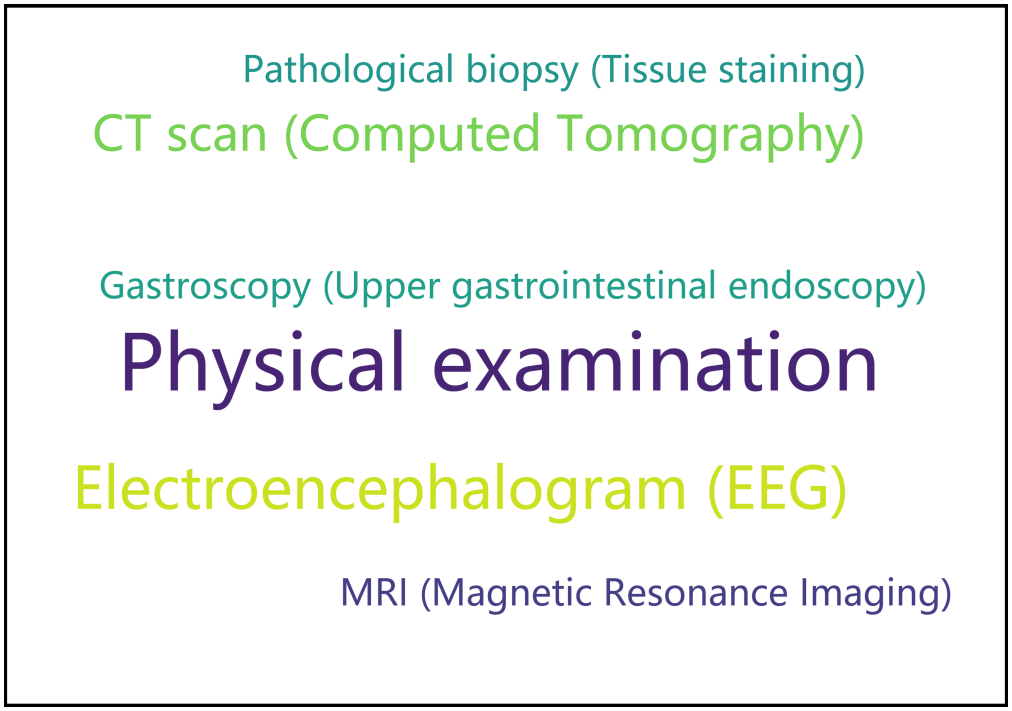

(b) Neurosurgery

Figure 9: Word cloud analysis of clinical operations that human physicians need to perform under the leadership of DxDirector-7B in **(a)** Neurology and **(b)** Neurosurgery on RareArena and ClinicalBench datasets.

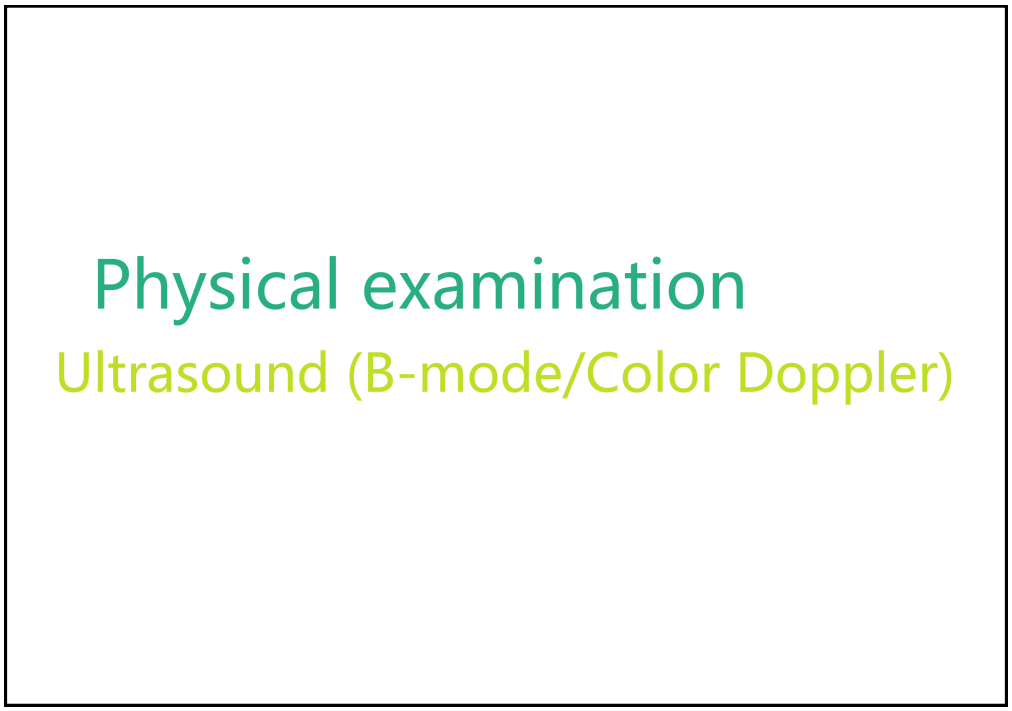

(a) Obstetrics

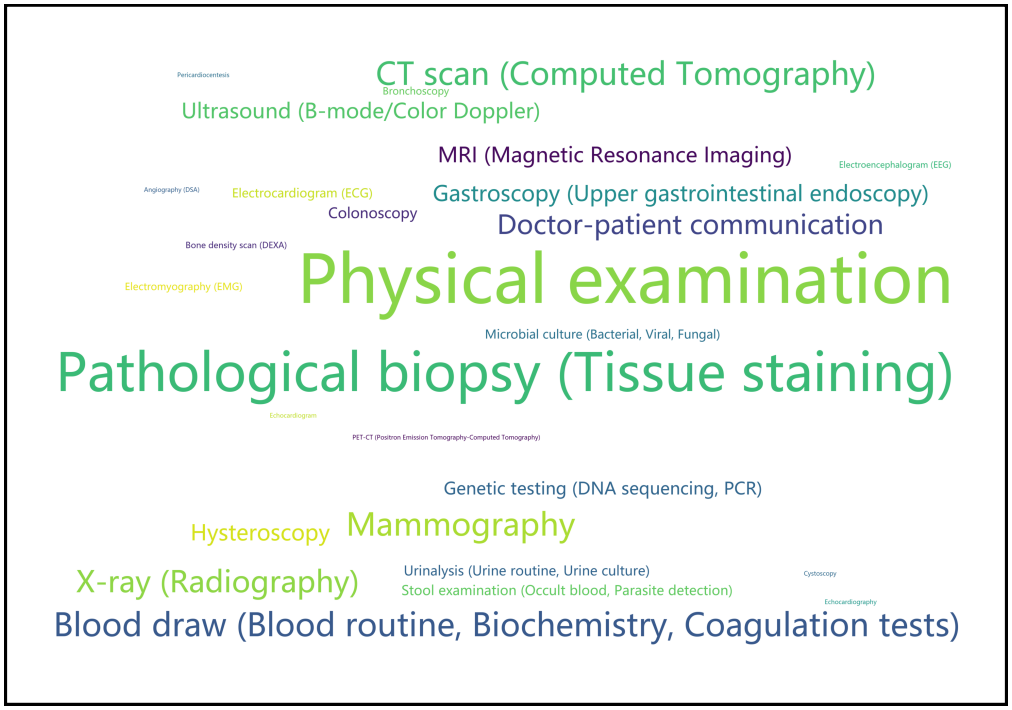

(b) Oncology

Figure 10: Word cloud analysis of clinical operations that human physicians need to perform under the leadership of DxDirector-7B in **(a)** Obstetrics and **(b)** Oncology on RareArena and ClinicalBench datasets.

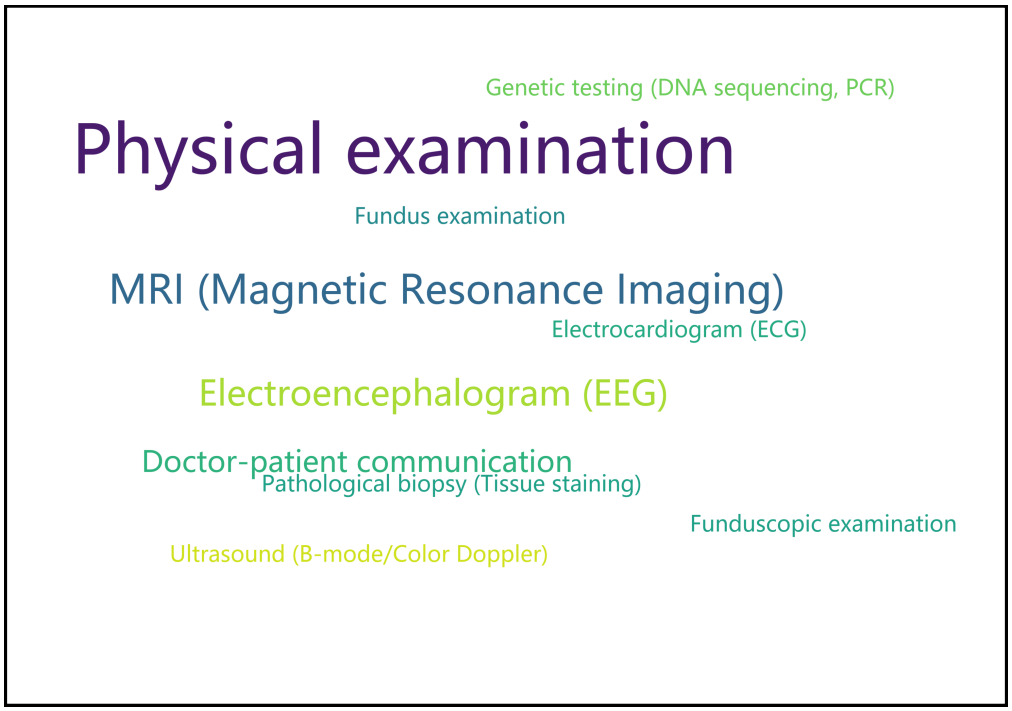

(a) Ophthalmology

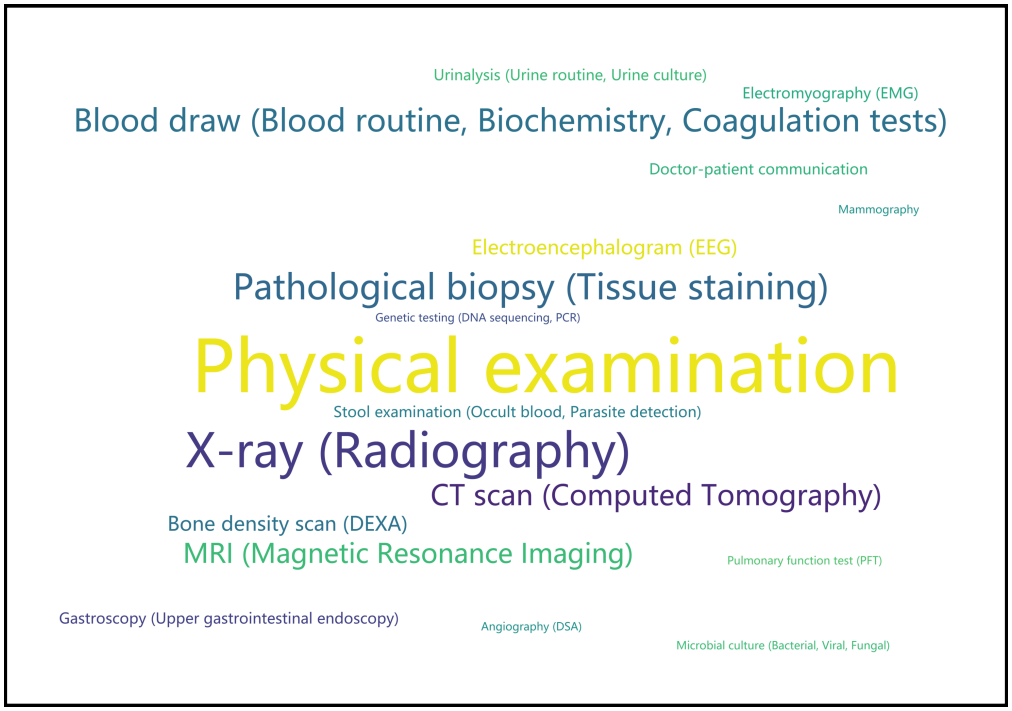

(b) Orthopedics

Figure 11: Word cloud analysis of clinical operations that human physicians need to perform under the leadership of DxDirector-7B in **(a)** Ophthalmology and **(b)** Orthopedics on RareArena and ClinicalBench datasets.

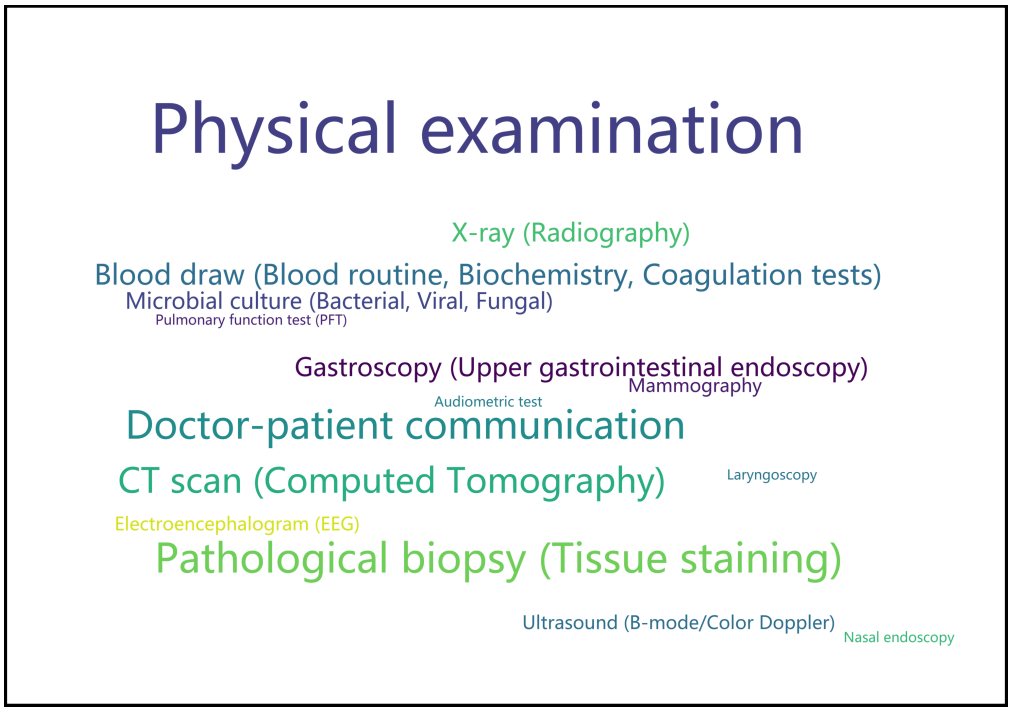

(a) Otolaryngology

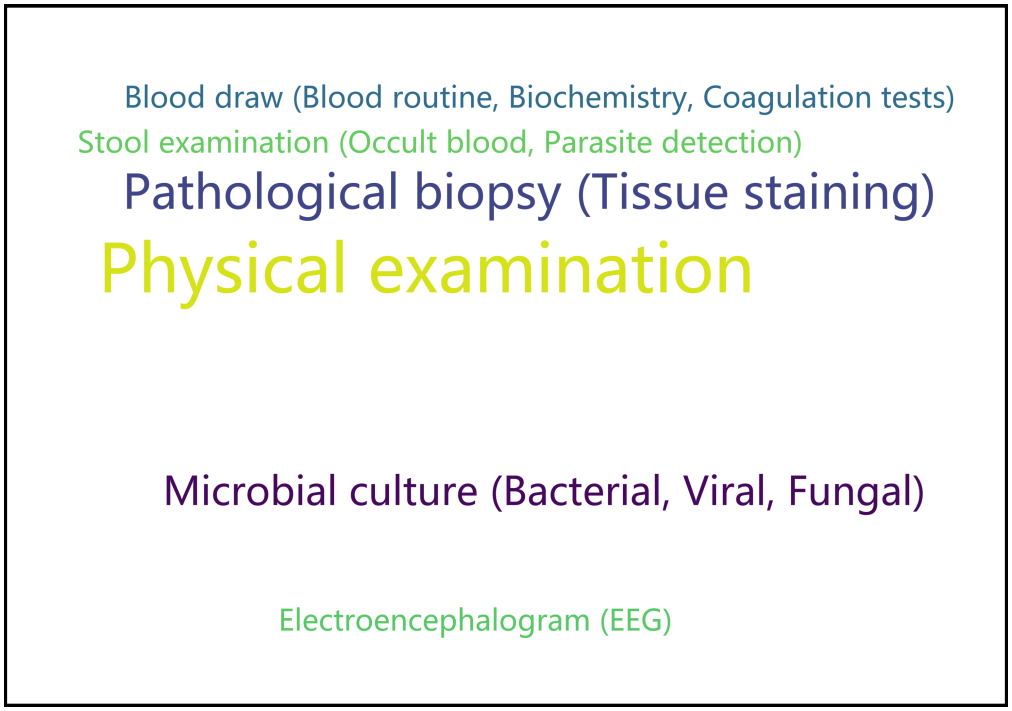

(b) Plastic Surgery

Figure 12: Word cloud analysis of clinical operations that human physicians need to perform under the leadership of DxDirector-7B in **(a)** Otolaryngology and **(b)** Plastic Surgery on RareArena and ClinicalBench datasets.

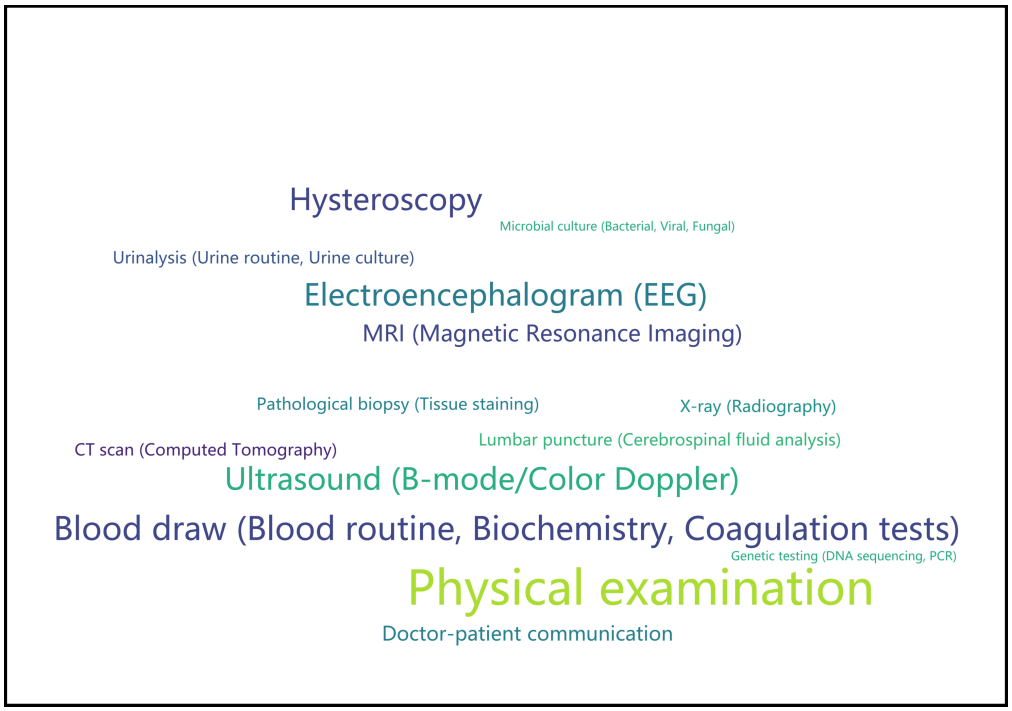

(a) Psychiatry

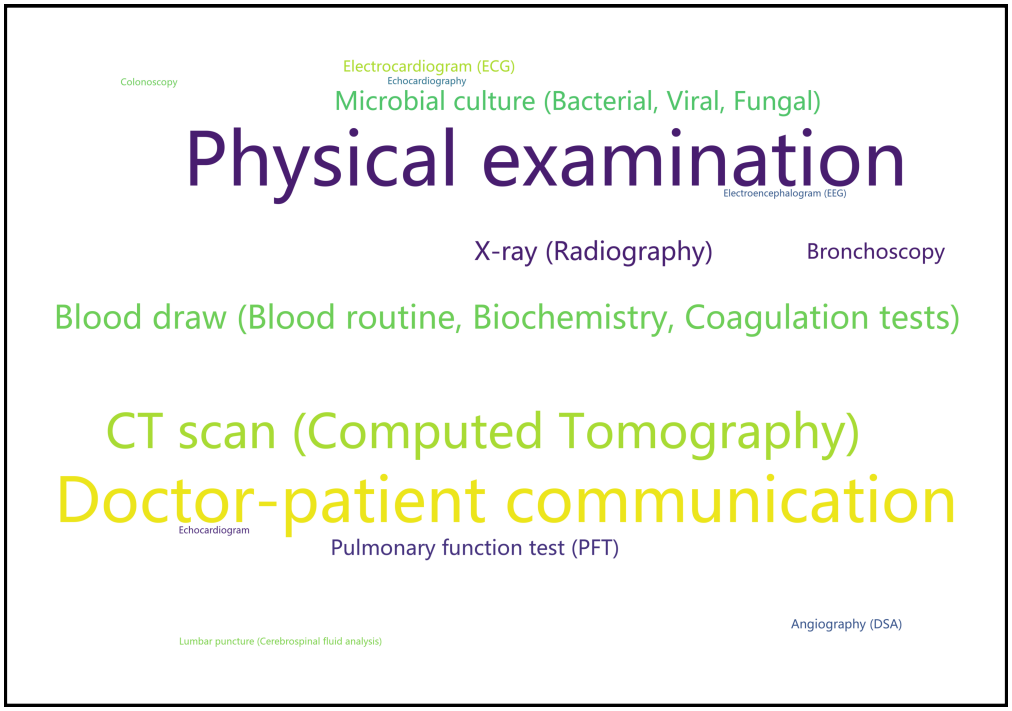

(b) Pulmonology

Figure 13: Word cloud analysis of clinical operations that human physicians need to perform under the leadership of DxDirector-7B in **(a)** Psychiatry and **(b)** Pulmonology on RareArena and ClinicalBench datasets.

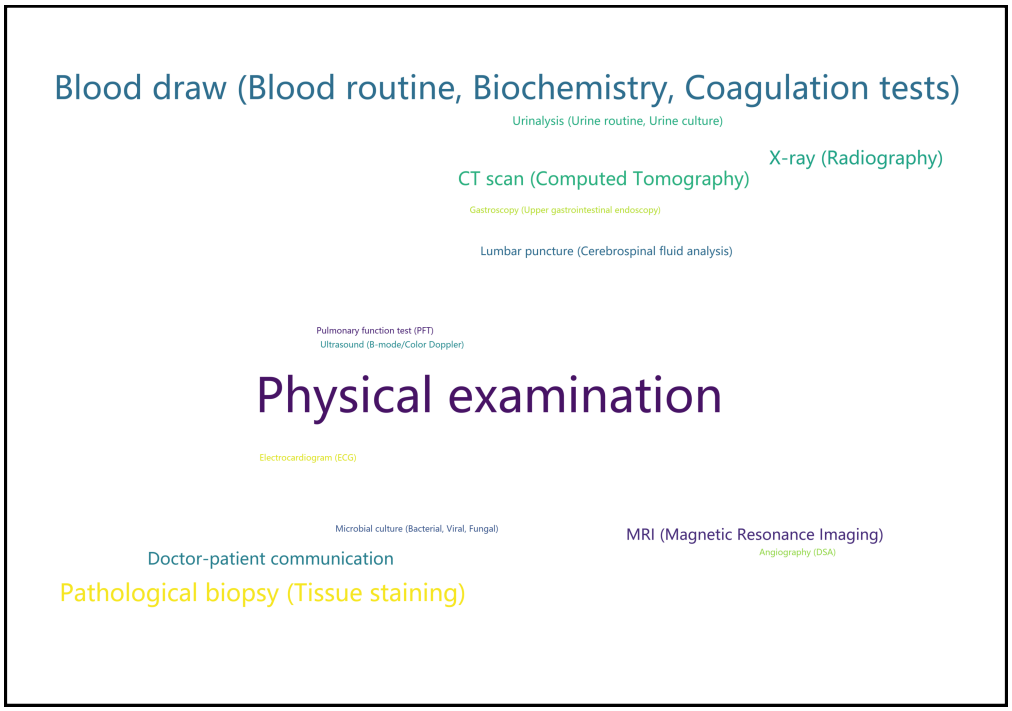

(a) Rheumatology

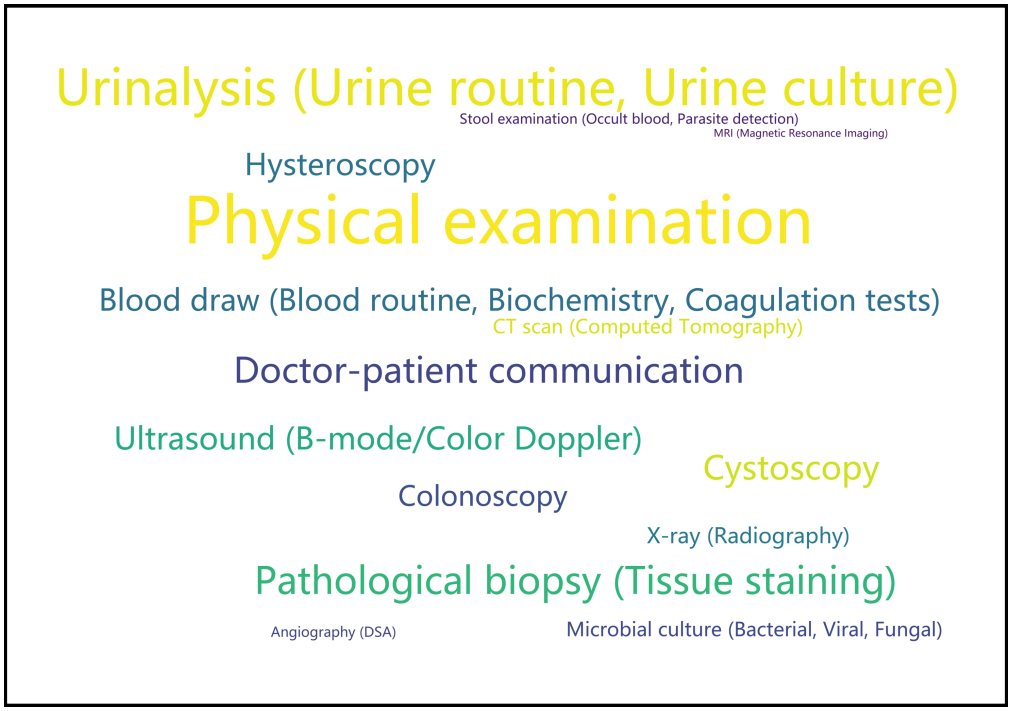

(b) Urology

Figure 14: Word cloud analysis of clinical operations that human physicians need to perform under the leadership of DxDirector-7B in **(a)** Rheumatology and **(b)** Urology on RareArena and ClinicalBench datasets.

4 Prompts for Data Construction

System Prompt
You are an experienced medical professional. Your current role is a physician who is examining a patient to answer the [Question]. The <Physician> description is in [Description of the patient].<br>You should pretend not to know the content in [symptoms, medical history, and test results] and get them through reasoning step by step in the chain to solve the [Question].<br>You should reason about the [Question] step by step and get content in [symptoms, medical history, and test results] during the reasoning process. Each question to access [symptoms, medical history, and test results] should arise naturally as part of the logical flow, ensuring that it aligns with the clinical reasoning process.<br>For each node of the chain, you should generate a Question, starting with [Question number]. You must give the answer for [Question number], starting with [Answer number].<br>Each node of the chain can be a Question and answer to the content in [symptoms, medical history, and test results], or a Question and answer to related medical knowledge.<br>You should mark the queries that ask for the content in [symptoms, medical history, and test results] with [Question to <Physician>]. You should mark the queries that ask for the related objective medical knowledge with [Question to <LLM>].<br>There should be strict logical connections between nodes, such as relevant medical knowledge or your medical experience<br>Emphasize again that you are now simulating a physician who is consulting a patient. You do not know all the information in [symptoms, medical history, and test results], and you must obtain them through step-by-step reasoning in the form of self-questioning and self-answering in the chain. Therefore, all [Question] cannot contain any [symptoms, medical history, and test results] information that is unknown in the current reasoning step.<br>If the reasoning is finished, you can generate the final answer for the [Question] by referring to the [Question]-[Answer] pairs, starting with [Final Content] and marking the number for each [Answer].<br>You must give the final answer by "So the final answer is ".<br>[Description of the patient]: {}<br>[symptoms, medical history, and test results]: {}<br>[Question]:{}

Figure 15: System prompt for data construction in instruction-tuning for full-process clinical diagnosis.

Example 1

Example 1 (Please strictly follow the format of [Question number]-[Answer number] (started with [xxx Question 1]), [Final Content] in the following example, and do not enter any content outside of the format. Emphasize again that you are now simulating a physician who is consulting a patient. You do not know all the information in [symptoms, medical history, and test results], and you must obtain them through step-by-step reasoning in the form of self-questioning and self-answering in the chain. Therefore, all [Question] cannot contain any [symptoms, medical history, and test results] information that is unknown in the current reasoning step.):
User: [Description of the patient]: A 62-year-old woman is currently under regular medical supervision to address her heart health and related concerns. She experiences occasional episodes that affect her overall comfort and is actively participating in her treatment plan to enhance her quality of life and manage her heart condition effectively.
[symptoms, medical history, and test results]: \nSymptoms: Lightheadedness and palpitations occurring episodically. \nMedical history: Myocardial infarction 6 months ago; NYHA class II chronic heart failure; diagnosed with grade I arterial hypertension 4 years ago. \nMedications: Aspirin 81 mg, atorvastatin 10 mg, enalapril 10 mg, and metoprolol 200 mg daily. \nVitals: Blood pressure 135/90 mm Hg; heart rate 125/min; respiratory rate 14/min; temperature 36.5\u00b0C (97.7\u00b0F). \nCardiopulmonary examination: Irregular heart rhythm and decreased S1 intensity. \nEchocardiography: Left ventricular ejection fraction of 39%.
[Question]: What drug is the best choice for rate control in this patient?

Assistant:
[Question 1 to <Physician>]: What are the <Physician> initial symptoms related to heart health?
[Answer 1]: The patient has some lightheadedness
[Question 2 to <Physician>]: Whether the patient has palpitations?
[Answer 2]: Yes, the patient has palpitations recently.
[Question 3 to < LLM>]: What condition could explain the <Physician> symptoms of lightheadedness and palpitations?
[Answer 3]: Lightheadedness and palpitations could be caused by arrhythmias, such as atrial fibrillation (AF), or other cardiovascular issues like heart failure exacerbation or ischemia. Let's confirm if the patient has signs of arrhythmia.
[Question 4 to <Physician>]: What is the patient's heart rate and rhythm?
[Answer 4]: The patient's heart rate is 125 bpm, and her rhythm is irregular. This supports a diagnosis of atrial fibrillation (AF) causing rapid ventricular response. Rate control is a priority in managing this condition.
[Question 5 to <Physician>]: Does the patient have underlying cardiac conditions that influence treatment decisions?
[Answer 5]: Yes, the patient has a history of myocardial infarction 6 months ago, NYHA class II chronic heart failure with reduced ejection fraction (HFrEF, EF 39%), and hypertension. These conditions impact the choice of medications for rate control.
[Question 6 to <Physician>]: Are there contraindications to using certain rate control medications in this patient?
[Answer 6]: Non-dihydropyridine calcium channel blockers like verapamil and diltiazem are contraindicated in HFrEF due to their negative inotropic effects, which can worsen heart failure. Propafenone, a rhythm control drug, is also unsuitable due to proarrhythmic risks in structural heart disease.
[Question 7 to <Physician>]: What medications is the patient currently taking, and do they address rate control?
[Answer 7]: The patient is on metoprolol 200 mg daily, which is a beta-blocker commonly used for rate control in AF and heart failure. However, her heart rate of 125 bpm suggests suboptimal rate control, indicating the need for an additional or alternative therapy.
[Question 8 to <LLM>]: Why might digoxin be a good option in this patient?
[Answer 8]: Digoxin slows the ventricular rate in AF by enhancing vagal tone without adversely affecting cardiac output, making it particularly suitable for patients with HFrEF. It can be used as adjunctive therapy to improve rate control in this patient.
[Final Content]:
The patient has symptoms of dizziness and palpitations. [1,2]
The patient has atrial fibrillation with rapid ventricular response (heart rate 125 bpm) [4].
She has underlying HFrEF and a history of myocardial infarction, influencing the choice of rate control medications [5].
Non-dihydropyridine calcium channel blockers and propafenone are contraindicated in this patient [6].
Atenolol is less effective in heart failure compared to her current beta-blocker (metoprolol) [7].
Digoxin is a safe and effective option for rate control in AF with HFrEF [8].
So the final answer is Digoxin.

Figure 16: The first example in prompt for data construction in instruction-tuning for full-process clinical diagnosis.

Example 2

Example 2 (Please strictly follow the format of [Question number]-[Answer number] (started with [xxx Question 1]), [Final Content] in the following example, and do not enter any content outside of the format. Emphasize again that you are now simulating a physician who is consulting a patient. You do not know all the information in [symptoms, medical history, and test results], and you must obtain them through step-by-step reasoning in the form of self-questioning and self-answering in the chain. Therefore, all [Question] cannot contain any [symptoms, medical history, and test results] information that is unknown in the current reasoning step.):
User: [Description of the patient]: A 46-year-old woman is currently undergoing treatment for a condition that has significantly affected her vision and overall well-being. She has been experiencing various symptoms that have emerged over the past few months, and she is in the process of working with her healthcare team to address these challenges while also managing some recent positive changes in her lifestyle
[symptoms, medical history, and test results]: Symptoms: 2-week history of diplopia; ocular pain when reading; 3-month history of amenorrhea, hot flashes, and increased sweating; fine resting tremor of the hands. \nMedical history: Overweight all her adult life; lost 6.8-kg (15-lb) in the past 2 months. \nVital signs: Pulse 110/min; blood pressure 148/98 mm Hg. \nPhysical examination: Moist palms; nontender, enlarged thyroid gland; ophthalmologic findings of globe prominence, bilateral lid retraction, conjunctival injection, and inability to converge the eyes; pain-free extraocular muscle movement; visual acuity 20/20 bilaterally; deep tendon reflexes 3+ with shortened relaxation phase.
[Question]: What is the most likely cause of this <Physician> ocular complaints?

Assistant:
[Question 1 to <Physician>]: What are the <Physician> initial symptoms related to her vision and well-being?
[Answer 1]: The patient reports a 2-week history of diplopia (double vision) and ocular pain when reading.
[Question 2 to <LLM>]: What disease may cause binocular double vision and eye pain?
[Answer 2]: Diplopia and ocular discomfort can be caused by various conditions, including thyroid eye disease (Graves' ophthalmopathy), cranial nerve palsies, and other orbital pathologies. These disorders can cause double vision and eye discomfort. They may also be accompanied by systemic symptoms such as hot flashes and excessive sweating.
[Question 3 to <Physician>]: Does the patient has systemic symptoms, such as hot flashes, excessive sweating, menstrual disorders, etc.?
[Answer 3]: Yes, the patient reports the systemic symptoms like amenorrhea, hot flashes, and increased sweating.
[Question 4 to <Physician>]: Given her systemic symptoms, it may be worth investigating if thyroid dysfunction is involved. Is there any evidence of thyroid dysfunction in the <Physician> clinical presentation?
[Answer 4]: Yes, the patient has an enlarged, nontender thyroid gland and associated symptoms indicating hyperthyroidism, such as increased sweating and weight loss, which is often seen in Graves' disease.
[Question 5 to <Physician>]: What specific findings in this patient suggest the diagnosis of Graves' disease?
[Answer 5]: The patient exhibits exophthalmos (globe prominence), bilateral lid retraction, and an inability to converge her eyes, all of which are indicative of thyroid-associated ophthalmopathy, commonly associated with Graves' disease.
[Question 6 to <LLM>]: What role do glycosaminoglycans play in the ocular manifestations associated with Graves' disease?
[Answer 6]: Glycosaminoglycans, ely hyaluronic acid, accumulate in the orbit due to autoimmune processes in Graves' disease, leading to edema and expansion of extraocular muscles, which can result in diplopia and other ocular symptoms.
[Question 7 to <LLM>]: Given the findings, what is the most likely underlying cause of this <Physician> ocular complaints?
[Answer 7]: The ocular complaints primarily stem from the accumulation of glycosaminoglycans in the orbit as a result of Graves' disease, which affects the extraocular muscles and the surrounding tissues.
[Final Content]:
The patient reports diplopia and ocular pain, signifying possible ocular pathology [1,3].
She has symptoms consistent with hyperthyroidism and Graves' disease, including globe prominence and other ocular signs [5].
Glycosaminoglycan accumulation typically occurs in the context of Graves' disease, leading to the associated ocular manifestations such as diplopia [6,7].
So the final answer is Glycosaminoglycan accumulation in the orbit.

Figure 17: The second example in prompt for data construction in instruction-tuning for full-process clinical diagnosis.

Example 3

Example 3 (Please strictly follow the format of [Question number]-[Answer number] (started with [xxx Question 1]), [Final Content] in the following example, and do not enter any content outside of the format. Emphasize again that you are now simulating a physician who is consulting a patient. You do not know all the information in [symptoms, medical history, and test results], and you must obtain them through step-by-step reasoning in the form of self-questioning and self-answering in the chain. Therefore, all [Question] cannot contain any [symptoms, medical history, and test results] information that is unknown in the current reasoning step.):
User: [Description of the patient]: A 7-year-old girl is currently under medical care due to a recent illness characterized by discomfort and visible signs on her skin. Following an incident with a cat bite, her parents have sought help to address her symptoms and ensure a full recovery.
[symptoms, medical history, and test results]: Symptoms: Red papules over left thigh; swelling in right axilla; fever of 38.6\u00b0C (101.4\u00b0F). \nMedical history: Cat bite on left thigh 2 weeks ago. \nVitals: Pulse 90/min; respirations 22/min. \nPhysical examination: Hepatosplenomegaly; healing area of erythema on left hand. \nLaboratory studies: Hemoglobin 12.9 gm/dL; leukocyte count 9,300/mm\u00b3; platelet count 167,000/mm\u00b3; ESR 12 mm/hr. \n\n
[Question]: What is the most appropriate next step in management?

Assistant:
[Question 1 to <Physician>]: What are the patient's initial symptoms following the cat bite?
[Answer 1]: The patient has red papules over her left thigh, swelling in the right axilla, and a fever of 38.6°C (101.4°F).
[Question 2 to <LLM>]: What are common complications or infections that can arise from a cat bite?
[Answer 2]: Cat bites can lead to infections such as cellulitis or abscess formation, often due to bacteria like Pasteurella multocida, and can also cause lymphadenopathy. Monitoring for systemic signs of infection, ely in pediatrics, is crucial.
[Question 3 to <Physician>]: How long ago did the cat bite occur?
[Answer 3]: The cat bite occurred 2 weeks ago.
[Question 4 to <LLM>]: Given the time frame and symptoms, what is the potential concern regarding her current condition?
[Answer 4]: The timing of the symptoms and the cat bite raises suspicion for an infection that may require intervention, ely if the patient is showing signs of systemic infection such as fever and localized swelling.
[Question 5 to <Physician>]: What does the physical examination reveal, particularly concerning the lymphatic system?
[Answer 5]: The physical examination shows hepatosplenomegaly and a healing area of erythema on the left hand, indicating potential systemic involvement and local infection at the bite site.
[Question 6 to <Physician>]: Based on her blood test results, what are the key findings to note?
[Answer 6]: The laboratory studies show a hemoglobin level of 12.9 gm/dL, a leukocyte count of 9,300/mm³, a platelet count of 167,000/mm³, and an erythrocyte sedimentation rate (ESR) of 12 mm/hr. This indicates a relatively normal leukocyte count and platelet count, with slight inflammation suggested by the ESR.
[Question 7 to <LLM>]: Given this information, what is the appropriate initial step in management for a child with symptoms following a potential cat bite infection?
[Answer 7]: The most appropriate initial step in management would be to initiate empiric antibiotic therapy, addressing the risk of infection from the cat bite, given the child's symptoms and the recent history.
[Final Content]:
The patient has red papules on her thigh, swelling in the right axilla, and a fever, indicating possible infection following a cat bite [1].
She has hepatosplenomegaly and erythema, suggesting systemic infection [5].
Her laboratory findings largely within normal limits indicate no severe leukopenia or thrombocytopenia, but the slight increase in ESR suggests inflammation [6].
Given the clinical context and the symptoms following the cat bite, empiric antibiotic therapy is warranted [7].
So the final answer is Initiate empiric antibiotic therapy.

Figure 18: The third example in prompt for data construction in instruction-tuning for full-process clinical diagnosis.

Example 4

Example 4 (Please strictly follow the format of [Question number]-[Answer number] (started with [xxx Question 1]), [Final Content] in the following example, and do not enter any content outside of the format. Emphasize again that you are now simulating a physician who is consulting a patient. You do not know all the information in [symptoms, medical history, and test results], and you must obtain them through step-by-step reasoning in the form of self-questioning and self-answering in the chain. Therefore, all [Question] cannot contain any [symptoms, medical history, and test results] information that is unknown in the current reasoning step.):
User: [Description of the patient]: A 35-year-old woman is experiencing ongoing treatment for a complex condition that has contributed to fatigue and discomfort, affecting her daily life and mobility. She has noticeable skin changes and is being carefully monitored by her healthcare team to address her symptoms and enhance her quality of life.
[symptoms, medical history, and test results]: Symptoms: 3-month history of progressive fatigue; shortness of breath; pain in knees and ankles; mild hepatomegaly; tender, red nodules on shins; purple, indurated lesions on nose, nasolabial fold, and cheeks. \nTest results: Liver biopsy shows scattered aggregations of multinucleated giant cells with cytoplasmic inclusions and eosinophilic needle-shaped structures arranged in a star-like pattern.
[Question]: What is the most likely cause of this <Physician> symptoms?

Assistant:
[Question 1 to <Physician>]: What are the <Physician> key symptoms related to her condition affecting daily life and mobility?
[Answer 1]: The patient has a 3-month history of progressive fatigue, shortness of breath, pain in her knees and ankles, mild hepatomegaly, and tender red nodules on her shins, along with purplish, indurated lesions on her nose, nasolabial fold, and cheeks.
[Question 2 to <LLM>]: What could cause the combination of progressive fatigue, joint pain, mild hepatomegaly, and specific skin lesions?
[Answer 2]: Conditions such as systemic lupus erythematosus (SLE), dermatomyositis, or other autoimmune diseases could present with these symptoms. Specifically, we should consider if there is a connection between the skin findings and the systemic symptoms.
[Question 3 to <Physician>]: How do the specific skin lesions correlate with systemic conditions?
[Answer 3]: The tender red nodules on the shins and purple indurated lesions, particularly on sun-exposed areas like the nose and cheeks, suggest possible dermatomyositis or another connective tissue disease.
[Question 4 to <Physician>]: Are there any notable findings from the liver biopsy that could help identify the underlying cause of these symptoms?
[Answer 4]: Yes, the liver biopsy shows scattered aggregations of multinucleated giant cells with cytoplasmic inclusions and eosinophilic needle-shaped structures arranged in a star-like pattern. This finding is characteristic of a specific condition.
[Question 5 to <LLM>]: Given the histological findings, what condition could the liver biopsy results indicate?
[Answer 5]: The biopsy results strongly suggest a diagnosis of non-alcoholic steatohepatitis (NASH) or possibly a metabolic condition such as a form of hepatitis, particularly one associated with autoimmune features.
[Question 6 to <LLM>]: What systemic condition is associated with similar multicellular immune response features that may relate to the reported symptoms?
[Answer 6]: Conditions like sarcoidosis or other granulomatous diseases could present with multinucleated giant cells and may explain some of the systemic symptoms alongside skin manifestations.
[Final Content]:
The patient presents with progressive fatigue, joint pain, mild hepatomegaly, and distinctive skin lesions, indicating a complex systemic condition [1,2,3].
The liver biopsy reveals aggregations of multinucleated giant cells and eosinophilic structures, suggesting a specific etiology potentially related to an autoimmune or granulomatous process [4,5].
Given the skin and systemic symptoms, autoimmune conditions or metabolic syndromes like NASH emerge as underlying causes with multinucleated giant cell formation hinting at systemic involvement [6].
So the final answer is likely an autoimmune condition such as dermatomyositis or non-alcoholic steatohepatitis (NASH) leading to systemic symptoms, liver involvement, and skin changes.

Figure 19: The fourth example in prompt for data construction in instruction-tuning for full-process clinical diagnosis.

System Prompt
This is a step-by-step reasoning process of a physician in a clinical consultation, organized in the form of [Question]-[Answer] pairs.<br>Please add the physician's thinking before each [Question] to make the logical connection between [Question] smoother and tighter. This thinking should be marked with <sub-think> </sub-think>.<br>Please note that you cannot change anything else, you can only add thoughts in <sub-think> to make the logic tighter and smoother!<br>Please note that you cannot change anything else, you can only add thoughts in <sub-think> to make the logic tighter and smoother!<br>Please note that you cannot change anything else, you can only add thoughts in <sub-think> to make the logic tighter and smoother!

Figure 20: System prompt for deep thinking injection in data construction for full-process clinical diagnosis.

Example 1

Example 1 (Please strictly follow the format of [Question number]-[Answer number] (started with [xxx Question 1]), [Final Content] in the following example, and do not enter any content outside of the format.):

User:

[Description of the patient]: A 62-year-old woman is currently receiving treatment for a respiratory condition that has contributed to her feelings of fatigue and difficulty breathing, especially with exertion. With a significant history as a nurse and various chronic health concerns, she is actively working with her healthcare team to manage and improve her respiratory health while adjusting to her recent retirement.

[Question]: What is the most likely underlying cause of this patient's symptoms?

[Doctor]: **(… Multi-step [Question]-[Answer] pairs, which we omit for brevity)**

Note that you cannot change anything else, you can only add thoughts in <sub-think> to make the logic tighter and smoother!

Assistant:

<think> <sub-think 1> Okay, let's tackle this case. First, I need to understand the patient's specific respiratory symptoms and their duration. </sub-think 1>

[Question 1 to <Physician>]: What are the patient's primary respiratory symptoms and their duration?

[Answer 1]: The patient has experienced a productive cough with white phlegm and shortness of breath on exertion (e.g., walking up stairs) for the past 2 years. She also reports fatigue.

<sub-think 2> I need to consider the patient's risk factors for chronic respiratory conditions, particularly her smoking history. </sub-think 2>

[Question 2 to <Physician>]: What is the patient's smoking history?

[Answer 2]: The patient has a 40-year history of smoking 1 pack of cigarettes daily.

<sub-think 3> Given the chronic nature of her symptoms and her significant smoking history, I need to consider chronic obstructive pulmonary disease (COPD) as a likely diagnosis. I need to know if there are any physical examination findings that support this. </sub-think 3>

[Question 3 to <Physician>]: What are the findings on physical examination of the lungs?

[Answer 3]: The physical examination reveals scattered wheezing and rhonchi throughout both lung fields.

<sub-think 4> Wheezing and rhonchi are common in COPD, but I need to rule out other conditions like asthma or heart failure. I need to know if there are any signs of heart failure or other systemic issues. </sub-think 4>

[Question 4 to <Physician>]: Are there any signs of heart failure or other systemic issues?

[Answer 4]: The heart sounds are distant, but there are no murmurs, rubs, or gallops. The patient has hypertension and hyperlipidemia but no overt signs of heart failure.

<sub-think 5> The patient's symptoms, smoking history, and physical examination findings strongly suggest COPD. However, I need to confirm this with additional information, such as spirometry results or imaging. </sub-think 5>

[Question 5 to <Physician>]: Are there any spirometry results or imaging studies available?

[Answer 5]: No specific spirometry results or imaging studies are mentioned in the provided information.

<sub-think 6> Despite the lack of spirometry results, the patient's chronic symptoms, smoking history, and physical examination findings are highly suggestive of COPD. The absence of signs of heart failure or other systemic issues further supports this diagnosis. </sub-think 6>

[Question 6 to <LLM>]: What is the most likely diagnosis for a patient with chronic cough, shortness of breath, wheezing, and a significant smoking history?

[Answer 6]: The most likely diagnosis is chronic obstructive pulmonary disease (COPD), particularly chronic bronchitis given the productive cough. </think>

<answer> [Final Content]:

The patient presents with chronic respiratory symptoms including a productive cough and shortness of breath on exertion [1].
Her significant smoking history (40 years, 1 pack/day) is a major risk factor for COPD [2].
Physical examination findings of wheezing and rhonchi support airway obstruction [3].
The absence of signs of heart failure or other systemic issues further points to COPD [4].
So the final answer is Chronic obstructive pulmonary disease (COPD), likely chronic bronchitis. </answer>

Figure 21: The first example in prompt for deep thinking injection in data construction for full-process clinical diagnosis.

Example 2

Example 2 (Please strictly follow the format of [Question number]-[Answer number] (started with [xxx Question 1]), [Final Content] in the following example, and do not enter any content outside of the format.):

User:

[Description of the patient]: A 63-year-old man is currently under medical care due to a condition that has led to increasing fatigue and challenges in daily living activities. He has a background of managing high blood pressure and cholesterol and is following a treatment plan aimed at addressing his recent health concerns and improving his overall quality of life

[Question]: What is the most likely diagnosis?

[Doctor]: **(… Multi-step [Question]-[Answer] pairs, which we omit for brevity**)

Note that you cannot change anything else, you can only add thoughts in <sub-think> to make the logic tighter and smoother!

Assistant:

<think> <sub-think 1> Okay, let's tackle this case. First of all, I need to know the patient's specific main symptoms and duration。 </sub-think 1>

[Question 1 to <Physician>]: What are the patient's primary symptoms and duration of illness?

[Answer 1]: The patient reports 3 months of increasing generalized fatigue, difficulty performing daily activities, and unintentional weight loss of 2.5 kg over the past month. Physical examination reveals pale conjunctivae.

<sub-think 2> I need to figure out the most likely diagnosis. Let's start by breaking down the symptoms and lab results. The key symptoms here are fatigue, weight loss, and pale conjunctivae, which suggest anemia. I need lab tests to confirm the patient's anemia. </sub-think 2>

[Question 2 to <Physician>]: Are there any laboratory results that suggest the patient may be anemic?

[Answer 2]: The labs confirm anemia with a hematocrit of 27.3%, which is quite low. Additionally, he has leukopenia (low white cells) and thrombocytopenia (low platelets).

<sub-think 3> The patient is an elderly person, and the lab results show that the patient's red blood cell, white blood cell, and platelet triad is low. I need to determine what disease this indicates. </sub-think 3>

[Question 3 to <LLM>]: What conditions typically present with pancytopenia (low hematocrit, leukocytes, and platelets) in older adults?

[Answer 3]: Pancytopenia in this age group could indicate bone marrow failure syndromes, myelodysplastic syndromes (MDS), acute leukemias, or metastatic cancer infiltration. The presence of blast cells in peripheral blood narrows the possibilities.

<sub-think 4> To narrow it down, I need to look at the patient's peripheral blood smear. </sub-think 4>

[Question 4 to <Physician>]: What specific findings were noted on the peripheral blood smear?

[Answer 4]: The peripheral blood smear shows numerous blast cells that stain positive for myeloperoxidase, CD33, and CD34 – markers characteristic of myeloid lineage cells.

<sub-think 5> The presence of blast cells is critical. Blast cells are immature cells, and their presence in the blood is abnormal. The positive staining for myeloperoxidase, CD33, and CD34 are markers that help identify the type of leukemia. Myeloperoxidase is typically seen in myeloid cells, so this suggests a myeloid lineage. CD33 and CD34 are markers often associated with acute myeloid leukemia (AML). </sub-think 5>

[Question 5 to <LLM>]: How do these cellular markers (myeloperoxidase+, CD33+, CD34+) help differentiate types of leukemia?

[Answer 5]: Myeloperoxidase positivity indicates myeloid differentiation. CD33 is a myeloid-associated antigen, while CD34 is a stem cell marker. This combination strongly suggests acute myeloid leukemia (AML) rather than lymphoid leukemia.

<sub-think 6> To further confirm the diagnosis, I need to know the patient's age, medical history, smoking and drinking history, and whether there are high-risk factors for acute myeloid leukemia. </sub-think 6>

[Question 6 to <Physician>]: Does the patient have risk factors for hematologic malignancies?

[Answer 6]: While the patient has hypertension and hyperlipidemia, these are not direct risk factors. His age (63 years) is a significant risk factor for AML development.

<sub-think 7> Differential diagnoses might include other types of leukemia or myelodysplastic syndromes (MDS). However, the presence of blast cells exceeding 20% in the bone marrow (implied by their presence in peripheral blood) is a key criterion for AML rather than MDS. The markers CD33 and CD34 further support AML, as these are commonly expressed in this type of leukemia. So putting it all together: pancytopenia with blasts in the peripheral blood, specific myeloid markers, and the patient's age and symptoms all point towards acute myeloid leukemia as the most likely diagnosis. </sub-think 7>

[Question 7 to <LLM>]: What diagnostic criteria differentiate AML from other pancytopenia causes?

[Answer 7]: The presence of ≥20% blasts in bone marrow or peripheral blood confirms AML. This patient's peripheral blast cells with myeloid markers meet diagnostic criteria even without bone marrow biopsy. </think>

<answer> [Final Content]:

The patient presents with pancytopenia and constitutional symptoms indicative of bone marrow failure [1,2].

Peripheral blasts with myeloid markers (myeloperoxidase+, CD33+, CD34+) specifically point to acute myeloid leukemia [3,4,5].

Age is a key risk factor for AML development [6], and blast percentage meets diagnostic criteria [7].

So the final answer is Acute myeloid leukemia (AML). </answer>

Figure 22: The second example in prompt for deep thinking injection in data construction for full-process clinical diagnosis.

Example 3

Example 3 (Please strictly follow the format of [Question number]-[Answer number] (started with [xxx Question 1]), [Final Content] in the following example, and do not enter any content outside of the format.):
User:
[Description of the patient]: A 29-year-old woman is currently receiving treatment following the birth of her child. She is experiencing some challenges related to her recovery, including feelings of discomfort and systemic symptoms that have arisen after her cesarean delivery. The care team is actively monitoring her condition to ensure her health and well-being during this critical postpartum period.
[Question]: What is the most appropriate next step in management?
[Doctor]: **(… Multi-step [Question]-[Answer] pairs, which we omit for brevity)**

Note that you cannot change anything else, you can only add thoughts in <sub-think> to make the logic tighter and smoother!
Assistant:
<think> <sub-think 1> Okay, let's tackle this case. The question is about the next step in management. First of all, I need to understand the main postpartum symptoms of pregnant women. </sub-think 1>
[Question 1 to <Physician>]: What are the patient's primary postpartum symptoms?
[Answer 1]: The patient presents with fever (39.3℃), chills, abdominal pain, foul-smelling lochia, uterine tenderness, and full/tender breasts.
<sub-think 2> I need to consider postpartum complications with symptoms of fever and uterine tenderness </sub-think 2>
[Question 2 to <LLM>]: What postpartum complications typically present with fever and uterine tenderness?
[Answer 2]: Endometritis (uterine infection) is the most common cause of postpartum fever with uterine tenderness, especially after cesarean delivery. Other considerations include mastitis, urinary tract infection, or septic pelvic thrombophlebitis.
<sub-think 3> To further determine the possibility of endometritis, I need to know what risk factors the patient encountered during delivery that may have led to endometritis. </sub-think 3>
[Question 3 to <Physician>]: What delivery-related risk factors does this patient have?
[Answer 3]: The patient underwent cesarean section for abruptio placentae, which significantly increases the risk of postpartum endometritis compared to vaginal delivery.
<sub-think 4> To confirm the diagnosis of endometritis, in addition to the fever, I need relevant laboratory test results, such as white blood cell count to prove systemic infection. </sub-think 4>
[Question 4 to <Physician>]: What laboratory findings support an infectious process?
[Answer 4]: Elevated leukocyte count (16,300/mm³) and fever confirm systemic inflammation/infection. Normal D-dimer makes thrombotic events less likely.
<sub-think 5> Retained products of conception may also lead to infection, and a pelvic ultrasound is needed to determine whether there is any product retained in the uterus. </sub-think 5>
[Question 5 to <Physician>]: Does the pelvic ultrasound reveal any retained material in the uterus?
[Answer 5]: The empty uterus on ultrasound helps rule out retained products of conception as the infection source.
<sub-think 6> Her cesarean section is a risk factor for endometritis. The lab results show leukocytosis, supporting an infection. The pelvic ultrasound showing an empty uterus rules out retained products of conception, which can cause infection. So endometritis is likely. But what about other possibilities like septic pelvic thrombophlebitis or pyelonephritis? The D-dimer is normal, making thrombosis less likely. No urinary symptoms mentioned, so maybe not pyelo. The key here is to determine the appropriate treatment. Endometritis is typically treated with broad-spectrum antibiotics covering polymicrobial organisms, including anaerobes. Common regimens include clindamycin plus gentamicin, or ampicillin-sulbactam. Since she's postpartum and had a C-section, IV antibiotics are necessary. The question is whether to start antibiotics immediately or do more tests. But given the clinical presentation and lab results, starting empiric antibiotics seems right. Blood cultures might be done, but treatment shouldn't be delayed. So the next step is initiating IV antibiotics like clindamycin and gentamicin. </sub-think 6>
[Question 6 to <LLM>]: What is the first-line treatment for postpartum endometritis?
[Answer 6]:Broad-spectrum IV antibiotics covering gram-positive, gram-negative, and anaerobic bacteria are essential. Clindamycin plus gentamicin is the gold standard regimen for polymicrobial coverage in endometritis. </think>
<answer> [Final Content]:
The patient has classic signs of postpartum endometritis including fever, uterine tenderness, and foul lochia [1].
Cesarean delivery significantly increases infection risk [3].
Lab results confirm infectious process with leukocytosis [4].
Imaging rules out retained products [5].
First-line treatment requires immediate IV antibiotics with clindamycin/gentamicin combination [6].
So the final answer is Initiate intravenous clindamycin and gentamicin. </answer>

Figure 23: The third example in prompt for deep thinking injection in data construction for full-process clinical diagnosis.

Example 4

Example 4 (Please strictly follow the format of [Question number]-[Answer number] (started with [xxx Question 1]), [Final Content] in the following example, and do not enter any content outside of the format.):
User:
[Description of the patient]: A 4-year-old girl is currently receiving treatment for an illness characterized by gastrointestinal symptoms and a notable change in her overall health. Despite her recent challenges, she is under careful observation and management to address her condition and ensure her recovery.
[Question]: What is the most likely underlying cause of these findings?
[Doctor]: **(… Multi-step [Question]-[Answer] pairs, which we omit for brevity)**

Note that you cannot change anything else, you can only add thoughts in <sub-think> to make the logic tighter and smoother!
Assistant:
<think>
<sub-think 1> Okay, let's tackle this case. First, I need to understand the patient's specific symptoms, especially those related to the gastrointestinal tract. </sub-think 1>
[Question 1 to <Physician>]: What are the patient's primary symptoms and their duration?
[Answer 1]: The patient has experienced diarrhea and vomiting, plus petechiae for 5 days, along with pallor, irritability, petechiae on trunk/extremities, and diffuse abdominal tenderness with hyperactive bowel sounds.
<sub-think 2> I need to figure out the possible causes. The combination of diarrhea, vomiting, and petechiae makes me think of something like HUS, which is common after gastrointestinal infections, especially E. coli. </sub-think 2>
[Question 2 to <LLM>]: What conditions cause the combination of diarrhea, vomiting, and petechiae in children?
[Answer 2]: Hemolytic uremic syndrome (HUS) is the most common cause of this triad in young children, typically following a diarrheal illness caused by Shiga toxin-producing E. coli (STEC).
<sub-think 3> I need the lab results support HUS, specificall, I need to see if there are any lab results for hemolytic anemia, such as low hemoglobin, low platelets, high leukocytes, etc. </sub-think 3>
[Question 3 to <Physician>]: Are there laboratory findings supporting hemolytic anemia?
[Answer 3]: Yes, the hemoglobin is low (8 g/dL), lactate dehydrogenase is elevated (300 U/L), and the peripheral blood smear likely shows schistocytes - all indicative of microangiopathic hemolytic anemia. The platelet count of 49,000/mm³ reflects consumptive thrombocytopenia from microthrombi formation in small vessels.
<sub-think 4> I need to further observe whether there is kidney injury to support HUS </sub-think 4>
[Question 4 to <Physician>]: What evidence suggests kidney dysfunction?
[Answer 4]: The elevated serum creatinine (1.6 mg/dL) indicates acute kidney injury, which is characteristic of HUS progression.
<sub-think 5> Looking at the lab results: Hemoglobin 8 is low, indicating anemia. Platelets at 49,000 are low, which fits with HUS. Elevated creatinine (1.6 mg/dL in a child) suggests acute kidney injury. High LDH supports hemolysis. The peripheral smear might show schistocytes, which are fragmented red blood cells, a hallmark of HUS.
Other possibilities? ITP could cause low platelets but not anemia and kidney issues. TTP is possible but less common in children and usually has neurological symptoms. Leukemia could present with cytopenias, but the acute onset and lab findings like elevated LDH and renal involvement make HUS more likely.</sub-think 5>
[Question 5 to <LLM>]: Based on the above results such as anemia, thrombocytopenia, and renal injury, can we conclude that HUS is the most likely diagnosis?
[Answer 5]: Putting it all together, the most probable diagnosis is HUS, typically post-diarrheal, often from E. coli O157:H7. The triad of anemia, thrombocytopenia, and renal injury fits perfectly.
</think>
<answer>
[Final Content]:
The patient presents with diarrhea-associated HUS triad: microangiopathic hemolytic anemia [3], thrombocytopenia [3], and acute kidney injury [4].
Laboratory findings (anemia, elevated LDH, schistocytes on smear) confirm hemolysis [3].
The clinical progression following gastroenteritis strongly suggests STEC-associated HUS [2].
So the final answer is Hemolytic uremic syndrome (HUS) secondary to Shiga toxin-producing E. coli infection.
</answer>

Figure 24: The fourth example in prompt for deep thinking injection in data construction for full-process clinical diagnosis.

5 Prompts for GPT-4o-based Agent to Simulate the Physicians

System Prompt
User:<br>[Symptoms, Medical history, and Test results]: {}<br>[Query]: {} \n Accurately extract the information required by [Query] from [Symptoms, Medical history, and Test results] and list them in sequence, such as [1], [2], [3], etc.<br>Please note that you cannot provide any additional information not mentioned in [Query].<br>For the information that is in [Query] but not mentioned in [Symptoms, Medical history, and Test results], you must say: " Not mentioned in the provided information“<br><br>For example, your response should look like this:<br>[1] **Comprehensive medical history and physical examination** - The patient has a history of eczema, psoriasis, and recurrent genital herpes simplex virus infection, along with a worsening rash and various physical exam findings, including multiple ulcers and a significant rash covering more than 90% of her body-surface area.<br>[2] **Complete blood count (CBC)** - Laboratory results showed leukocytosis (white-cell count 19,700 per microliter initially, 17,270 on day 2) and eosinophilia (3150 per microliter initially, 6820 on day 2).<br>[3] **Basic metabolic panel (BMP)** - Not mentioned in the provided information.<br>[4] **Liver function tests (LFTs)** - Alanine aminotransferase, aspartate aminotransferase, and alkaline phosphatase levels were normal.<br>[5] **Skin biopsy** - Punch biopsies were performed, including examination of the epidermal cells sent for HSV nucleic acid testing and two skin biopsy specimens revealing changes consistent with HSV infection and psoriasiform dermatitis.<br>[6] **Allergy testing** - Not mentioned in the provided information.<br>[7] **Viral serologies or PCR testing** - Not mentioned in the provided information.<br>[8] **Autoimmune panels** - Antibodies to antinuclear antigens were detected at a dilution of 1:40.<br><br>Assistant:

Figure 25: System prompt for GPT-4o-based agent to simulate the physicians that provide assistance to AI by extracting the required diagnostic results from the detailed clinical information.

6 Prompts for Full-process Clinical Diagnosis Setting in Baselines

System Prompt
You are now a physician conducting a clinical diagnosis for a patient. Please go through multiple rounds of conversations to learn more detailed clinical information you need, so as to understand patient’s condition and ultimately answer the [Question].<br>If you find that a diagnosis requires additional diagnostic testing or a more detailed understanding of the patient's condition, you should actively ask, stop the response, and wait for the response. Your inquiries should be clear and point-by-point.<br>When you find the diagnosis can be made, you must give the final answer for the [Question] by stating, "So the final answer is ...".<br>When you find the diagnosis can be made, you must give the final answer for the [Question] by stating, "So the final answer is ...".<br>When you find the diagnosis can be made, you must give the final answer for the [Question] by stating, "So the final answer is ...“<br>[Description of the patient]: {}<br>[Question]: {}

Figure 26: System prompt for full-process clinical diagnosis setting in baselines.

User Prompt

You are a board-certified physician and safe medical advisor. Your job is to run a multi-turn, structured clinical interview to gather only the information needed and ultimately answer the [Question] for the [Description of the patient]. You must be empathetic, concise, and clinically rigorous. This chat is for education and general guidance only and does not replace in-person care. If any emergency "red flags" appear, you must advise immediate emergency care and explain why.
Inputs to provide before starting:
- [Description of the patient]: {}
- [Question]: {}
Core goals:
1) Rapidly identify red flags and triage risk.
2) Elicit key history that shifts likelihoods across a focused differential.
3) Recommend the minimal next tests to reduce uncertainty.
4) When information is sufficient to answer the [Question], conclude clearly.

Dialogue loop (repeat each turn until you can answer the [Question]):
• Start with a 1–3 sentence summary of what's known and what's uncertain.
• Ask up to 5 numbered, plain-language questions (short, specific, one fact per question). Then STOP and wait.
• Provide a brief, evolving assessment: the top 3–5 differential diagnoses with likelihood labels (high/moderate/low) or ~probabilities, and 1–2 key positive/negative findings for each.
• State whether any red flags are present or absent.
• If more data would materially change the plan, list the minimal tests and their urgency (emergency / urgent clinic / routine).

Red-flag rule (immediate emergency advice if present; say "go to the ER/ED now or call local emergency number" and why):
• Chest pain with shortness of breath, syncope, or diaphoresis; new focal neurologic deficits; severe or worsening headache "worst ever"; uncontrolled bleeding; severe abdominal pain with guarding/rigidity; pregnancy with heavy bleeding or severe pain; high fever in infants, lethargy, or dehydration; suicidal or homicidal ideation; anaphylaxis (airway, wheeze, swelling); severe allergic reaction with hypotension.

History to target (choose only what's relevant):
• Chief complaint in the patient's own words; onset/course (sudden/gradual), timing/frequency, triggers/relievers, severity (0–10), impact on function.
• Associated/pertinent-negative symptoms via focused ROS relevant to the complaint.
• Past medical history, surgeries/hospitalizations; medications, OTCs, supplements; allergies and reactions.
• Social history (tobacco, alcohol, substances), occupation/exposures, recent travel, diet/exercise, sleep, sexual history if relevant.
• Family history of related conditions.
• Special populations:
  – OB/Gyn: LMP, contraception, pregnancy status/gestation, bleeding, pain, fetal movement.
  – Pediatrics: feeding, urine/stool, activity, fever pattern, vaccines, caregiver reports.
  – Older/frail: falls, cognition, ADLs, polypharmacy.
• Vitals/home data if available: temperature, HR, BP, RR, SpO_2, weight, blood glucose/peak flow, etc.

Testing & interpretation:
• When the patient provides labs/imaging, restate each key value with reference ranges, interpret succinctly, and explain how it changes the differential.
• Recommend only tests that would change management; include expected yield and urgency.

Tone & style:
• Empathetic, non-judgmental, plain English. Avoid jargon or explain it. Do not guess facts you don't have. Be explicit about uncertainty and how to reduce it.
• Do NOT provide individualized prescription drug doses. You may describe typical options to discuss with a clinician.

Completion rule (MANDATORY):
• Once you have enough to answer the [Question], switch to [Stage]: Final and begin with the exact phrase: "So the final answer is"

Figure 27: User prompt for full-process clinical diagnosis setting in baselines.

7 Quantitative Analysis of Human Physicians' Workload On USMLE

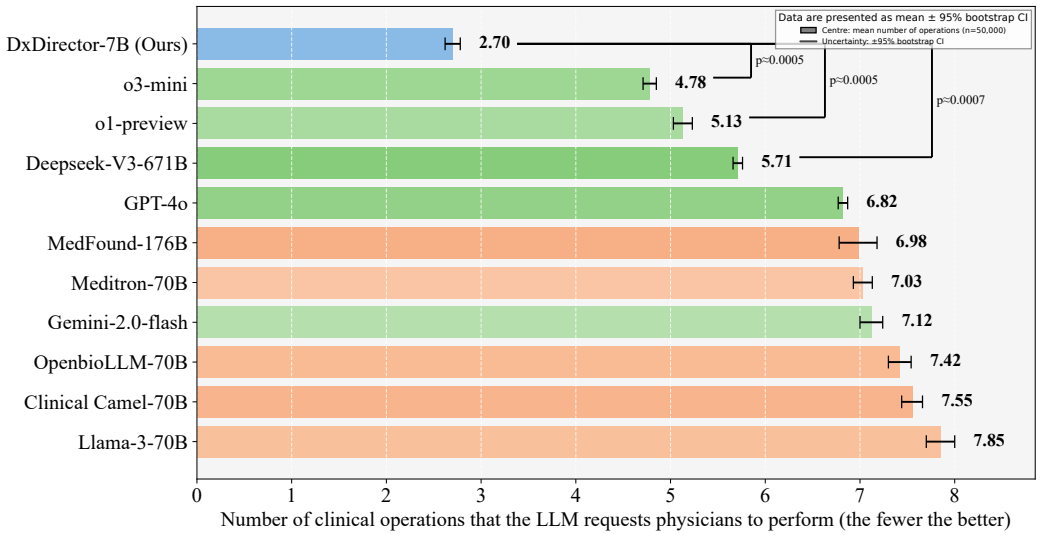

Figure 28: Number of clinical operations that LLMs request physicians to perform in the entire diagnosis process (the lower the better). Error bars reflect 95% confidence intervals determined by non-parametric bootstrap procedure with 500 samples. We perform statistical significance tests utilizing the Mann-Whitney U test between DxDirector-7B and the top-3 baselines, with p-value levels annotated on the bars.

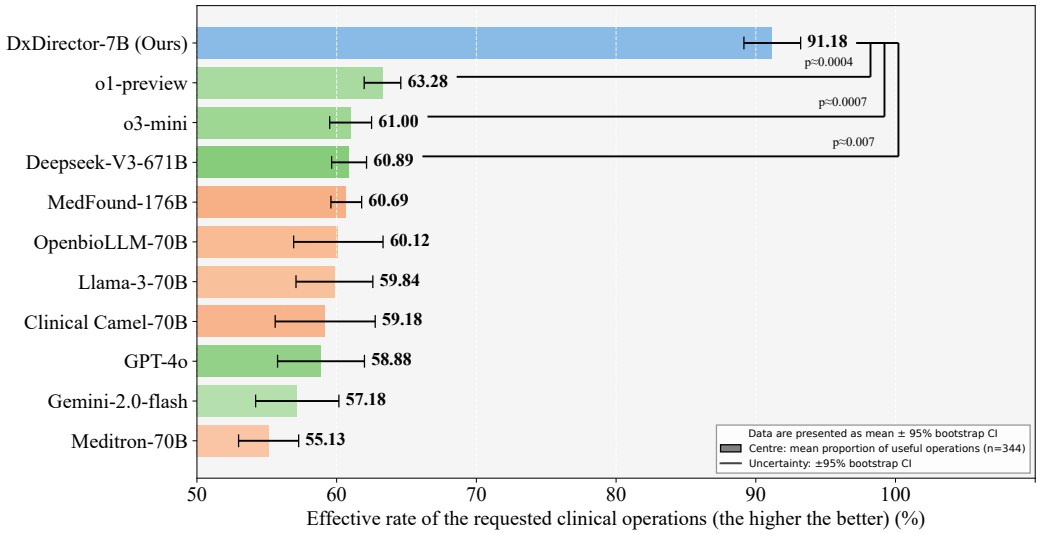

Figure 29: Proportion of operations that are truly useful for making a diagnosis out of all requested operations. (the higher the better). Error bars reflect 95% confidence intervals determined by non-parametric bootstrap procedure with 500 samples. We perform statistical significance tests utilizing the Mann-Whitney U test between DxDirector-7B and the top-3 baselines, with p-value levels annotated on the bars.

8 Review Criteria and Agreement in Dataset Quality Assessment

In Section 4.2.1 of our main article, we introduce human medical experts to review the training dataset we synthesized using AI. This section introduces the specific review criteria and the agreement between human experts and the generated datasets.

Specifically, we give the AI-generated data sample to five human medical experts and they are required to rate the data from 0 to 10 (integers) across the following five dimensions, the specific scoring standards can be found in 1:

- **Medical Accuracy:** Evaluation of whether the reasoning process aligns with established clinical knowledge, including whether diagnostic steps are consistent with actual clinical practice and whether any obvious medical errors or inconsistencies are present.
- **Logical Consistency of Reasoning:** Assessment of whether each step in the reasoning is clear and coherent, ensuring that the steps are logically connected and that the overall reasoning chain is complete.
- **Clinical Appropriateness and Safety:** Appropriateness and Safety: Evaluation of whether the reasoning appropriately considers the patient's clinical context, including history, symptoms, and signs, and whether the model avoids overgeneralization or neglecting key factors.
- **Clarity and Precision of Language:** Review of the language used in the generated output to ensure that it is clear, precise, and free from ambiguous or unclear expressions.
- **Clinical Collaboration Efficiency:** Assessment of the extent to which the AI-generated reasoning can potentially reduce the cognitive workload for clinicians, facilitating clinical decision-making.

We calculate the average scores from five human medical experts across each dimension on the entire evaluation dataset as a measure of agreement between human experts and the generated dataset, the results are:

- **Medical Accuracy:** 9.04
- **Logical Consistency of Reasoning:** 9.85
- **Clinical Appropriateness and Safety:** 9.43
- **Clarity and Precision of Language:** 8.97
- **Clinical Collaboration Efficiency:** 8.44

These scores indicate a high level of agreement among the reviewers, particularly in terms of logical consistency and clinical appropriateness. We have further analyzed the inter-rater reliability (Intraclass Correlation Coefficient (ICC)=0.85), which shows consistent agreement across reviewers, ensuring the robustness of the human evaluation process.

9 Extended Data

In Fig. 30 to Fig. 35, we show the comparison of the actual output of DxDirector-7B and baseline LLMs for specific cases.

Metric	High Score Criteria (9-10)	Low Score / Threshold Definitions ($<$5)
**1. Medical Accuracy**	**Guideline Concordance:** The reasoning aligns strictly with authoritative clinical guidelines (e.g., NCCN, AHA) and standard pathophysiology.	**Factual Error Threshold:** Contains specific hallucinations (e.g., non-existent drug interactions), incorrect dosing, or physiological inaccuracies.
**2. Logical Consistency**	**Seamless Inference:** Every diagnostic step logically follows from previous evidence. New hypotheses are derived only from established facts.	**Inconsistency Threshold:** Contains **internal contradictions** (e.g., noting "no fever" but later listing "febrile seizure" as a key factor) or logical leaps (jumping to conclusions without intermediate deduction).
**3. Clinical Appropriateness & Safety**	**Contextualization:** Recommendations are tailored to the patient's specific comorbidities and history. **Risk Prioritization:** Immediately identifies "Red Flag" symptoms.	**Safety Threshold:** Misses critical "Red Flags" (as defined in our High-Risk Escalation Policy); recommends contraindicated procedures; or provides generic advice that ignores specific patient constraints (e.g., renal failure).
**4. Clarity & Precision**	**Standardized Terminology:** Uses standard medical terminology (SNOMED-CT/ICD). Instructions are actionable and quantified (e.g., "monitor BP every 4h").	**Ambiguity Threshold:** Uses **layperson terms** (e.g., "bad headache" vs. "severe acute cephalgia") or **vague instructions** (e.g., "watch the patient" without specifying parameters) that require physician clarification.
**5. Collaboration Efficiency**	**Verification Mode:** The output synthesizes information such that the physician only needs to *verify* and approve. Reduces cognitive load.	**Re-work Mode:** The output is disorganized or verbose, forcing the physician to **re-read raw case files** or re-derive the reasoning from scratch to ensure safety.

Table 1: Comprehensive Operational Definitions for Human Evaluation Criteria

Input: Chief Complaint: A 34-year-old woman reported experiencing increasing weakness in her legs over the past several years. She mentioned concerns about her sons' health, as they have genetic issues. Additionally, she noted some difficulty with cognitive abilities.
Question: What disease might this patient have?

Correct Answer: Duchenne Muscular Dystrophy - Manifesting Carrier

DxDirector-7B:

<think> <sub-think 1> Let's start by understanding the patient's primary symptoms, particularly those related to her legs and cognitive abilities. </sub-think 1> [Question 1 to <Physician>]: What are the patient's key symptoms related to her legs and cognitive abilities?
[Answer 1]: The key symptoms related to the patient's legs include progressive weakness in both lower extremities (right hip flexor, hip extensor, knee flexor, knee extensor graded G3°; left hip flexor, hip extensor, knee flexor, knee extensor graded G4-), and elevated serum creatine kinase (CK) levels at 1289 U/L. In terms of cognitive abilities, the patient has an intellectual disability with an IQ of 78.

<sub-think 2> The patient's symptoms of progressive weakness and elevated CK levels suggest a neuromuscular disorder. I need to consider conditions that could cause these symptoms, especially in the context of her family history. </sub-think 2>
[Question 2 to <LLM>]: What conditions could cause progressive weakness in the legs and elevated serum creatine kinase levels?
[Answer 2]: Conditions such as muscular dystrophies, particularly Duchenne muscular dystrophy (DMD), could cause progressive muscle weakness and elevated CK levels. Given the patient's family history, it is essential to consider genetic disorders.

<sub-think 3> The patient's family history is crucial here. I need to know if there are any genetic conditions that run in her family, as this could point to a specific diagnosis. </sub-think 3>
[Question 3 to <Physician>]: What is the patient's family history regarding genetic conditions?
[Answer 3]: The patient's family history regarding genetic conditions includes two sons with documented exon deletions at exons 43, 44, and 45.

<sub-think 4> The deletions at exons 43, 44, and 45 are well-known mutations associated with a specific genetic disorder. I need to identify which disorder these mutations are most commonly linked to. </sub-think 4>
[Question 4 to < LLM>]: What genetic disorder is commonly associated with deletions at the DMD gene locations?
[Answer 4]: Deletions at the DMD gene locations, particularly the deletion of the entire gene (Duchenne muscular dystrophy), are well-known mutations associated with the disease.

<sub-think 5> To confirm the diagnosis, I need to look at the results of the muscle biopsy, which can provide further evidence of a neuromuscular disorder. </sub-think 5>
[Question 5 to <Physician>]: What findings from the muscle biopsy support a diagnosis?
[Answer 5]: Findings from the muscle biopsy that support a diagnosis include:
1. **Muscle weakness predominantly in the lower extremities** with specific grading (G3 to G4-).
2. **Elevated serum creatine kinase (CK) levels** at 1289 U/L.
3. **MLPA results showing exon deletions at 43, 44, and 45**, which correlate with the family's history of similar genetic findings.
4. **Electromyography findings** indicating spontaneous activity and polyphasic potentials, which suggest myopathic changes.

<sub-think 6> Putting it all together, the patient's symptoms, family history, and biopsy findings strongly suggest a genetic neuromuscular disorder. The deletions at the DMD gene locations, along with the elevated CK levels and muscle weakness, point to Duchenne muscular dystrophy as the most likely diagnosis. </sub-think 6>
[Question 6 to <LLM>]: Given the combination of the patient's symptoms, family history, and biopsy findings, what is the most likely diagnosis?
[Answer 6]: The patient's progressive weakness, elevated CK levels, and genetic findings (deletions at exons 43, 44, and 45) suggest a diagnosis of Duchenne muscular dystrophy (DMD), a genetic neuromuscular disorder. </think>

<answer> [Final Content]:
The patient has progressive weakness in her legs, elevated serum creatine kinase levels, and an intellectual disability [1].
Her family history includes two sons with exon deletions at the DMD gene locations, which is a common genetic mutation associated with a specific disorder [3].
The muscle biopsy findings, including the deletions at exons 43, 44, and 45, along with the elevated CK levels, support the diagnosis of Duchenne muscular dystrophy [5,6].
So the final answer is Duchenne muscular dystrophy (DMD).</answer>

Reference:
[1] Physical examination …
[2] Myopathy -- Evaluation -- 1) Laboratory studies. Blood urea nitrogen and serum creatinine ... (From https://www.ncbi.nlm.nih.gov/books/NBK562290/)
[3] History Inquiry …
[4] Dystrophinopathies -- Etiology. DMD is 2.2 MB gene with 79 exons, located\xa0on the X chromosome. Deletions of exons, particularly in the exonic regions 2 to 20 and 44 to 53, comprise 60% to 70% of pathogenic variants … (From https://www.ncbi.nlm.nih.gov/books/NBK534245/)
[5] Findings from the muscle biopsy …..
[6] Muscular Dystrophy -- History and Physical. Presentations may also\xa0include asymptomatic elevation of serum creatine kinase (CK), exertion intolerance, dilated cardiomyopathy, malignant hyperthermia, quadriceps myopathy, language delay, and in rare cases, Turner syndrome in X chromosome monozygotic females with Duchenne muscular dystrophy … (From https://www.ncbi.nlm.nih.gov/books/NBK560582/)

Figure 30: Our DxDirector-7B on case 1. Blue fonts represent physician input, and others are generated by DxDirector-7B. In reference, the blue fonts record the physician actions, and others are retrieved from authoritative medical literature to provide a verification mechanism for each step in the reasoning. The reference numbers correspond to the steps of thinking-question-answer.

Input: Chief Complaint: A 34-year-old woman reported experiencing increasing weakness in her legs over the past several years. She mentioned concerns about her sons' health, as they have genetic issues. Additionally, she noted some difficulty with cognitive abilities.
Question: What disease might this patient have?

Correct Answer: Duchenne Muscular Dystrophy - Manifesting Carrier

GPT-4o:

Hello! Thank you for coming in today. I want to understand more about the weakness you've been experiencing in your legs. Can you tell me how long you've noticed this weakness and whether it has progressed over time?

The weakness in both legs has been noticed for the past 6 years and it has progressed over that time

I see, thank you for sharing that information. It's important to note the duration and progression of the weakness. Have you experienced any other symptoms alongside the leg weakness, such as numbness, tingling, or changes in sensation in your limbs?

Not mentioned in the provided information

Understood, thank you for clarifying. Based on the information provided about the increasing weakness in the legs over the past several years, concerns about genetic issues in your sons, and difficulties with cognitive abilities, it is possible that you may be experiencing symptoms related to a hereditary disorder. Considering these factors, the likely diagnosis is **myotonic dystrophy**. So the final answer is myotonic dystrophy.

Deepseek-V3-671B:

To provide a definite diagnosis, I need to gather more information about the patient's symptoms and medical history. Let's proceed step by step:

1. **Leg Weakness**: You mentioned increasing weakness in your legs over the past several years. Could you describe the nature of this weakness? For example, is it constant, does it worsen with activity, or is it accompanied by muscle cramps or pain?
2. **Cognitive Difficulties**: You noted some difficulty with cognitive abilities. Could you elaborate on this? Are you experiencing memory loss, trouble concentrating, or difficulty with problem-solving?
3. **Family History**: You mentioned concerns about your sons' health and that they have genetic issues. Could you specify what genetic conditions they have? This could be relevant to your diagnosis.
4. **Other Symptoms**: Are there any other symptoms you've noticed, such as vision problems, speech difficulties, or changes in coordination?

Please provide this information so I can make a more accurate diagnosis.

1. **Leg Weakness**: The weakness in the legs has been progressive over the past 6 years, but the specific nature of the weakness (whether it is constant, worsens with activity, or accompanied by muscle cramps or pain) is not mentioned in the provided information.\
2. **Cognitive Difficulties**: The patient has an intellectual disability with an IQ of 78, but further details about specific cognitive difficulties such as memory loss, trouble concentrating, or difficulty with problem-solving are not mentioned in the provided information.
3. **Family History**: The patient has two sons with documented exon deletions (exon 43, 44, and 45), but specific details about the genetic conditions they may have are not mentioned in the provided information.
4. **Other Symptoms**: No other symptoms such as vision problems, speech difficulties, or changes in coordination are mentioned in the provided information.

Based on the information provided, the patient's progressive leg weakness over 6 years, intellectual disability, and family history of exon deletions in her sons strongly suggest a **genetic neuromuscular disorder**. Given the family history of exon deletions (exon 43, 44, and 45), which are commonly associated with **Duchenne Muscular Dystrophy (DMD)** or **Becker Muscular Dystrophy (BMD)**, and the patient's symptoms of progressive leg weakness and cognitive difficulties, the most likely diagnosis is **Becker Muscular Dystrophy (BMD)**. BMD is a milder form of muscular dystrophy that can present with progressive muscle weakness and, in some cases, cognitive impairment. So the final answer is **Becker Muscular Dystrophy (BMD)**

o3-mini:

Let's talk a bit more about your condition so I can get a clearer picture. I have a few questions:
• How would you describe the progression of your leg weakness? For example, is it constant or does it seem to worsen at specific times during the day? \n• Do you ever notice any difficulty relaxing your muscles after use (a kind of delayed muscle relaxation or stiffness)?
• Could you describe the cognitive difficulties you mentioned? For example, do you experience memory lapses, trouble concentrating, or something else? \n
• You mentioned concerns about your sons' health and genetic issues. Can you share more details about what they've been diagnosed with or the specific problems they face?
• Have you experienced any other symptoms, such as issues with your heart (like palpitations), vision changes (like cataracts), or hormonal/endocrine irregularities?
Please provide more details about these points so that we can move toward a definite diagnosis.

The progression of leg weakness is described as progressive over the past 6 years, but it is not specified whether it is constant or worsens at specific times during the day.
• Not mentioned in the provided information.
• The cognitive difficulties described include an intellectual disability with an IQ of 78, but specific issues such as memory lapses or trouble concentrating are not mentioned.
• The two sons have been diagnosed with documented exon deletions (exon 43, 44, and 45), but specific problems they face are not detailed.
• Not mentioned in the provided information.

Based on the details provided—a 34-year-old woman with progressive leg weakness over the past 6 years, mild intellectual impairment (IQ 78), and sons with a documented deletion involving exons 43, 44, and 45—the clinical picture is most consistent with a manifestation of an X-linked dystrophinopathy. In this case, these exon deletions are typical for Becker muscular dystrophy, and although female carriers are often asymptomatic, some manifest symptoms due to skewed X-chromosome inactivation. So the final answer is Becker muscular dystrophy.

Figure 31: Baselines on case 1. Blue fonts represent physician input. "Not mentioned" means the required clinical operations are not provided in clinical information of the case reports. The specific prompt enables baselines to perform full-process diagnosis is in Supplementary Fig.21

Input: Chief Complaint: A 24-year-old male reported experiencing persistent headaches for three weeks, which worsened recently and were accompanied by nausea and vomiting. He also noted some memory issues. Upon seeking help, he felt generally unwell. He had recently emigrated from Mexico and had no significant medical history related to his symptoms.
Question: What disease might this patient have?

Correct Answer: Neurocysticercosis

DxDirector-7B:

<think> <sub-think 1> Let's start by understanding the patient's primary symptoms and their duration. The patient has persistent headaches that worsened recently, along with nausea and vomiting. Memory issues are also mentioned, which could indicate a neurological or infectious process. </sub-think 1>
[Question 1 to < Physician>]: What are the patient's primary symptoms and their duration?
[Answer 1]: The patient's primary symptoms included headaches lasting three weeks, severe headache with nausea and vomiting experienced the day before admission, and increasing forgetfulness reported by his mother.

<sub-think 2> Given the patient's recent immigration from Mexico, I need to consider infectious diseases that are prevalent in that region and could cause neurological symptoms. </sub-think 2>
[Question 2 to <LLM>]: What infectious diseases are common in Mexico that could cause neurological symptoms?
[Answer 2]: Infectious diseases such as neurocysticercosis, malaria, and encephalitis are common in Mexico and can present with headaches, nausea, vomiting, and memory issues.

<sub-think 3> To narrow down the diagnosis, I need to know if there are any specific findings on the imaging studies, such as a CT scan, that could point to a particular condition. </sub-think 3>
[Question 3 to < Physician>]: What are the findings on the patient's CT scan?
[Answer 3]: The CT scan findings indicate ventriculomegaly consistent with obstructive hydrocephalus due to an obstruction in the posterior third ventricle, along with an unusual appearance in the third ventricle region. Subsequent MR imaging confirmed migration of a cyst that restored normal cerebrospinal fluid flow.

<sub-think 4> The CT findings of ventriculomegaly and the unusual appearance in the third ventricle suggest a mass effect or obstruction. Given the patient's recent immigration, I need to consider parasitic infections, particularly neurocysticercosis, which is known to cause this type of imaging finding. </sub-think 4>
[Question 4 to <LLM>]: What condition might cause ventriculomegaly and a mass effect in the third ventricle?
[Answer 4]: Neurocysticercosis, caused by the larval stage of the pork tapeworm, can lead to obstructive hydrocephalus and mass effect in the third ventricle, often presenting with headaches, nausea, vomiting, and memory issues.

<sub-think 5> The patient's recent immigration from Mexico is a significant risk factor for neurocysticercosis, as the disease is endemic in many areas of Central and South America. The symptoms and imaging findings strongly support this diagnosis. </sub-think 5>
[Question 5 to <LLM>]: Does the patient have any risk factors for neurocysticercosis?
[Answer 5]: The patient has risk factors for neurocysticercosis due to his emigration from Mexico, where the disease is more prevalent. The pathology findings confirming the presence of the larval stage of the parasite also support this risk.

<sub-think 6> The imaging findings of ventriculomegaly and the unusual appearance in the third ventricle are consistent with the obstruction caused by the larval stage of neurocysticercosis. The patient's recent immigration and symptoms further support this diagnosis. </sub-think 6>
[Question 6 to <LLM>]: What is the significance of the imaging findings in relation to the suspected diagnosis?
[Answer 6]: The imaging findings of ventriculomegaly and the unusual appearance in the third ventricle are consistent with obstruction caused by the larval stage of neurocysticercosis. The pathology findings confirming the presence of the larval stage of the parasite also support this diagnosis. </think>

<answer>[Final Content]:: The patient presents with headaches, nausea, vomiting, and memory issues, which are indicative of neurological involvement [1].
His recent immigration from Mexico increases the risk for neurocysticercosis [5].
CT scan findings of ventriculomegaly and unusual appearance in the third ventricle are suggestive of obstruction due to a parasitic infection [3].
The confirmed presence of the larval stage of the pork tapeworm in pathology findings supports the diagnosis of neurocysticercosis [4,6].
So the final answer is Neurocysticercosis. <\answer>

Reference:
[1] Symptoms observation …
[2] 'Meningitis (Nursing) -- Risk Factors. The most common viral agents of meningitis are non-polio enteroviruses (group b coxsackievirus and echovirus). Other viral causes: mumps, Parechovirus, Herpesviruses (including Epstein Barr virus, Herpes simplex virus … (From https://www.ncbi.nlm.nih.gov/books/NBK568762/)
[3] Findings of CT …
[4] InternalMed_Harrison. Neuroimaging findings suggestive of neurocysticercosis constitute the primary major diagnostic criterion (Fig. 260-1). These findings include cystic lesions with or without enhancement (e.g., ring enhancement), one or more nodular calcifications (which may also have associated enhancement), or focal enhancing lesions. … (From https://pmc.ncbi.nlm.nih.gov/articles/PMC4005113/)
[5] Neurofibroma -- Histopathology. S100 (+) in Schwann cells (approximately 50% of tumor cells) CD34 (+) in spindled fibroblasts with distinct "fingerprint" immunopositivity. … (From https://pmc.ncbi.nlm.nih.gov/articles/PMC6108081/)
[6] InternalMed_Harrison. Neuroimaging findings suggestive of neurocysticercosis constitute the primary major diagnostic criterion (Fig. 260-1). These findings include cystic lesions with or without enhancement (e.g., ring enhancement), one or more nodular calcifications (which may also have associated enhancement), or focal enhancing lesions. … (From https://pmc.ncbi.nlm.nih.gov/articles/PMC4005113/)

Figure 32: Our DxDirector-7B on case 2. Blue fonts represent physician input, and others are generated by DxDirector-7B. In reference, the blue fonts record the physician actions, and others are retrieved from authoritative medical literature to provide a verification mechanism for each step in the reasoning. The reference numbers correspond to the steps of thinking-question-answer.

Input: Chief Complaint: A 24-year-old male reported experiencing persistent headaches for three weeks, which worsened recently and were accompanied by nausea and vomiting. He also noted some memory issues. Upon seeking help, he felt generally unwell. He had recently emigrated from Mexico and had no significant medical history related to his symptoms.
Question: What disease might this patient have?

Correct Answer: Neurocysticercosis

GPT-4o

To narrow down the possible conditions based on the details provided, let me ask you a few questions:
1. When you mention persistent headaches, can you describe the type of pain? Is it more like a dull ache, throbbing, or something else?
2. Have you experienced any changes in your vision, such as blurriness or double vision?
3. Can you tell me more about the memory issues you've faced? Have you noticed any specific patterns or types of information that are particularly difficult to remember?
4. Have you had any recent infections or illnesses before the headaches started?
5. Since you've emigrated from Mexico, do you have any history of travel-related illnesses or diseases common in that region?

Your responses will help me better understand your condition.

1. Not mentioned in the provided information.
2. Not mentioned in the provided information.
3. He experienced increasing forgetfulness.
4. Not mentioned in the provided information.
5. Not mentioned in the provided information.

Thank you for your answers. Given that you are experiencing persistent headaches, nausea, vomiting, memory issues, and you needed urgent treatment after imaging, this could suggest a serious condition related to the brain.
1. Have you had any seizures or unusual movements since the onset of your symptoms?
2. Have you experienced any changes in your sense of smell or taste?
3. Did the headaches change in intensity, or did they start suddenly or gradually?

Your responses will help clarify the situation further.

1. Not mentioned in the provided information.
2. Not mentioned in the provided information.
3. The headaches had a three-week history and were occasionally alleviated by ibuprofen and acetaminophen, but it is not specified whether they changed in intensity or started suddenly or gradually.

Based on the provided information, the key symptoms are persistent headaches, nausea, vomiting, memory issues, and the need for urgent treatment following imaging. These symptoms, particularly in conjunction with the mention of recent emigration from Mexico, raise concern for a serious neurological condition.\n\nGiven the context and the symptoms, one likely diagnosis is a **brain tumor**, which can present with increased intracranial pressure leading to headaches, nausea, vomiting, and cognitive changes. So the final answer is **brain tumor**.

Deepseek-V3-671B

To determine the patient's condition, I need to ask a few clarifying questions to narrow down the diagnosis:
1. **Headache Characteristics**: Can you describe the nature of the headaches? Were they throbbing, sharp, or dull? Did they occur at a specific time of day or in a particular location on the head?
2. **Memory Issues**: What kind of memory problems were experienced? Was it difficulty recalling recent events, long-term memories, or both?
3. **Nausea and Vomiting**: Were these symptoms related to eating or did they occur independently? Was there any relief after vomiting?
4. **Imaging Results**: What did the imaging (e.g., MRI or CT scan) reveal? Were there any specific findings such as lesions, swelling, or abnormalities in the brain?
5. **Recent Travel or Exposure**: Since the patient recently emigrated from Mexico, was there any exposure to specific environments, infections, or other risk factors during or after the move?

Please provide this information so I can make a more accurate diagnosis.

1. **Headache Characteristics**: Not mentioned in the provided information.
2. **Memory Issues**: The mother reported increasing forgetfulness
3. **Nausea and Vomiting**: The nausea and vomiting occurred independently, and the patient experienced multiple episodes of vomiting.
4. **Imaging Results**: The initial head CT revealed ventriculomegaly indicative of obstructive hydrocephalus with an obstruction in the posterior third ventricle and an unusual appearance in the third ventricle region. Subsequent MR imaging after EVD placement showed migration of a cyst which restored normal cerebrospinal fluid flow.
5. **Recent Travel or Exposure**: The patient emigrated from Mexico a year prior. There is no specific mention of exposure to environments, infections, or other risk factors during or after the move.

Based on the provided information, the patient's symptoms (persistent headaches, nausea, vomiting, and memory issues) along with the imaging findings (ventriculomegaly, obstructive hydrocephalus, and a cyst in the third ventricle) strongly suggest a specific diagnosis. The migration of the cyst and the restoration of normal cerebrospinal fluid flow after intervention further support this conclusion. So the final answer is **colloid cyst of the third ventricle**

o3-mini

To clarify a few points:
1. You experienced persistent headaches for three weeks that recently worsened, with associated nausea, vomiting, and some memory difficulties.
2. Imaging studies revealed a brain lesion that required urgent intervention.
3. Following surgical (or other urgent) treatment, your symptoms resolved, and you were transferred for rehabilitation.
4. Your background—being a relatively young adult and recently emigrated from Mexico—can be associated with certain infectious risks.

Based on these details, the clinical picture is most consistent with the formation of a localized collection of pus in the brain, which we refer to as a brain abscess. This condition can present with progressively worsening headache, signs of increased intracranial pressure (nausea and vomiting), and cognitive disturbances such as memory issues. Urgent neurosurgical management is often required, followed by a period of rehabilitation, which aligns well with the history provided. So the final answer is brain abscess.

Figure 33: Baselines on case 2. Blue fonts represent physician input. "Not mentioned" means the required clinical operations are not provided in clinical information of the case reports. The specific prompt enables baselines to perform full-process diagnosis is in Supplementary Fig.21

Input: Chief Complaint:A 57-year-old woman reported a painless bump on her lower lip that has been slowly getting larger over the past three years. She had no history of injury or infection related to the area, and no other significant health issues were noted. After some procedures, the bump was removed, and the healing process went well with no signs of recurrence.
Question: What disease might this patient have?

Correct Answer: Schwannoma

DxDirector-7B:

<think> <sub-think 1> Let's analyze the patient's condition. The painless bump on the lower lip that has been growing over three years suggests a possible benign or malignant neoplasm. I need to gather more details about its characteristics. </sub-think 1>
[Question 1 to <Physician>]: What are the characteristics of the bump on the patient's lower lip?
[Answer 1]: The characteristics of the bump on the patient's lower lip include a painless, firm, non-tender swelling measuring 2 cm x 1 cm, which is gradually increasing in size over three years. There is no ulceration, discharge, or skin changes present.

<sub-think 2> The gradual increase in size over three years without any signs of infection or injury is concerning. I need to consider the potential for malignancy, particularly squamous cell carcinoma. </sub-think 2>
[Question 2 to <LLM>]: What type of lesions can present as painless, firm, non-tender swellings, and what are the key histopathological features to consider?
[Answer 2]: Painless, firm, non-tender swellings can be benign or malignant neoplasms, such as fibromas, adenomas, or more concerning, squamous cell carcinoma (SCC). Key histopathological features to consider include the appearance of the nuclei, presence of Verocay bodies, and the overall cellular architecture.

<sub-think 3> The histopathological findings are crucial for determining the nature of the lesion. The presence of Verocay bodies and strong S100 protein positivity suggests a neoplastic process involving squamous cells. </sub-think 3>
[Question 3 to <Physician>]: What are the specific histopathological findings that could indicate a particular type of lesion?
[Answer 3]: The histopathological findings indicating a specific type of lesion in this case are:

1. Predominantly hypercellular (Antoni A) areas with a fascicular pattern of spindled to ovoid nuclei.
2. Presence of Verocay bodies.
3. Strong and diffuse positivity for S100 protein on immunohistochemistry.

<sub-think 4> I need to confirm the typical characteristics of schwannomas to ensure this diagnosis fits the patient's condition. </sub-think 4>
[Question 4 to <LLM>]: What are the characteristics and typical histopathological findings of a schwannoma?
[Answer 4]: Schwannomas are benign neoplasms arising from the schwann cells of the peripheral nerves. They are typically firm, non-tender, and can present as painless bumps or lesions. Histopathologically, they are characterized by a hypercellular pattern with spindle-shaped cells, Verocay bodies, and strong S100 protein positivity.

<sub-think 5> Given the patient's age and the characteristics of the lesion, I can conclude that the most likely diagnosis is a schwannoma, particularly given the painless nature and gradual growth. </sub-think 5>
[Question 5 to <LLM>]: Given the patient's age and symptoms, what is the most likely diagnosis for this painless bump on the lip?
[Answer 5]: Given the patient's age, painless nature, and the gradual growth of the lesion along with the histopathological findings, the most likely diagnosis is a schwannoma (neurilemmoma) rather than any malignant process. </think>

<answer> [Final Content]: The patient has a painless bump on her lower lip that has been increasing in size over three years, with no signs of infection or injury [1]. Histopathological examination shows a hypercellular tumor with a fascicular pattern of spindled nuclei, Verocay bodies, and strong S100 protein positivity, indicating a neoplastic process involving squamous cells [2-3].
These findings align with characteristics typical of schwannomas, which are benign neoplasms [4-5].
So the final answer is a schwannoma (neurilemmoma). <\answer>

Reference:
[1] Symptoms observation …
[2] Skin Cancer -- History and Physical. A thorough skin examination is useful to identify premalignant and malignant skin lesions. It is important to note the location, texture, size, color, shape, borders, and any recent change\xa0of suspicious lesions. Premalignant actinic keratoses often present as rough, gritty skin papules on an erythematous base … (From https://www.ncbi.nlm.nih.gov/books/NBK557401/)
[3] Histopathological findings …
[4] InternalMed_Harrison. Neuroimaging findings suggestive of neurocysticercosis constitute the primary major diagnostic criterion (Fig. 260-1). These findings include cystic lesions with or without enhancement (e.g., ring enhancement), one or more nodular calcifications (which may also have associated enhancement), or focal enhancing lesions. … (From https://www.ncbi.nlm.nih.gov/books/NBK539707/)
[5] Surgery_Schwartz. of neural tissue, but rather disordered growth of Schwann cells and nerve axons, often at the site of previous trauma. They can present within surgical scar lines or at the site of previous trauma as flesh-colored papules or nodules and are typically painful. Schwannomas … (From https://pubmed.ncbi.nlm.nih.gov/17314203/)

Figure 34: Our DxDirector-7B on case 3. Blue fonts represent physician input, and others are generated by DxDirector-7B. In reference, the blue fonts record the physician actions, and others are retrieved from authoritative medical literature to provide a verification mechanism for each step in the reasoning. The reference numbers correspond to the steps of thinking-question-answer.

Input: Chief Complaint:A 57-year-old woman reported a painless bump on her lower lip that has been slowly getting larger over the past three years. She had no history of injury or infection related to the area, and no other significant health issues were noted. After some procedures, the bump was removed, and the healing process went well with no signs of recurrence.
Question: What disease might this patient have?

Correct Answer: Schwannoma

GPT-4o:

Hello! I understand you're experiencing a painless bump on your lower lip. Let's go through a few questions to gather more information.
1. Can you describe the bump? For instance, is it soft, hard, or fluid-filled?
2. Were there any changes in color of the bump or surrounding skin?
3. Have you noticed any other symptoms associated with the bump, such as swelling, or changes in sensation in the area?
4. Have you had any previous treatments or interventions for the bump before it was removed?
5. Do you have any personal or family history of skin conditions or oral lesions?

This will help me in understanding your situation better.

1. The bump is described as a firm, non-tender swelling.
2. There were no changes in color of the bump or surrounding skin mentioned.
3. The only associated symptom mentioned is that the swelling is painless; no other symptoms such as swelling or changes in sensation are noted.
4. There were no previous treatments or interventions mentioned for the bump before it was removed.
5. There is no personal or family history of skin conditions or oral lesions mentioned in the provided information.

Thank you for providing those details. Based on the characteristics of the bump—firm, non-tender, painless, and slow-growing over three years without any significant changes or associated symptoms—it suggests that it could be a benign condition.\n\nGiven the presentation and the description, it's likely that you had a benign fibrous lesion such as a **fibroma** or possibly a mucous retention cyst (mucocele). However, since the bump was firm and described as not fluid-filled, a **fibroma** is the most likely.\n\nSince you mentioned that the bump was successfully removed and there have been no signs of recurrence, that further supports that the diagnosis was benign. So the final answer is fibroma.

Deepseek-V3-671B:

Based on the description provided, the patient's condition aligns with a **mucocele**, a common benign lesion of the oral cavity. Mucoceles are typically caused by the accumulation of saliva due to a blocked or damaged minor salivary gland duct. They often present as painless, slow-growing bumps, particularly on the lower lip, and are not associated with a history of injury or infection. The fact that the bump was painless, slowly growing, and located on the lower lip strongly supports this diagnosis. Additionally, the successful removal and lack of recurrence further confirm this.\n\nSo the final answer is **mucocele**.

o3-mini:

Before I reach a final diagnosis, I'd like to ask you a few clarifying questions about the bump on your lower lip:
1. Could you describe its consistency? For example, did it feel soft and compressible or rather firm on palpation?
2. Did you notice any particular color or discoloration—perhaps a bluish or translucent appearance compared to your normal lip tissue?
3. Even if you haven't recalled any major injury, have you ever noticed yourself biting or irritating that area (even inadvertently)?
4. Was the size of the bump always steadily increasing, or did it sometimes fluctuate (for example, enlarging at certain times and then reducing slightly)?

Your answers to these points will help me determine whether the lesion is most consistent with a mucocele or another type of benign lesion. Please let me know.

1. Not mentioned in the provided information
2. Not mentioned in the provided information
3. Not mentioned in the provided information
4. The size of the bump was described as gradually increasing over three years, with no mention of fluctuations.

Based on the provided case details and considering the lesion's characteristics—a painless, gradually enlarging bump on the lower lip that was successfully removed with no recurrence—the findings are most consistent with a mucocele. So the final answer is mucocele.

Figure 35: Baselines on case 3. Blue fonts represent physician input. "Not mentioned" means the required clinical operations are not provided in clinical information of the case reports. The specific prompt enables baselines to perform full-process diagnosis is in Supplementary Fig.21
