# Supplementary material for: DxDirector: an agentic large language model driving the full-process clinical diagnosis
Source: Nat Commun. 2026 Apr 23;17:5614. doi: 10.1038/s41467-026-71928-5 (PMC13315919; doi:10.1038/s41467-026-71928-5)
Supplement: Supplementary file 2 — Reporting Summary [file 41467_2026_71928_MOESM2_ESM.pdf]

nature portfolio

Corresponding author(s): Liang Pang
Last updated by author(s): Jan 24, 2026

Reporting Summary

Nature Portfolio wishes to improve the reproducibility of the work that we publish. This form provides structure for consistency and transparency in reporting. For further information on Nature Portfolio policies, see our Editorial Policies and the Editorial Policy Checklist.

Statistics

For all statistical analyses, confirm that the following items are present in the figure legend, table legend, main text, or Methods section.

n/a	Confirmed	
☐	☒	The exact sample size (*n*) for each experimental group/condition, given as a discrete number and unit of measurement
☐	☒	A statement on whether measurements were taken from distinct samples or whether the same sample was measured repeatedly
☐	☒	The statistical test(s) used AND whether they are one- or two-sided<br>*Only common tests should be described solely by name; describe more complex techniques in the Methods section.*
☒	☐	A description of all covariates tested
☒	☐	A description of any assumptions or corrections, such as tests of normality and adjustment for multiple comparisons
☐	☒	A full description of the statistical parameters including central tendency (e.g. means) or other basic estimates (e.g. regression coefficient) AND variation (e.g. standard deviation) or associated estimates of uncertainty (e.g. confidence intervals)
☐	☒	For null hypothesis testing, the test statistic (e.g. *F*, *t*, *r*) with confidence intervals, effect sizes, degrees of freedom and *P* value noted<br>*Give P values as exact values whenever suitable.*
☒	☐	For Bayesian analysis, information on the choice of priors and Markov chain Monte Carlo settings
☐	☒	For hierarchical and complex designs, identification of the appropriate level for tests and full reporting of outcomes
☒	☐	Estimates of effect sizes (e.g. Cohen's *d*, Pearson's *r*), indicating how they were calculated

Our web collection on statistics for biologists contains articles on many of the points above.

Software and code

Policy information about availability of computer code

Data collection	Code for data collection was written in Python (3.10) Joblib (1.4.2) numpy (1.26.4) scipy (1.14.0) openai (0.27.2)
Data analysis	The training and evaluation framework used were written in Python (3.10), Pytroch (2.1.2), Transformers (4.37.2). We used DeepSpeed (0.12.6) and accelerate (0.21.0) to accelerate parallel training on multiple GPUs (4 Nvidia A100 80G GPUs). We used LLamaFactory (0.9.2) for online service of LLMs and used trl (0.9.6) for step-level preference optimization training. To prompt the future research, we promise to release all source codes to train our DxDirector-7B on Github, and the model weights on Huggingface after the external review. This ensures unrestricted access for anyone to use the code and model weights for any purpose, fostering future research and development.<br>Link to the custom code: Python: \url{https://github.com/python/cpython} Pytorch: \url{https://github.com/pytorch/pytorch} Transformers: \url{https://github.com/huggingface/transformers} DeepSpeed: \url{https://github.com/deepspeedai/DeepSpeed} accelerate: \url{https://github.com/huggingface/accelerate} LLamaFactory: \url{https://github.com/hiyouga/LlamaFactory}trl: \url{https://github.com/huggingface/trl}

For manuscripts utilizing custom algorithms or software that are central to the research but not yet described in published literature, software must be made available to editors and reviewers. We strongly encourage code deposition in a community repository (e.g. GitHub). See the Nature Portfolio guidelines for submitting code & software for further information.

Data

Policy information about availability of data

All manuscripts must include a data availability statement. This statement should provide the following information, where applicable:

- Accession codes, unique identifiers, or web links for publicly available datasets
- A description of any restrictions on data availability
- For clinical datasets or third party data, please ensure that the statement adheres to our policy

The training, testing and source data is available on Google Drive (link is \url{https://drive.google.com/file/d/1wvLuo58F24Xqh7yDY44cxXR1X487aRMJ/view?usp=drive_link})

Research involving human participants, their data, or biological material

Policy information about studies with human participants or human data. See also policy information about sex, gender (identity/presentation), and sexual orientation and race, ethnicity and racism.

Reporting on sex and gender	We did not perform sex and gender analysis. Our study did not involve human research participants where sex or gender considerations were explicitly needed, as the data were derived from retrospective clinical records (Peking University Third Hospital, IRB approval #IRB00006761-M20250173). We have confirmed that no sex- or gender-based analyses were performed in this study.
Reporting on race, ethnicity, or other socially relevant groupings	We did not perform race, ethnicity, or other socially relevant groupings analysis.
Population characteristics	N/A
Recruitment	Human participants were recruited through invitations from the research director within Peking University Third Hospital. The human research participants in this study were medical specialists invited from nine distinct clinical departments, including Gastroenterology, Nephrology, and Cardiovascular Medicine, within Peking University Third Hospital. These specialists were recruited to participate in a double-blind adjudication process where they independently diagnosed patient cases without seeing the AI's output, serving as a high-quality reference standard for evaluating the model's performance.<br>As for the self-selection bias, inherent selection bias exists as the study was conducted at a single top-tier medical institution using inpatient records, which typically involve more complex conditions than outpatient cases, potentially limiting the generalizability of the results to primary care settings or hospitals with different resource levels.
Ethics oversight	This research has been approved by the Ethics Review (IRB00006761-M20250173) of Peking University Third Hospital Medical Science Research Ethics Committee.

Note that full information on the approval of the study protocol must also be provided in the manuscript.

Field-specific reporting

Please select the one below that is the best fit for your research. If you are not sure, read the appropriate sections before making your selection.

☒ Life sciences ☐ Behavioural & social sciences ☐ Ecological, evolutionary & environmental sciences

For a reference copy of the document with all sections, see nature.com/documents/nr-reporting-summary-flat.pdf

Life sciences study design

All studies must disclose on these points even when the disclosure is negative.

Sample size	1. 10,178 samples for instruction-tuning, 23, 608 samples for step-Level strategy preference optimization<br>2. As for evaluation data: (1) NEJM Clinicopathologic Cases it covers 344 clinical cases published by the New England Journal of Medicine between 2014 and 2024. These cases are highly complex, rare, and educationally significant. (2) RareArena is a dataset of nearly 50,000 rare disease diagnoses extracted from case summaries in PubMed Central, covering 4,597 rare disease types. We use the rare disease confirmation of it, which covers 22,901 data samples. (3) ClinicalBench is a multi-departmental clinical diagnostic evaluation benchmark includes 1,500 real-world cases that cover 150 diseases. (4) US Medical License Exam is a set of 1,273 challenging medical questions in the US Medical License Exam. There are many tasks in this dataset such as diagnosis, differential diagnosis, treatment planning, and so on.
Data exclusions	We did not apply any special exclusion criteria to the datasets
Replication	We report the confidence intervals and we repeated our experiments 3 times on each dataset and found that the results are stable.
Randomization	Samples were randomly allocated to training and we used a random seed to control it.
Blinding	To ensure objective assessments and mitigate potential biases in human specialists scoring, a double-blind adjudication approach is implemented. In this approach, both human specialists and LLMs independently diagnose the same patient cases without exposure to each other's diagnostic outputs. Additionally, a third-party evaluation agent, utilizing both GPT-4o and Deepseek-V3, assigns scores based on the

alignment between LLM-generated diagnoses and those provided by medical specialists. The final score is calculated as the average of the scores given by GPT-4o and Deepseek-V3, thus ensuring robust and unbiased comparative assessment.

Reporting for specific materials, systems and methods

We require information from authors about some types of materials, experimental systems and methods used in many studies. Here, indicate whether each material, system or method listed is relevant to your study. If you are not sure if a list item applies to your research, read the appropriate section before selecting a response.

Materials & experimental systems

n/a	Involved in the study
☒	☐ Antibodies
☒	☐ Eukaryotic cell lines
☒	☐ Palaeontology and archaeology
☒	☐ Animals and other organisms
☐	☒ Clinical data
☒	☐ Dual use research of concern
☒	☐ Plants

Methods

n/a	Involved in the study
☒	☐ ChIP-seq
☒	☐ Flow cytometry
☒	☐ MRI-based neuroimaging

Clinical data

Policy information about clinical studies

All manuscripts should comply with the ICMJE guidelines for publication of clinical research and a completed CONSORT checklist must be included with all submissions.

Clinical trial registration

All real-world clinical data utilized in this research are exclusively for academic purposes, acquired ethically and legally, and have been reviewed and approved by the relevant institutional ethics committee (IRB00006761-M20250173), ensuring adherence to ethical and legal standards. Not registered on public clinical trial registries (e.g., ClinicalTrials.gov) as this is an observational (non-interventional, utilizing retrospective real-world clinical data) evaluation study. We also used the large-scale publicly available data for evaluation.

Study protocol

The real clinical diagnostic scenario is set within an officially certified Grade 3A hospitals in China. The involved patients are inpatients presenting with more complex conditions than typical outpatients. Consequently, LLMs must engage in intricate reasoning to gather comprehensive clinical information effectively. To safeguard patients from potential harm, the evaluation environment is structured as follows: patient behaviors and medical specialist operations during clinical diagnosis are fully recorded using actual inpatient records. Subsequently, two GPT-4o-based agents replicate precisely the recorded behaviors of patients and specialists throughout the diagnostic process. In evaluation, LLMs interact with these agents to drive the full-process diagnosis, initiating solely from the patient's vague chief complaint. Within this controlled environment, LLMs do not directly interact with real patients, and their diagnostic outputs undergo rigorous review by medical specialists, thereby effectively mitigating ethical risks and potential harm. The evaluation is performed on 160 cases across 9 different clinical departments including Gastroenterology, Nephrology, Dermatology, Cardiovascular Medicine, Infectious Diseases, Endocrinology, Pulmonology, General Surgery, and Pain Management.

To ensure objective assessments and mitigate potential biases in human specialists scoring, a double-blind adjudication approach is implemented. In this approach, both human specialists and LLMs independently diagnose the same patient cases without exposure to each other's diagnostic outputs. Additionally, a third-party evaluation agent, utilizing both GPT-4o and Deepseek-V3, assigns scores ranging from 0 to 10 based on the alignment between LLM-generated diagnoses and those provided by medical specialists. The final score is calculated as the average of the scores given by GPT-4o and Deepseek-V3, thus ensuring robust and unbiased comparative assessment. The assessment of whether the diagnoses generated by LLMs could fully replace those made by medical specialists also follows the same pattern by observing the decisions of the third party agent (can or cannot).

Data collection

The real clinical diagnostic scenario is set within an officially certified Grade 3A hospitals in China. The involved patients are inpatients presenting with more complex conditions than typical outpatients. Consequently, LLMs must engage in intricate reasoning to gather comprehensive clinical information effectively. To safeguard patients from potential harm, the evaluation environment is structured as follows: patient behaviors and medical specialist operations during clinical diagnosis are fully recorded using actual inpatient records. Subsequently, two GPT-4o-based agents replicate precisely the recorded behaviors of patients and specialists throughout the diagnostic process. In evaluation, LLMs interact with these agents to drive the full-process diagnosis, initiating solely from the patient's vague chief complaint. Within this controlled environment, LLMs do not directly interact with real patients, and their diagnostic outputs undergo rigorous review by medical specialists, thereby effectively mitigating ethical risks and potential harm. The evaluation is performed on 160 cases across 9 different clinical departments including Gastroenterology, Nephrology, Dermatology, Cardiovascular Medicine, Infectious Diseases, Endocrinology, Pulmonology, General Surgery, and Pain Management.

Outcomes

To ensure objective assessments and mitigate potential biases in human specialists scoring, a double-blind adjudication approach is implemented. In this approach, both human specialists and LLMs independently diagnose the same patient cases without exposure to each other's diagnostic outputs. Additionally, a third-party evaluation agent, utilizing both GPT-4o and Deepseek-V3, assigns scores ranging from 0 to 10 based on the alignment between LLM-generated diagnoses and those provided by medical specialists. The final score is calculated as the average of the scores given by GPT-4o and Deepseek-V3, thus ensuring robust and unbiased comparative assessment. The assessment of whether the diagnoses generated by LLMs could fully replace those made by medical specialists also follows the same pattern by observing the decisions of the third party agent (can or cannot).

Plants

Seed stocks	N/A
Novel plant genotypes	N/A
Authentication	N/A
