# Supplementary figures and images for: DxDirector: an agentic large language model driving the full-process clinical diagnosis
Source: Nat Commun. 2026 Apr 23;17:5614. doi: 10.1038/s41467-026-71928-5 (PMC13315919; doi:10.1038/s41467-026-71928-5)

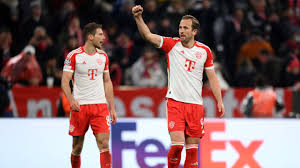

Supplement: Supplementary file 5 — Source Data 2 [file 41467_2026_71928_MOESM5_ESM.zip › Source-Code/Inference/LLaMA-Factory-main/data/mllm_demo_data/1.jpg]

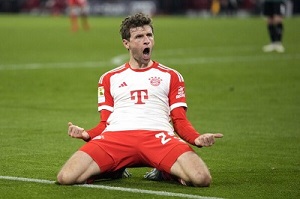

Supplement: Supplementary file 5 — Source Data 2 [file 41467_2026_71928_MOESM5_ESM.zip › Source-Code/Inference/LLaMA-Factory-main/data/mllm_demo_data/2.jpg]

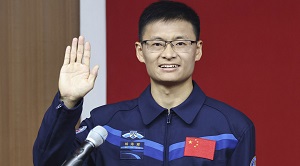

Supplement: Supplementary file 5 — Source Data 2 [file 41467_2026_71928_MOESM5_ESM.zip › Source-Code/Inference/LLaMA-Factory-main/data/mllm_demo_data/3.jpg]
