# Supplementary material for: DxDirector: an agentic large language model driving the full-process clinical diagnosis
Source: Nat Commun. 2026 Apr 23;17:5614. doi: 10.1038/s41467-026-71928-5 (PMC13315919; doi:10.1038/s41467-026-71928-5)
Supplement: Supplementary file 3 — Peer Review file [file 41467_2026_71928_MOESM3_ESM.pdf]

nature portfolio

Peer Review File

DxDirector: An Agentic Large Language Model Driving the Full-process Clinical Diagnosis

Corresponding Author: Professor Liang Pang

This file contains all reviewer reports in order by version, followed by all author rebuttals in order by version.

Version 0:

Reviewer comments:

Reviewer #2

(Remarks to the Author)
This paper aims to reverse the current proposed Doctor-AI interaction paradigm by proposing a primarily AI-driven and Doctor assisted framework for performing diagnostic tasks, alongside an accompanying fine-tuned model. While interesting, there are several major concerns from a purely technical perspective particularly with respect to evaluation that directly impact the rigor of this study:

- Excepting the closed-weight GPT o-series, the baselines compared are non-reasoning models (as characterized by explicit training for an internal CoT phase prior to final answer generation, e.g., DeepSeek R1). Considering that your proposed paradigm is highly analogous to elucidating the internal CoT process and injecting human input at each step, reasoning models seem a more reasonable comparison as a baseline.

- The datasets are generated by GPT and subsequently purportedly human reviewed. What is the annotation/review criteria? What level of agreement was there between human reviewers and the generated dataset?

- “We determine whether an operation is helpful for diagnosis by assessing whether it appears in the case report provided from medical specialists” How was this matching done? Is it via GPT/similar evaluation using LLMs as a judge?

- “We introduce a third-party agent to score an LLM based on the degree of match between LLM’s diagnosis and human physicians’ diagnosis. The third-party agent is based on GPT-4o and Deepseek-V3, and the average of the scores given by them is taken as the actual score. The score range is between 0 and 10.” This is highly suspect, given LLM predisposition for integer scores and certain values within the score range that does not correspond to human judgements (see G-EVAL paper by Liu et al.). Was this reviewed by humans at all? If so what was the agreement and why are there two models in this third-party agent?

- Throughout the manuscript but most pertinently in Figure 12 denotes “Accuracy of Diagnosis” is mentioned but USMLE dataset/answers contains more than just diagnosis as acknowledged by the authors themselves. How was this conflict resolved?

- Evaluation of 4.4.2 seems to be done? Given that this is an active research question, it may be beneficial to at least perform a sample and verify the retrieval accuracy, even if a systemic evaluation is not feasible

Some additional minor comments:
- Multiple minor typos/grammatical errors, may benefit from a round of proofreading

(Remarks on code availability)
This seems to only contain code for training/finetuning? Is there any plan to release evaluation code as well?

Also, this codebase seems to also (in addition to the authors own code) contain an amalgamation of the source code of other

libraries (e.g., deepspeed, transformers, amongst others) is there a listed diff of modifications made or can these be substituted with the existing open-source libraries as is?

Reviewer #4

(Remarks to the Author)
The manuscript presents a technically interesting model of LLM interaction for diagnostic decision making called, DxDirector-7B, with good benchmark performance. However, its clinical relevance may be overstated. In real-world practice, physicians prioritize risk management, patient safety, and timely therapeutic decisions, which are not adequately captured by accuracy-focused evaluations. A model that identifies the correct diagnosis may still be clinically insufficient if it fails to recognize and escalate high-risk conditions. Additionally, the proposed reconfiguration of physician roles—as assistants to an AI system—raises concerns about clinical autonomy, ethical responsibility, and professional judgment, which are central to medical practice and patient trust. I recommend the authors clarify the distinction between benchmark success and clinical applicability and consider reframing the model's role as a collaborative tool rather than a directive agent.

So, while the results maybe interesting from a technical standpoint, but the paper overstates its real-world applicability by not fully addressing the human, ethical, and contextual dimensions of clinical practice. Translating it into clinical workflows would require careful integration, trust-building, and alignment with how care is delivered.

In the remaining review, I will consider the study as an assessment of a restructured LLM interaction for clinical decision making, which places AI in the lead role to guide clinical reasoning with minimal human input. The paper contrasts this with what one might naively do by presenting all the info to an LLM and ask for its diagnostic decision. Results show that the AI driven approach has advantages including better diagnostic accuracy.

I think the paper's significant contribution is the methodology to evaluate an interaction model between human and an LLM (or the prompt engineering, if you will) for diagnostic purposes. To simulate diagnostic workflows, they reconstruct four public datasets (NEJM Clinicopathologic Cases, RareArena, ClinicalBench, and USMLE) by masking detailed clinical information and requiring the model to actively infer or request it. A simulated physician agent (powered by GPT-4o) provides responses to the model's clinical queries, mimicking real-world physician collaboration. Performance is assessed using diagnostic accuracy, number and utility of physician-requested operations, and alignment with human specialists. Additionally, real-world evaluations were conducted in a top-tier hospital using 160 inpatient cases across 9 departments, with double-blind adjudication by medical experts and third-party LLM agents to assess diagnostic quality and substitution potential.
In terms of how the methodology and results compare, the paper has not provided sufficient details. For example, how does the proposed approach of "slow thinking" compare with prompt engineering strategies and sophisticated orchestration strategies within an agent. In other words, since the reasoning strategies of a chatbot (like, ChatGPT) are improving how does the proposed strategy compare.

Another concern I have is the use of LLMs to simulate interactions with patients and physicians. While this is an expedient way, are you multiplying errors? Or are you ending up leveraging synergies between them?

If the customized versions of the benchmarks, prompts, and agent code are available, it will enhance reproducibility.

(Remarks on code availability)
Code is not available.

Version 1:

Reviewer comments:

Reviewer #2

(Remarks to the Author)
I thank the authors for their careful revisions. While they have satisfactorily addressed most of my comments, I do have one additional comment in response to their point-by-point.

With respect to your response to reviewer #2 comment 2, while I appreciate the inclusion of evaluation criteria, the criteria used seem somewhat subjective and would benefit from additional detail being listed in the manuscript.

Some examples of this include items such as: what is the definition of "consistent" how was the threshold between consistent/inconsistent defined? What is an "ambiguous" expression? How was "reducing the cognitive workload of clinicians" defined?

While the answers to these may seem obvious from a plain english perspective, this may differ between individual annotators. Additionally, some analysis of mismatches between inter-annotators may be beneficial.

(Remarks on code availability)
Thank you to the authors for addressing my concerns w.r.t. codebase

Reviewer #4

(Remarks to the Author)
I reviewed authors' response specifically to my comments and questions as well as in the manuscript. I think the paper is acceptable for publication now.

(Remarks on code availability)
I noticed that the code is available now in the supplementary information.

Open Access This Peer Review File is licensed under a Creative Commons Attribution 4.0 International License, which permits use, sharing, adaptation, distribution and reproduction in any medium or format, as long as you give appropriate credit to the original author(s) and the source, provide a link to the Creative Commons license, and indicate if changes were made.
In cases where reviewers are anonymous, credit should be given to 'Anonymous Referee' and the source.
The images or other third party material in this Peer Review File are included in the article's Creative Commons license, unless indicated otherwise in a credit line to the material. If material is not included in the article's Creative Commons license and your intended use is not permitted by statutory regulation or exceeds the permitted use, you will need to obtain permission directly from the copyright holder.
To view a copy of this license, visit https://creativecommons.org/licenses/by/4.0/

Dear Reviewers,

We sincerely thank you for the opportunity to revise our manuscript. We are deeply grateful to the reviewers for their insightful and constructive comments. These suggestions have been invaluable in helping us improve the technical rigor of our evaluation, clarify the specific criteria for human review, and properly contextualize the clinical relevance and safety of our proposed system.

In accordance with your recommendations, we have performed a major revision of the manuscript. specifically, we have:

1. Enhanced Technical Rigor: Added reasoning models (DeepSeek-R1) as baselines and conducted rigorous human verification to validate our automated evaluation metrics.
2. Refined Clinical Framework: Redefined the physician-AI relationship as "collaborative" and implemented a "High-Risk Escalation Policy" with human-in-the-loop safeguards to prioritize patient safety and physician autonomy.
3. Improved Clarity: Comprehensively polished the manuscript to address terminology inconsistencies (e.g., USMLE task accuracy) and improve readability.

We believe that these extensive revisions have significantly strengthened the paper and fully addressed the concerns raised. Below, we provide a point-by-point response to each specific comment.

Reviewer #2:

Reviewer’s Comment 1:
This paper aims to reverse the current proposed Doctor-AI interaction paradigm by proposing a primarily AI-driven and Doctor assisted framework for performing diagnostic tasks, alongside an accompanying fine-tuned model. While interesting, there are several major concerns from a purely technical perspective particularly with respect to evaluation that directly impact the rigor of this study:

- Excepting the closed-weight GPT o-series, the baselines compared are non-reasoning models (as characterized by explicit training for an internal CoT phase prior to final answer generation, e.g., DeepSeek R1). Considering that your proposed paradigm is highly analogous to elucidating the internal CoT process and injecting human input at each step, reasoning models seem a more reasonable comparison as a baseline.

Response 1:

We appreciate the reviewer's insightful comment and agree that reasoning models would be a more relevant baseline for comparison, given the nature of our approach.

On the inclusion of reasoning models.

In response to the reviewer's suggestion, we have added DeepSeek-R1 (a reasoning model) as an additional baseline (See **Figure 4, Figure 5, Figure 6, Figure 7, Figure 8, Figure 9, Figure 10, Figure 11, Figure 12, Figure 13**). We also clarify that the models mentioned in the original paper, including OpenAI o1 and OpenAI o3, are reasoning models, as they incorporate an internal Chain-of-Thought phase during answer generation.

New experiments and findings.

Our new experiments demonstrate that our proposed paradigm continues to significantly outperform DeepSeek-R1, further highlighting the effectiveness of our approach. These results have been added to the manuscript, with detailed comparisons of reasoning-based models.

We appreciate the reviewer's valuable feedback, which has helped improve the depth of our evaluation.

Reviewer's Comment 2:

- The datasets are generated by GPT and subsequently purportedly human reviewed. What is the annotation/review criteria? What level of agreement was there between human reviewers and the generated dataset?

Response 2:

We appreciate the reviewer's valuable question regarding the human review process and the criteria used for evaluating the generated datasets. Below, we provide a detailed explanation of the annotation/review criteria, as well as the level of agreement between the human reviewers and the generated dataset.

Review Criteria:

Human reviewer are all experienced clinicians and they are instruct to evaluate the AI-generated datasets based on the following five key criteria:

1. **Medical Accuracy:**
   Evaluation of whether the reasoning process aligns with established clinical knowledge, including whether diagnostic steps are consistent with actual clinical practice and whether any obvious medical errors or inconsistencies are present.

2. **Logical Consistency of Reasoning:**
   Assessment of whether each step in the reasoning is clear and coherent, ensuring that the

steps are logically connected and that the overall reasoning chain is complete.

3. **Clinical Appropriateness and Safety:**
   Evaluation of whether the reasoning appropriately considers the patient's clinical context, including history, symptoms, and signs, and whether the model avoids overgeneralization or neglecting key factors.

4. **Clarity and Precision of Language:**
   Review of the language used in the generated output to ensure that it is clear, precise, and free from ambiguous or unclear expressions.

5. **Clinical Collaboration Efficiency:**
   Assessment of the extent to which the AI-generated reasoning can potentially reduce the cognitive workload for clinicians, facilitating clinical decision-making.

Review Process and Agreement:
The human reviewers assigned scores for each of these dimensions on a scale from 0 to 10. The average scores across five clinicians for the entire dataset were as follows:

- Medical Accuracy: 9.04
- Logical Consistency of Reasoning: 9.85
- Clinical Appropriateness and Safety: 9.43
- Clarity and Precision of Language: 8.97
- Clinical Collaboration Efficiency: 8.44

These scores indicate a high level of agreement among the reviewers, particularly in terms of logical consistency and clinical appropriateness. We have further analyzed the inter-rater reliability (Intraclass Correlation Coefficient (ICC)=0.85), which shows consistent agreement across reviewers, ensuring the robustness of the human evaluation process.

In our revised version, we added a description of this part in **Section 6 of the Supplementary**. We hope this clarifies the human review process and the criteria used. We appreciate the reviewer's attention to this detail, which has allowed us to further highlight the rigor and reliability of our evaluation methodology.

Reviewer's Comment 3:

- "We determine whether an operation is helpful for diagnosis by assessing whether it appears in the case report provided from medical specialists" How was this matching done? Is it via GPT/similar evaluation using LLMs as a judge?

Response 3:

Thank you for your insightful question. Yes, the matching process is indeed performed using an LLM (GPT-4o) in our large-scale automated evaluation. GPT-4o is instructed to determine whether a given operation is mentioned in the case report provided by the medical specialists. Essentially, this task is similar to information extraction and matching, which is relatively straightforward for an LLM to handle [1,2]. We hope this provides clarity on the process and appreciate your attention to this detail. In our revised version, we have clarified this point in **Lines 981-986** in Section 4.6.2**.**

[1] Xu D, Chen W, Peng W, et al. Large language models for generative information extraction: A survey[J]. Frontiers of Computer Science, 2024, 18(6): 186357.

[2] Polak M P, Morgan D. Extracting accurate materials data from research papers with conversational language models and prompt engineering[J]. Nature Communications, 2024, 15(1): 1569.

Reviewer's Comment 4:

- "We introduce a third-party agent to score an LLM based on the degree of match between LLM's diagnosis and human physicians' diagnosis. The third-party agent is based on GPT-4o and Deepseek-V3, and the average of the scores given by them is taken as the actual score. The score range is between 0 and 10." This is highly suspect, given LLM predisposition for integer scores and certain values within the score range that does not correspond to human judgements (see G-EVAL paper by Liu et al.). Was this reviewed by humans at all? If so what was the agreement and why are there two models in this third-party agent?

Response 4:

We thank the reviewer for the careful reading and constructive feedback. We appreciate the opportunity to clarify our evaluation design and the role of the third-party agent.

1. **About LLM-based judgements:**

We appreciate the reviewer pointing out the G-EVAL paper by Liu et al. regarding integer bias in LLM scoring. We agree that this is a critical consideration. However, it is important to distinguish the setting in G-EVAL (reference-free quality assessment) from our specific experimental setup.

In our study, the evaluation is reference-based. The judge agent is not asked to score the quality of a diagnosis in a vacuum (which is prone to the biases mentioned in G-EVAL); rather, it is tasked with measuring the semantic alignment between the model's output and a detailed physician-given reference (the "Gold Standard" case report). This formulation

reduces the task to a comparison of factual alignment rather than subjective quality scoring. While human evaluation remains the gold standard, exclusive reliance on it for large-scale benchmarks risks subjectivity (e.g., fatigue, inter-rater inconsistency). Recent literature suggests that for reference-based tasks, LLMs can serve as reliable, scalable proxies that correlate well with human experts [1,2,3] while mitigating specific human inconsistencies [4,5,6].

[1] Hsu T Y, Huang C Y, Rossi R, et al. GPT-4 as an Effective Zero-Shot Evaluator for Scientific Figure Captions[C]//2023 Findings of the Association for Computational Linguistics: EMNLP 2023. Association for Computational Linguistics (ACL), 2023: 5464-5474.

[2] Proietti L, Perrella S, Navigli R. Has Machine Translation Evaluation Achieved Human Parity? The Human Reference and the Limits of Progress[J]. arXiv preprint arXiv:2506.19571, 2025.

[3] Henkel O, Hills L, Boxer A, et al. Can large language models make the grade? an empirical study evaluating llms ability to mark short answer questions in k-12 education[C]//Proceedings of the Eleventh ACM Conference on Learning@ Scale. 2024: 300-304.

[4] A framework for human evaluation of large language models in healthcare derived from literature review

[5] Evaluating evidence-based health information from generative AI using a cross-sectional study with laypeople seeking screening information

[6] ConSiDERS-The-Human Evaluation Framework: Rethinking Human Evaluation for Generative Large Language Models

2. About human review and agreement:

To address your concern directly and validate our automated metric, we conducted a rigorous human verification study in the revised manuscript. We randomly sampled 10% of the test cases across baseline LLMs and DxDirector-7B and introduced three independent medical specialists to score them blindly.

We compared the automated agent scores against these human scores and now report the following agreement statistics in **Section 2.6 (Lines 416-421)**:

- Ranking Consistency: The rank ordering of models under human scoring matches the agent scoring with a **Kendall's τ of 0.83**, indicating very strong alignment in model ranking.
- Reliability: The inter-rater reliability between clinicians was **Intraclass Correlation**

Coefficient (ICC)=0.79, suggesting that the task definition is robust.

These results confirm that our third-party agent is a reliable proxy for human specialist judgment in this specific context.

3. Why two models in the third-party agent?

We use two independently trained models from different organizations (GPT-4o and DeepSeek-V3) to mitigate single-model biases and reduce variance in the judge. Averaging their scores yields a more stable, conservative estimate of alignment to the physician reference. We have clarified this in **Lines 387-391** of the revised manuscript.

Reviewer's Comment 5:

- Throughout the manuscript but most pertinently in Figure 12 denotes "Accuracy of Diagnosis" is mentioned but USMLE dataset/answers contains more than just diagnosis as acknowledged by the authors themselves. How was this conflict resolved?

Response 5:

We thank the reviewer for their keen attention to detail. We agree that labeling the metric as "Accuracy of Diagnosis" was imprecise given that the USMLE dataset encompasses a diverse range of clinical reasoning tasks beyond establishing a diagnosis (e.g., pathophysiology, management, pharmacology).

To resolve this inconsistency and accurately represent our evaluation:

1. **Label Correction:** We have renamed the y-axis in **Figure 12** from "Accuracy of Diagnosis" to **"Overall Task Accuracy"**. This explicitly communicates that the metric represents the aggregated performance across all question types in the USMLE dataset.
2. **Explicit Breakdown:** We have reinforced the connection between the aggregate score in Figure 12 and the fine-grained analysis in **Figure 13**. The text now clearly states that while Figure 12 presents the mean performance, Figure 13 visualizes the specific accuracy across the **12 distinct clinical tasks** (including Differential Diagnosis, Treatment, Pathophysiology, etc.) acknowledged by the reviewer.

We believe these changes ensure the terminology precisely matches the experimental design.

Reviewer's Comment 6:

- Evaluation of 4.4.2 seems to be done? Given that this is an active research question, it may

be beneficial to at least perform a sample and verify the retrieval accuracy, even if a systemic evaluation is not feasible

Response 6:

We thank the reviewer for the thoughtful suggestion. We agree that verifying the retrieval component in Section 4.4.2 is important. Accordingly, we have added a targeted experiment to assess retrieval accuracy.

Protocol.
On a held-out sample of test queries used in Section 4.4.2, we measure Recall@k, defined as the proportion of queries for which at least one accurate reference document appears in the top-k retrieved items.

Results.

- Recall@1: 70.58%
- Recall@5: 79.80%
- Recall@10: 93.25%

These results indicate that while single-hit retrieval leaves room for improvement, relevant evidence is present in the top-10 for the vast majority of cases, which supports the practicality of our downstream evaluation that provide references from authoritative medical literature for the model's output.

We have added this evaluation to **Section 4.4.2 (Lines 838-845)** and included **Table 1** to present these statistics clearly. We appreciate the guidance, as this quantitative validation significantly strengthens the empirical grounding of our pipeline.

Reviewer's Comment 7:

Some additional minor comments:
- Multiple minor typos/grammatical errors, may benefit from a round of proofreading

Response 7:

We thank the reviewer for pointing this out. We have conducted a comprehensive proofreading of the entire manuscript to correct typos, grammatical errors, and phrasing inconsistencies. We believe the readability and flow of the text have been significantly improved.

Reviewer's Comment 8:

Reviewer #2 (Remarks on code availability):

This seems to only contain code for training/finetuning? Is there any plan to release evaluation code as well?

Response 8:

We have submitted the relevant evaluation code as supplementary material. These scripts will also be permanently hosted on our public GitHub repository alongside the training code upon publication.

Reviewer's Comment 9:

Also, this codebase seems to also (in addition to the authors own code) contain an amalgamation of the source code of other libraries (e.g., deepspeed, transformers, amongst others) is there a listed diff of modifications made or can these be substituted with the existing open-source libraries as is?

Response 9:

We thank the reviewer for this observation. We included the source code of these standard libraries (DeepSpeed, Transformers) within the submission solely to ensure a self-contained, reproducible environment and to prevent version mismatches (dependency drift) during the peer review process.

We have not modified the source code of these libraries. Therefore, they can be substituted with the standard open-source versions installed via package managers (e.g., pip or conda) without any issues.

For the final public GitHub release, we will remove these bundled libraries and instead provide a strict requirements.txt / Dockerfile to align with community best practices and keep the repository lightweight.

Reviewer #4 (Remarks to the Author):

Reviewer's Comment 1:

The manuscript presents a technically interesting model of LLM interaction for diagnostic decision making called, DxDirector-7B, with good benchmark performance. However, its clinical relevance may be overstated. In real-world practice, physicians prioritize risk management, patient safety, and timely therapeutic decisions, which are not adequately

captured by accuracy-focused evaluations. A model that identifies the correct diagnosis may still be clinically insufficient if it fails to recognize and escalate high-risk conditions. Additionally, the proposed reconfiguration of physician roles—as assistants to an AI system—raises concerns about clinical autonomy, ethical responsibility, and professional judgment, which are central to medical practice and patient trust. I recommend the authors clarify the distinction between benchmark success and clinical applicability and consider reframing the model's role as a collaborative tool rather than a directive agent.

So, while the results maybe interesting from a technical standpoint, but the paper overstates its real-world applicability by not fully addressing the human, ethical, and contextual dimensions of clinical practice. Translating it into clinical workflows would require careful integration, trust-building, and alignment with how care is delivered.

Response 1:

We sincerely thank the reviewer for the thoughtful and constructive comments. We fully agree with the reviewer that benchmark accuracy alone is insufficient to demonstrate real-world clinical applicability. Patient safety, timely risk management, and the preservation of physician autonomy are indeed central to clinical practice.

We acknowledge that our original framing of the physician as an "assistant" to the AI may have inadvertently minimized the crucial role of human judgment. Our core intention was to propose a system that alleviates the burden of repetitive, low-risk information gathering (the "process"), allowing physicians to focus on high-stakes decision-making. In existing real clinical practice, AI systems are often asked to produce a final diagnosis or answer specific medical questions. However, the most labor-intensive part of care is driving the overall clinical process—a sequence of low-risk, high-cognitive steps that AI can reliably learn and execute (e.g., analyzing the patient's condition, collecting information, and determining the next steps, etc.). The main contribution of our DxDirector is that we redefine the role of AI in clinical practice. It should automate the entire clinical diagnostic process, thereby reducing the workload of physicians on repetitive and low-risk reasoning tasks. Real-world testing and physician feedback indicate that this approach is more popular with physicians than traditional methods of answering medical questions and significantly reduces their clinical workload.

To address your concerns comprehensively, we have implemented major revisions to both the system architecture and the manuscript:

1. **Reframing the Physician–AI Relationship:** We have softened our language throughout

the Abstract, Introduction, and Discussion to position DxDirector-7B as a collaborative tool that assists physicians in repetitive or low-risk reasoning steps, while physicians retain autonomy, ethical responsibility, and final decision-making authority. These revisions can be found in our **new title**, **Lines 12-16, Lines 51-54, Lines 60-67, Lines 553-569, etc.**

2. **High-Risk Escalation Policy and Human-in-the-Loop Safeguards.**
   We follow your suggestions to add a High-Risk Escalation Policy and Human-in-the-Loop Safeguards to our DxDirector. It means that acute or high-risk scenarios including critical vital signs or laboratory/imaging alerts suggestive of imminent harm, suspected suicidal intent, legal/forensic implications, substance-related or ethical concerns are escalated to physicians, at which point the AI does not further intervene. We detail this mechanism in the **new Section 4.5 (Lines 847-930)** of our revised manuscript. This policy preserves physician autonomy, concentrates expert attention where it matters most, and prevents inappropriate AI involvement in sensitive decisions.

   To validate this new safety pipeline, we conducted a dedicated evaluation using both large-scale simulations and real-world critical cases. The results verify that our escalation pipeline is highly effective, achieving a **Recall of 98.59%** and **Precision of 92.47%** in identifying dangerous situations that require human intervention. These results are reported in **Section 4.5.5 (Lines 919-930).**

3. **Ethical and Professional Responsibility**: We clarified our description of the accountability framework to make clear that DxDirector-7B can not only reduce physician workload on repetitive and low-risk reasoning tasks, but also support traceability and transparency in the decision-making process, thus reinforcing patient trust and clinical responsibility. Please see **Section 2.8.**

4. **Clarifying Clinical Relevance**: We added a dedicated subsection entitled *"Clinical Relevance and Safety"* in the Discussion (**Lines 553-569**) to highlight how DxDirector-7B addresses high-risk conditions. Specifically, we now describe mechanisms by which the model flags potentially urgent cases and escalates them directly to physicians, ensuring that risk management and patient safety remain the highest priority.

We believe these changes bridge the gap between technical innovation and clinical applicability.

Reviewer's Comment 2:
In the remaining review, I will consider the study as an assessment of a restructured LLM interaction for clinical decision making, which places AI in the lead role to guide clinical

reasoning with minimal human input. The paper contrasts this with what one might naively do by presenting all the info to an LLM and ask for its diagnostic decision. Results show that the AI driven approach has advantages including better diagnostic accuracy.

I think the paper's significant contribution is the methodology to evaluate an interaction model between human and an LLM (or the prompt engineering, if you will) for diagnostic purposes. To simulate diagnostic workflows, they reconstruct four public datasets (NEJM Clinicopathologic Cases, RareArena, ClinicalBench, and USMLE) by masking detailed clinical information and requiring the model to actively infer or request it. A simulated physician agent (powered by GPT-4o) provides responses to the model's clinical queries, mimicking real-world physician collaboration. Performance is assessed using diagnostic accuracy, number and utility of physician-requested operations, and alignment with human specialists. Additionally, real-world evaluations were conducted in a top-tier hospital using 160 inpatient cases across 9 departments, with double-blind adjudication by medical experts and third-party LLM agents to assess diagnostic quality and substitution potential.
In terms of how the methodology and results compare, the paper has not provided sufficient details. For example, how does the proposed approach of "slow thinking" compare with prompt engineering strategies and sophisticated orchestration strategies within an agent. In other words, since the reasoning strategies of a chatbot (like, ChatGPT) are improving how does the proposed strategy compare.

Response 2:

We thank the reviewer for highlighting the importance of situating our methodology within the broader landscape of prompt engineering and agent-based orchestration strategies. Your observation helped us sharpen both the positioning of our contribution and the empirical contrasts to alternative strategies. Below we respond point-by-point and outline concrete manuscript updates.

What we already compare against (and how)

As reported in Section 2.2 (**Lines 224-227**), we compare to strong commercial and open-source LLMs (e.g., GPT-4o, o3-mini, DeepSeek). These baselines are given task-specific prompts (please see **Figure 22** and **Figure 23** in Supplementary) that enables them perform multiple rounds of conversations for clinical diagnosis. In other words, our baselines already include prompt-engineering and orchestration that are favorable to them rather than "zero-shot" settings.

Relationship to improving base chatbots

Advances in general-purpose chatbots directly benefit our system because our method is

layered on top of a base model. Our step-level training can be applied to newer/stronger base models to further enhance performance. Conversely, without the proposed training, a stronger base chatbot alone does not acquire the interactive clinical reasoning skills and workload-aware behaviors we target.

Why our "slow thinking" is different from (and complementary to) prompting/orchestration

Our slow-thinking policy is *learned* via our designed step-level preference optimization on large-scale medical data. At each reasoning step, the model is optimized against a measurable objective that balances *diagnostic correctness* and *physician workload*. The gradients update model parameters, *persistently internalizing* medical knowledge, reasoning behaviors, and strategy preferences. At inference time, the model therefore tends to produce stepwise plans that are both accurate and workload-reducing.

What prompt engineering/orchestration cannot guarantee.

- **No quantified objective:** Prompt templates (even with sophisticated control flow) do not provide a numeric supervision signal about "which stepwise strategy is better." They steer behavior but do not *optimize* it against outcomes like physician effort.
- **Stability and generalization:** Prompts are known to be sensitive; minor wording changes can yield noticeably different outputs. Without large, domain-specific training, it is difficult to guarantee stable, department-wide generalization across diseases and settings.
- **Compliance and deployment:** Prompt-only use of third-party APIs can require transmitting sensitive records to external services, raising privacy/compliance burdens (e.g., HIPAA). Our approach supports fine-tuning and local/on-prem deployment, so institutions can keep patient data in-house while achieving performance that rivals or exceeds third-party chatbots. This materially simplifies privacy reviews and clinical governance.

In short, as reasoning-capable chatbots improve, our approach remains orthogonal and complementary: it converts general capability into clinically grounded, workload-aware behavior via step-level optimization on large medical datasets. Prompt engineering and agent orchestration are valuable, but without outcome-linked training they cannot by themselves deliver the same stability, generalization, and compliance-ready deployment that our slow-thinking models achieve.

We appreciate the reviewer's suggestion; the revision incorporates the clarifications above to make the methodology and empirical comparisons more explicit.

Reviewer's Comment 3:

Another concern I have is the use of LLMs to simulate interactions with patients and physicians. While this is an expedient way, are you multiplying errors? Or are you ending up leveraging synergies between them?

Response 3:

We sincerely thank you for this thoughtful and stimulating comment. We address your concern in two parts: (A) the use of an LLM-powered simulated physician agent for scalable testing, and (B) the end-to-end collaboration among the LLM, physicians, and patients in our DxDirector framework.

A) On using an LLM to simulate physician–patient interactions during evaluation

We agree this is a critical point and appreciate the opportunity to clarify. Running full physician–patient interactions at scale on large test sets is prohibitively costly. Using an LLM to simulate these interactions allows us to construct comprehensive, end-to-end diagnostic training and evaluation scenarios rapidly and systematically.

Why this does not multiply errors. In our setup, the simulated physician agent (GPT-4o) is largely a bounded extractor of facts from the case record in response to the model's clinical queries (e.g., labs, symptoms, imaging findings), rather than an open-ended generator. This task is relatively straightforward for an LLM to handle [1,2]. In our audit on the test split, the agent's information-extraction accuracy was 99.45%. Even under a conservative multi-turn assumption, the compounded accuracy remains high: $0.9945^{10} \approx 94.63\%$. This level is comparable to reported ranges for practicing physicians on tasks such as chest X-ray and ECG interpretation, laboratory test interpretation and communication, and point-of-care ultrasound use [3–8].

Not a deployment constraint. Importantly, this simulation is a scalability device for evaluation, not a requirement for deployment. In practical use, clinicians—not a simulated agent—conduct the interactions, so there is no risk of synthetic multi-turn error accumulation in real workflows.

Synergy rather than amplification. We observe that the model and the simulated agent exhibit *complementarity*: the LLM handles clinical reasoning while the agent supplies only the requested facts. This design improved both diagnostic accuracy and the efficiency of physician effort.

Accountability and containment of errors. We also introduce an accountability framework

that cleanly separates inputs and outputs between the LLM and the physician agent . This separation supports auditability and limits any potential error propagation across components. Empirically, this structure achieves the best precision/recall for attributing misdiagnoses to the correct party among all baselines (**Section 2.8**).

B) On DxDirector's LLM–physician–patient collaboration: error multiplication vs. synergy

We do not observe error amplification; rather, the system is designed for complementary strengths:

LLM (AI).

- Knowledge & reasoning: Accurately and quickly answers medical knowledge queries and performs complex reasoning (differential diagnosis, complication risk estimation, pathway selection).
- Stability & efficiency: Consistent output without fatigue or mood variation.
- Low-risk, high-cognitive execution: High-throughput checks of drug–drug interactions and contraindications; dose adjustments (hepatic/renal); guideline-pathway matching; appropriateness checks for ordered tests; first-read key-point extraction for reports; medical-record structuring; follow-up stratification and patient education drafting.

Physicians.

- Clinical judgment & ultimate responsibility: Integrate signs, disease course, and resource constraints to make safe, feasible decisions and bear ethical/legal accountability.
- Experience & uncertainty management: Handle atypical presentations, rare diseases, and reconciliation of conflicting information.
- Hands-on examinations & procedures: Physical exams (palpation, auscultation, localization of pain), procedures (puncture, suturing, catheterization, endoscopy, biopsy), and immediate interpretation/management of results.

Patients.

- First-hand information & preferences: Symptom nuances, lifestyle, and value trade-offs (efficacy vs. side effects, cost constraints).
- Execution & feedback: Adherence to medications/monitoring/lifestyle changes and timely reporting of discomfort and outcomes.

How DxDirector operationalizes complementarity. The system assigns the LLM the low-risk, cognitively intensive steps to offload clinician workload and accelerate care, while *dynamically escalating* to physicians whenever situations are high-risk, ethically sensitive (**Section 4.5**), or require examinations/procedures that only humans can perform. This division of labor increases efficiency and accuracy while preserving safety. The accountability framework further distinguishes and traces the inputs/outputs of the LLM and clinicians to ensure precise responsibility and reduce any chance of error propagation (**Section 2.8**).

References:

[1] Xu D, Chen W, Peng W, et al. Large language models for generative information extraction: A survey[J]. Frontiers of Computer Science, 2024, 18(6): 186357.

[2] Polak M P, Morgan D. Extracting accurate materials data from research papers with conversational language models and prompt engineering[J]. Nature Communications, 2024, 15(1): 1569.

[3] Al Shammari M, Hassan A, AlShamlan N, et al. Family medicine residents' skill levels in emergency chest X-ray interpretation[J]. BMC Family Practice, 2021, 22(1): 39.

[4] Andersen C A, Holden S, Vela J, et al. Point-of-care ultrasound in general practice: a systematic review[J]. The Annals of Family Medicine, 2019, 17(1): 61-69.

[5] Hickner J, Thompson P J, Wilkinson T, et al. Primary care physicians' challenges in ordering clinical laboratory tests and interpreting results[J]. The Journal of the American Board of Family Medicine, 2014, 27(2): 268-274.

[6] Houben P H H, van der Weijden T, Winkens B, et al. Pretest expectations strongly influence interpretation of abnormal laboratory results and further management[J]. BMC family practice, 2010, 11(1): 13.

[7] Casalino L P, Dunham D, Chin M H, et al. Frequency of failure to inform patients of clinically significant outpatient test results[J]. Archives of internal medicine, 2009, 169(12): 1123-1129.

[8] Cook D A, Oh S Y, Pusic M V. Accuracy of physicians' electrocardiogram interpretations: a systematic review and meta-analysis[J]. JAMA internal medicine, 2020, 180(11): 1461-1471.

Reviewer's Comment 4:

If the customized versions of the benchmarks, prompts, and agent code are available, it will enhance reproducibility.

Response 4:

Yes, as we stated in our code and data availability declaration in Section 5 and Section 6, we are willing to release the relevant information such as training data, benchmarks, prompts and code after the acceptance of this paper. We have already introduced the prompt in Section 2, 3 and 4 of Supplementary and uploaded the code in supplementary materials.

Reviewer's Comment 5:

Reviewer #4 (Remarks on code availability):

Code is not available.

Response 5:

To prompt the future research, we promise to release all source codes to train our DxDirector-7B on Github, and the model weights on Huggingface after the external review. This ensures unrestricted access for anyone to use the code and model weights for any purpose, fostering future research and development. Combined with our detailed introduction to the method and the open source datasets, we believe that researchers will find it very easy to reproduce our work. During the review, we have already provided the source code in supplementary materials.

Dear Editor and Reviewers,

Thank you very much for your positive feedback regarding our manuscript. We are delighted to learn that our previous responses and the revised manuscript have satisfactorily addressed the comments and concerns raised by the reviewers. We sincerely appreciate the time and effort the Editor and Reviewers have dedicated to evaluating our work. Your constructive suggestions have significantly improved the quality of our paper, thank you!

Reviewer #2:

Reviewer's Comment 1:

I thank the authors for their careful revisions. While they have satisfactorily addressed most of my comments, I do have one additional comment in response to their point-by-point.

With respect to your response to reviewer #2 comment 2, while I appreciate the inclusion of evaluation criteria, the criteria used seem somewhat subjective and would benefit from additional detail being listed in the manuscript.

Some examples of this include items such as: what is the definition of "consistent" how was the threshold between consistent/inconsistent defined? What is an "ambiguous" expression? How was "reducing the cognitive workload of clinicians" defined?

While the answers to these may seem obvious from a plain english perspective, this may differ between individual annotators. Additionally, some analysis of mismatches between inter-annotators may be beneficial.

Response 1:

We sincerely thank the reviewer for this constructive follow-up. We agree that while "plain English" descriptions provide a general overview, rigorous evaluation requires operationalized definitions to minimize subjective variance between annotators.

To address this, we have provided our description of the human evaluation protocol in the Supplementary Section 8:

1. Detailed Annotation Criteria and Thresholds

In our evaluation, the medical specialists were provided with the following specific operational definitions (now added to Supplementary Section 6):

Metric	High Score Criteria (9-10)	Low Score / Threshold Definitions (<5)
**1. Medical Accuracy**	Guideline Concordance: The reasoning aligns strictly with authoritative clinical guidelines (e.g., NCCN, AHA) and standard pathophysiology.	Factual Error Threshold: Contains specific hallucinations (e.g., non-existent drug interactions), incorrect dosing, or physiological inaccuracies.
**2. Logical Consistency**	Seamless Inference: Every diagnostic step logically follows from previous evidence. New hypotheses are derived only from established facts.	Inconsistency Threshold: Contains internal contradictions (e.g., noting "no fever" but later listing "febrile seizure" as a key factor) or logical leaps (jumping to conclusions without intermediate deduction).
**3. Clinical Appropriateness & Safety**	Contextualization: Recommendations are tailored to the patient's specific comorbidities and history. Risk Prioritization: Immediately identifies "Red Flag" symptoms.	Safety Threshold: Misses critical "Red Flags" (as defined in our High-Risk Escalation Policy); recommends contraindicated procedures; or provides generic advice that ignores specific patient constraints (e.g., renal failure).
**4. Clarity & Precision**	Standardized Terminology: Uses standard medical terminology (SNOMED-CT/ICD). Instructions are actionable and quantified (e.g., "monitor BP every	Ambiguity Threshold: Uses layperson terms (e.g., "bad headache" vs. "severe acute cephalgia") or vague instructions (e.g., "watch the patient" without specifying

**Metric**	**High Score Criteria (9-10)**	**Low Score / Threshold Definitions (<5)**
	4h").	parameters) that require physician clarification.
**5. Collaboration Efficiency**	Verification Mode: The output synthesizes information such that the physician only needs to *verify* and approve. Reduces cognitive load.	Re-work Mode: The output is useless, disorganized or verbose, forcing the physician to re-read raw case files or re-derive the reasoning from scratch to ensure safety.

2. Analysis of Inter-Annotator Mismatches

We have further analyzed the inter-rater reliability (Intraclass Correlation Coefficient (ICC)=0.85), which shows consistent agreement across reviewers, ensuring the robustness of the human evaluation process. Besides, we found that disagreements primarily stemmed from two sources:

- Clinical "Gray Zones" (Subjectivity): In complex cases (e.g., autoimmune diseases with atypical presentation), specialists occasionally differed on whether a specific diagnostic test was "essential" (part of the optimal reasoning path) or "optional" (defensive medicine), leading to differences in the "Logical Consistency" score.
- Stylistic Preferences: Some mismatches in "Ambiguity" scores arose from preferences for specific medical phrasing (e.g., "unremarkable" vs. "normal") rather than actual semantic confusion.

We have updated the Supplementary Materials (Section 8) to include the detailed scoring rubrics table. We believe these operational definitions provide the transparency and rigor requested.

Reviewer #4:

Reviewer's Comment 1:

I reviewed authors' response specifically to my comments and questions as well as in the

manuscript. I think the paper is acceptable for publication now.

Response 1:

We thank the reviewer for your time in reviewing our responses and the revised manuscript. We appreciate the positive evaluation and the recommendation for publication. Thank you!
